# Supplementary figures and images for: An augmented Mendelian randomization approach provides causality of brain imaging features on complex traits in a single biobank-scale dataset
Source: PLoS Genet. 2023 Dec 27;19(12):e1011112. doi: 10.1371/journal.pgen.1011112 (PMC10775988; doi:10.1371/journal.pgen.1011112)

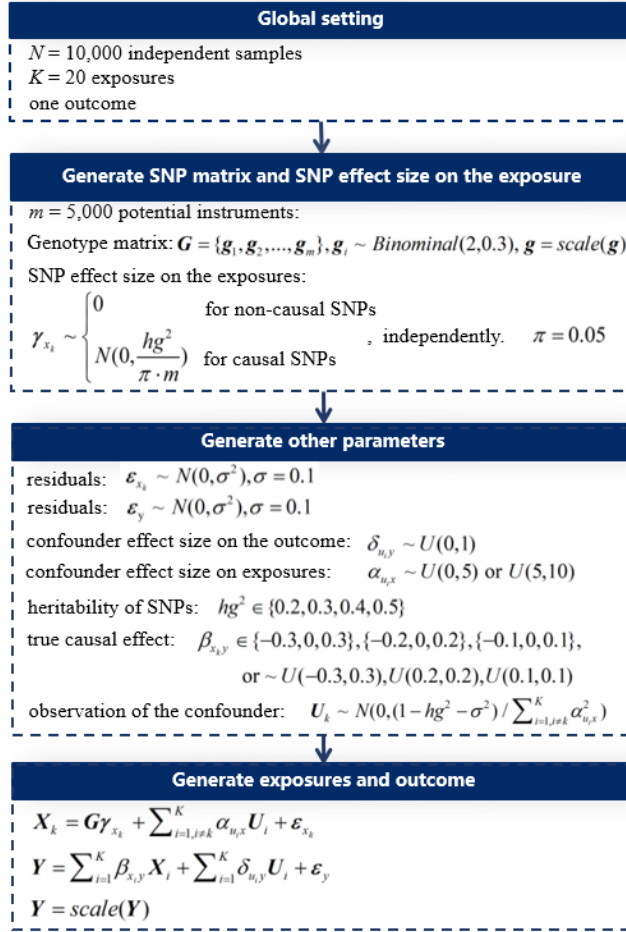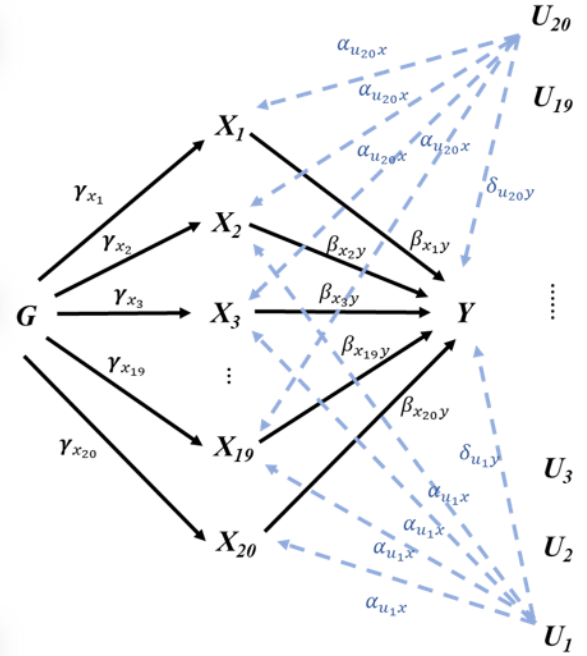

**S1 Fig. The data generation framework in baseline simulation.**

Supplement: S1 Fig — (PDF) [file pgen.1011112.s001.pdf]

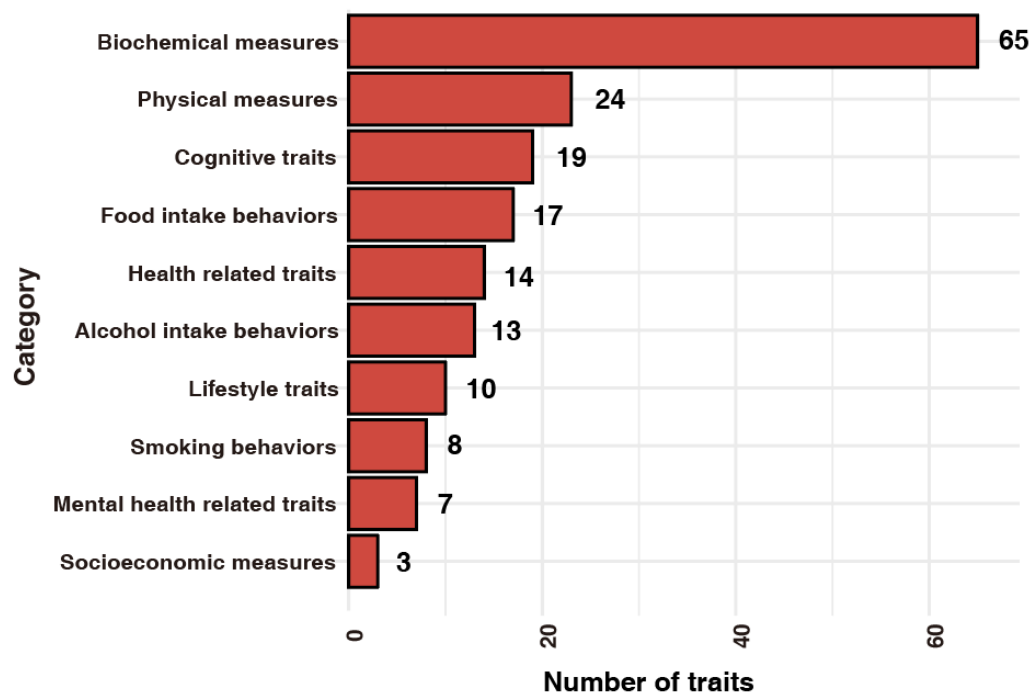

**S25 Fig.** Total number of traits in each trait categories from the UK Biobank.

Supplement: S25 Fig — (PDF) [file pgen.1011112.s025.pdf]
