# Supplementary material for: An augmented Mendelian randomization approach provides causality of brain imaging features on complex traits in a single biobank-scale dataset
Source: PLoS Genet. 2023 Dec 27;19(12):e1011112. doi: 10.1371/journal.pgen.1011112 (PMC10775988; doi:10.1371/journal.pgen.1011112)
Supplement: S2 Fig — Bias was calculated as the mean of the absolute difference between causal estimate and true effect, and variance was calculated as the variance of causal estimate minus true effect (averaged across 100 replications for each setting). The results from the methods of ImagingMR and PRS-2SLS were not included here. The full results can be found in S3 Table. (PDF) [file pgen.1011112.s002.pdf]

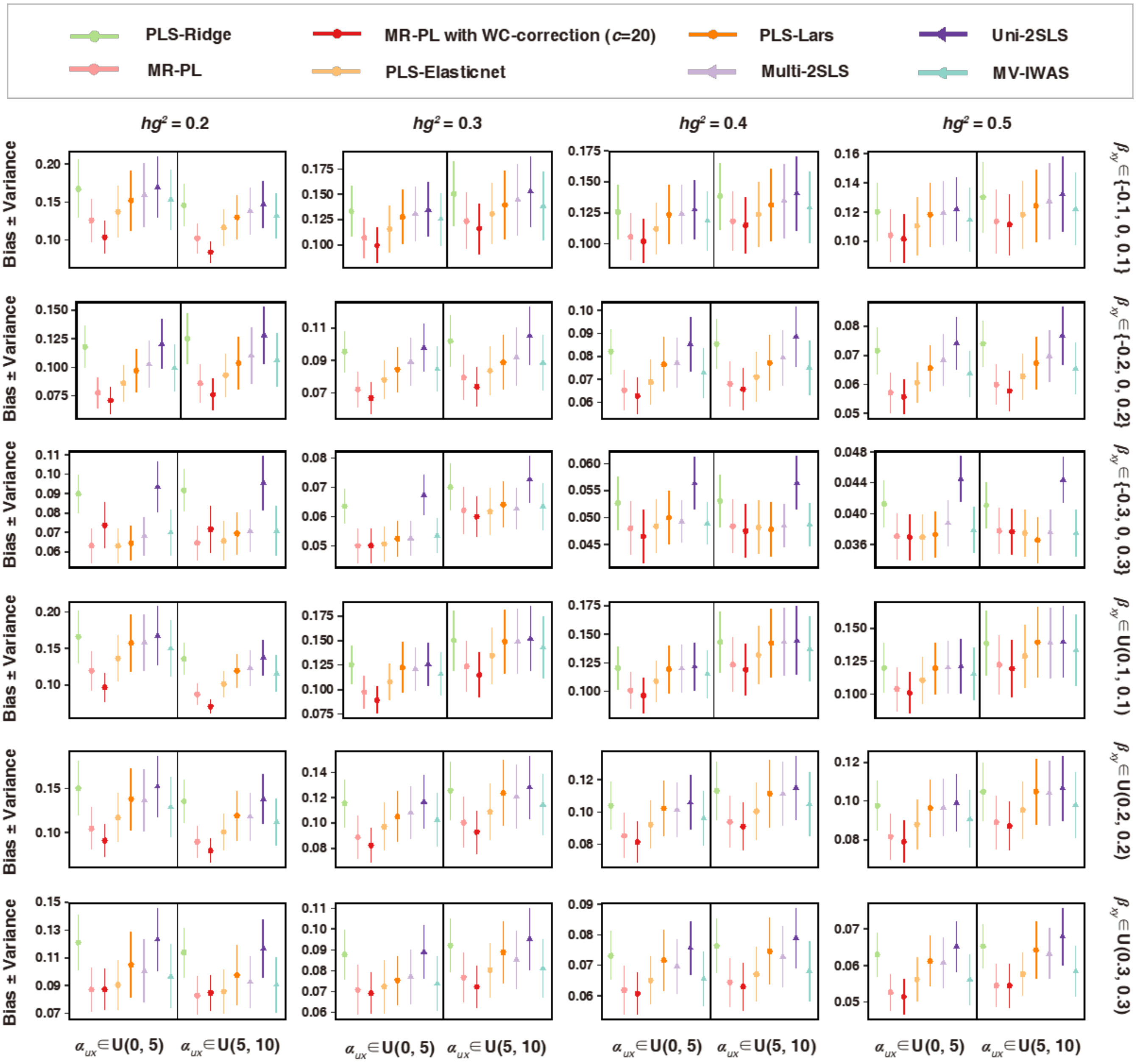

S2 Fig. Bias and variance results of MR-PL and other MR approaches in baseline simulation. Bias was calculated as the mean of the absolute difference between causal estimate and true effect, and variance was calculated as the variance of causal estimate minus true effect (averaged across 100 replications for each setting). The results from the methods of ImagingMR and PRS-2SLS were not included here. The full results can be found in **S3 Table**.
