# Supplementary material for: An augmented Mendelian randomization approach provides causality of brain imaging features on complex traits in a single biobank-scale dataset
Source: PLoS Genet. 2023 Dec 27;19(12):e1011112. doi: 10.1371/journal.pgen.1011112 (PMC10775988; doi:10.1371/journal.pgen.1011112)
Supplement: S3 Fig — Simulation settings were included if the causal effect was drawn from the discrete set. MR approaches were included if individual P-value of causal effect estimate of each exposure can be calculated. A method fails to control type I error if its type I error rate exceeds the nominal significance level of 0.05 (red dotted line). The error bar represents the variance of type I error rate over 100 replications. For settings with too small variance, the error bar tends to degenerate to a point. (PDF) [file pgen.1011112.s003.pdf]

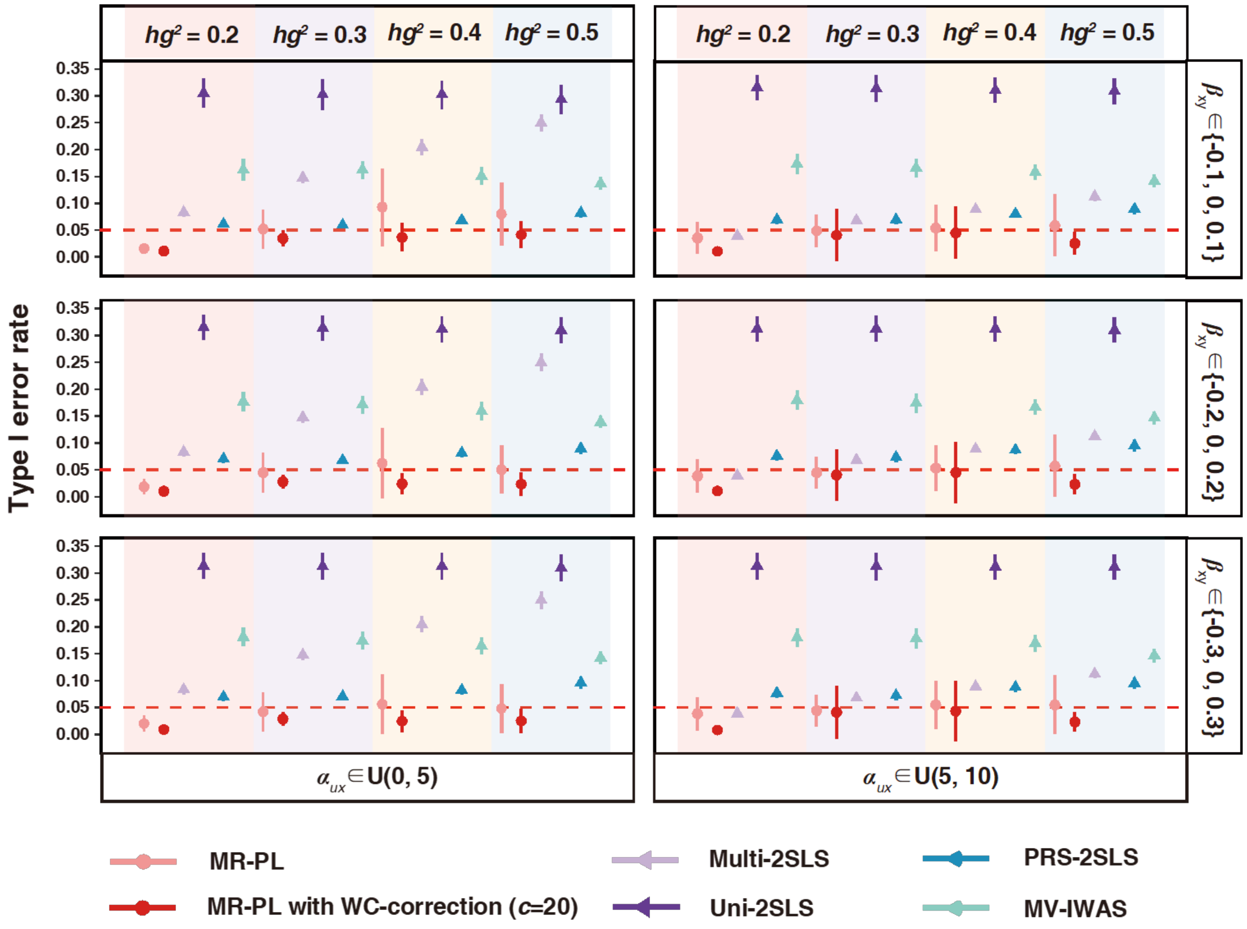

S3 Fig. Type I error rate of MR-PL and other MR approaches in supplementary simulation with the presence of linkage disequilibrium. Simulation settings were included if the causal effect was drawn from the discrete set. MR approaches were included if individual *P*-value of causal effect estimate of each exposure can be calculated. A method fails to control type I error if its type I error rate exceeds the nominal significance level of 0.05 (red dotted line). The error bar represents the variance of type I error rate over 100 replications. For settings with too small variance, the error bar tends to degenerate to a point.
