# Supplementary material for: An augmented Mendelian randomization approach provides causality of brain imaging features on complex traits in a single biobank-scale dataset
Source: PLoS Genet. 2023 Dec 27;19(12):e1011112. doi: 10.1371/journal.pgen.1011112 (PMC10775988; doi:10.1371/journal.pgen.1011112)
Supplement: S4 Fig — Simulation settings were included if the causal effect was drawn from the discrete set. A method fails to control type I error if its type I error rate exceeds the nominal significance level of 0.05 (red dotted line). The error bar represents the variance of type I error rate over 100 replications. (PDF) [file pgen.1011112.s004.pdf]

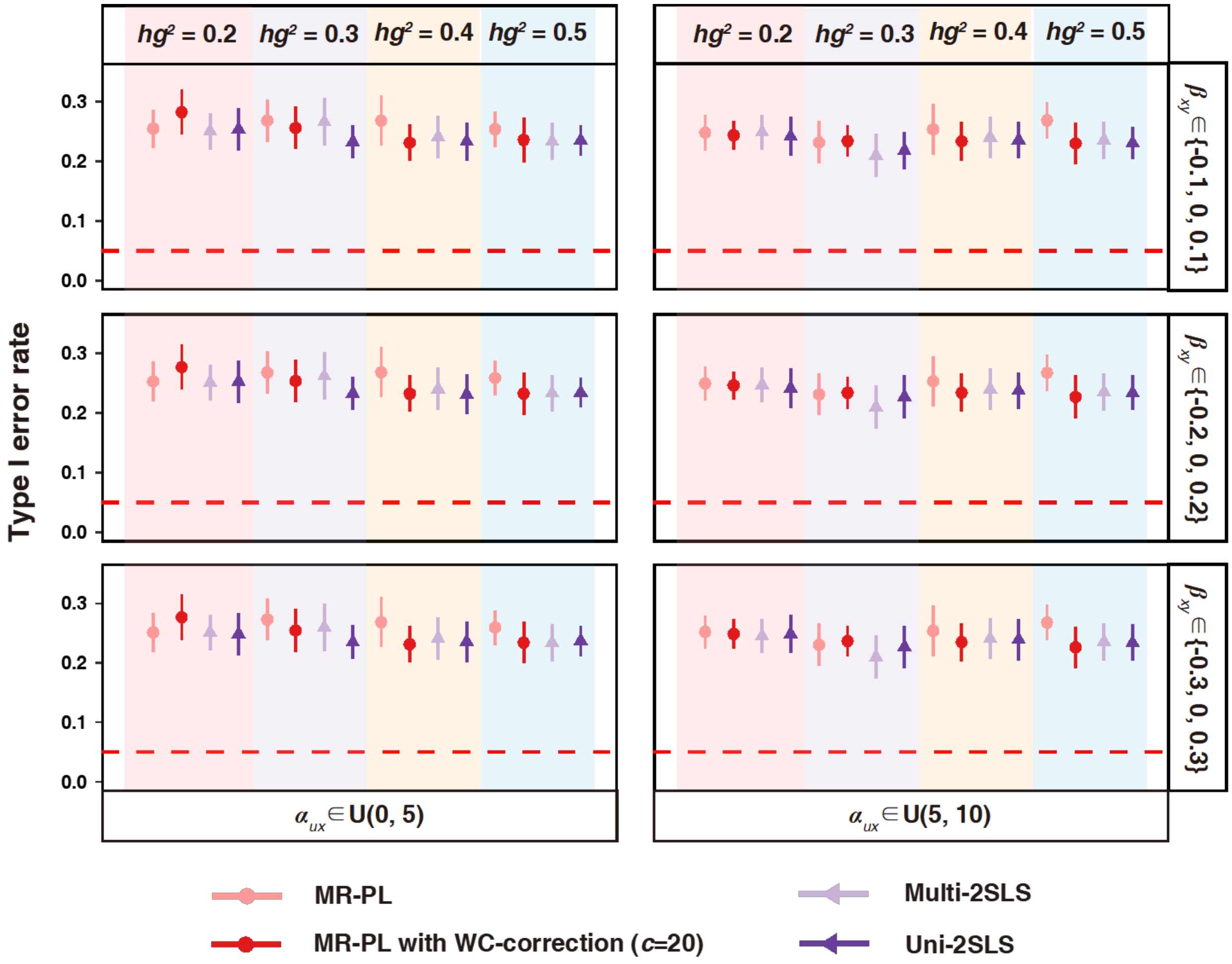

S4 Fig. Type I error rate of MR-PL and other MR approaches in supplementary simulation with the presence of pleiotropy. Simulation settings were included if the causal effect was drawn from the discrete set. A method fails to control type I error if its type I error rate exceeds the nominal significance level of 0.05 (red dotted line). The error bar represents the variance of type I error rate over 100 replications.
