# Supplementary material for: An augmented Mendelian randomization approach provides causality of brain imaging features on complex traits in a single biobank-scale dataset
Source: PLoS Genet. 2023 Dec 27;19(12):e1011112. doi: 10.1371/journal.pgen.1011112 (PMC10775988; doi:10.1371/journal.pgen.1011112)
Supplement: S5 Fig — A histogram illustrating the distribution of P-values across 100 replications is presented for each parameter setting. A P-value exceeding 0.05 indicates the absence of pleiotropy. (PDF) [file pgen.1011112.s005.pdf]

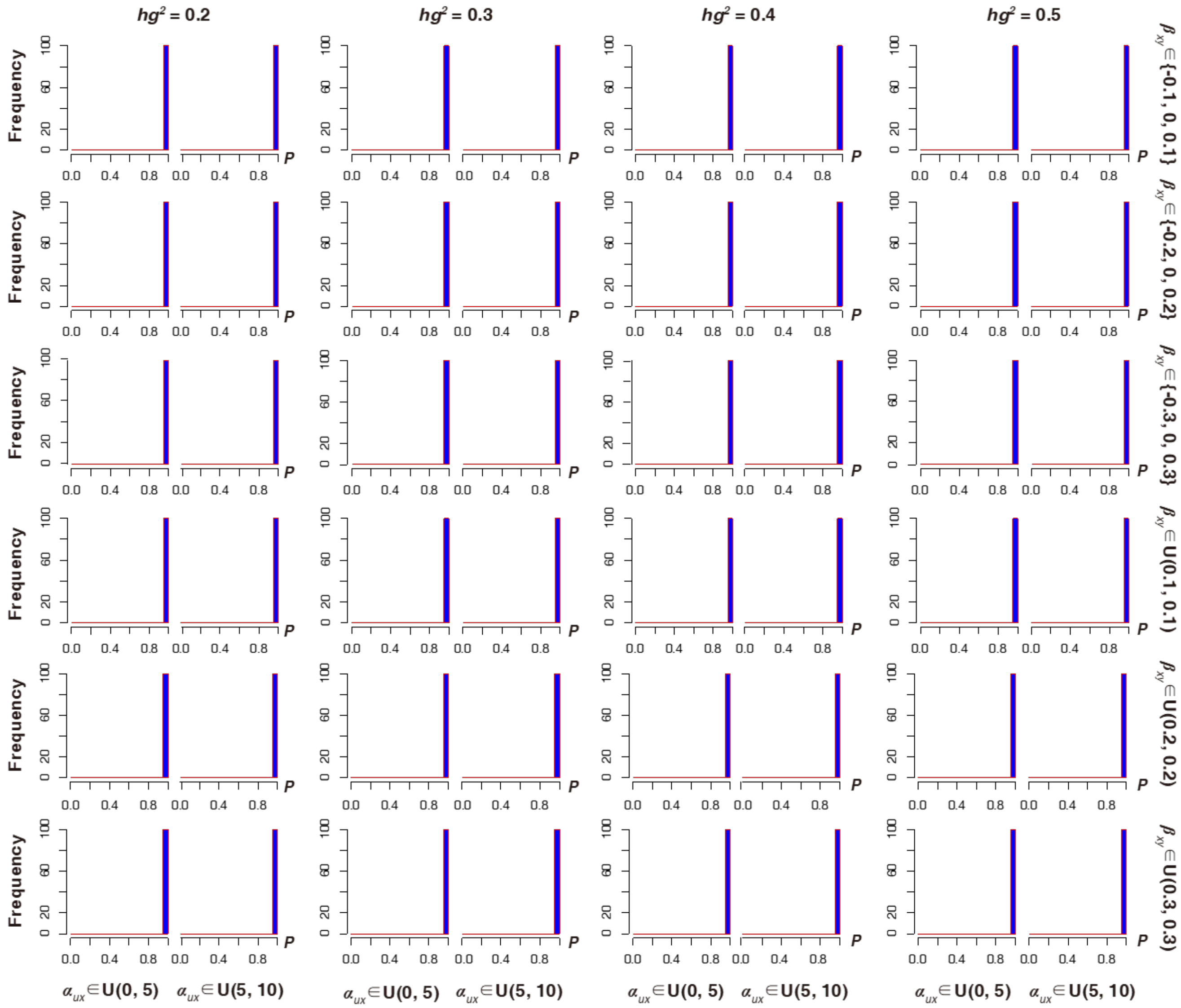

S5 Fig. Sargan test *P*-value distribution of MR-PL in baseline simulation where no pleiotropy exists. A histogram illustrating the distribution of *P*-values across 100 replications is presented for each parameter setting. A *P*-value exceeding 0.05 indicates the absence of pleiotropy.
