# Supplementary material for: An augmented Mendelian randomization approach provides causality of brain imaging features on complex traits in a single biobank-scale dataset
Source: PLoS Genet. 2023 Dec 27;19(12):e1011112. doi: 10.1371/journal.pgen.1011112 (PMC10775988; doi:10.1371/journal.pgen.1011112)
Supplement: S8 Fig — A histogram illustrating the distribution of P-values across 100 replications is presented for each parameter setting. A P-value below 0.05 indicates the presence of pleiotropy. (PDF) [file pgen.1011112.s008.pdf]

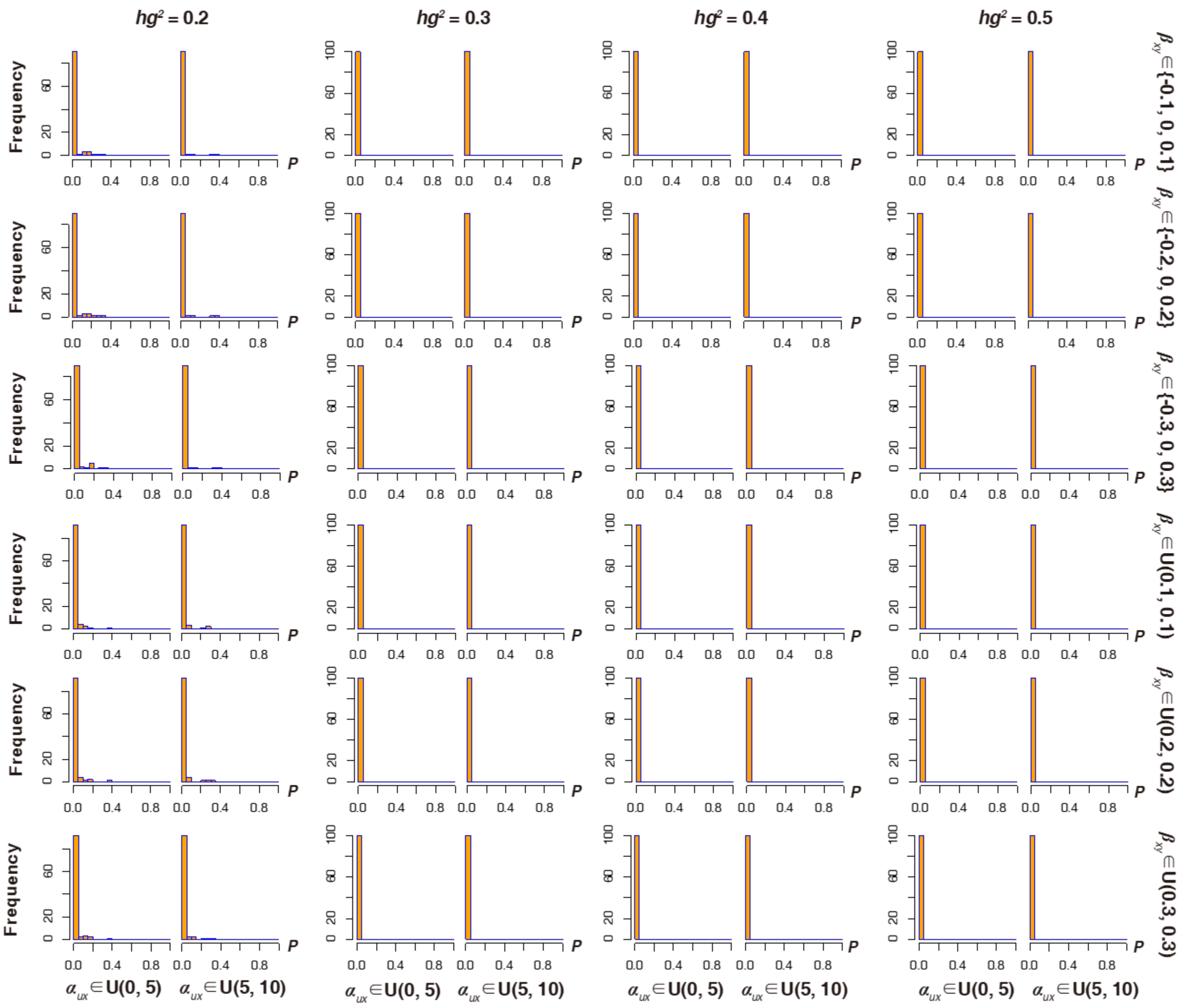

S8 Fig. Sargan test *P*-value distribution of MR-PL with WC-correction in supplementary simulation with the presence of pleiotropy. A histogram illustrating the distribution of *P*-values across 100 replications is presented for each parameter setting. A *P*-value below 0.05 indicates the presence of pleiotropy.
