# Supplementary material for: An augmented Mendelian randomization approach provides causality of brain imaging features on complex traits in a single biobank-scale dataset
Source: PLoS Genet. 2023 Dec 27;19(12):e1011112. doi: 10.1371/journal.pgen.1011112 (PMC10775988; doi:10.1371/journal.pgen.1011112)
Supplement: S9 Fig — Simulation settings were included if the causal effect was drawn from the discrete sets. The error bar represents the variance of power over 100 replications in each parameter setting. For settings with too small variance, the error bar tends to degenerate to a point. (PDF) [file pgen.1011112.s009.pdf]

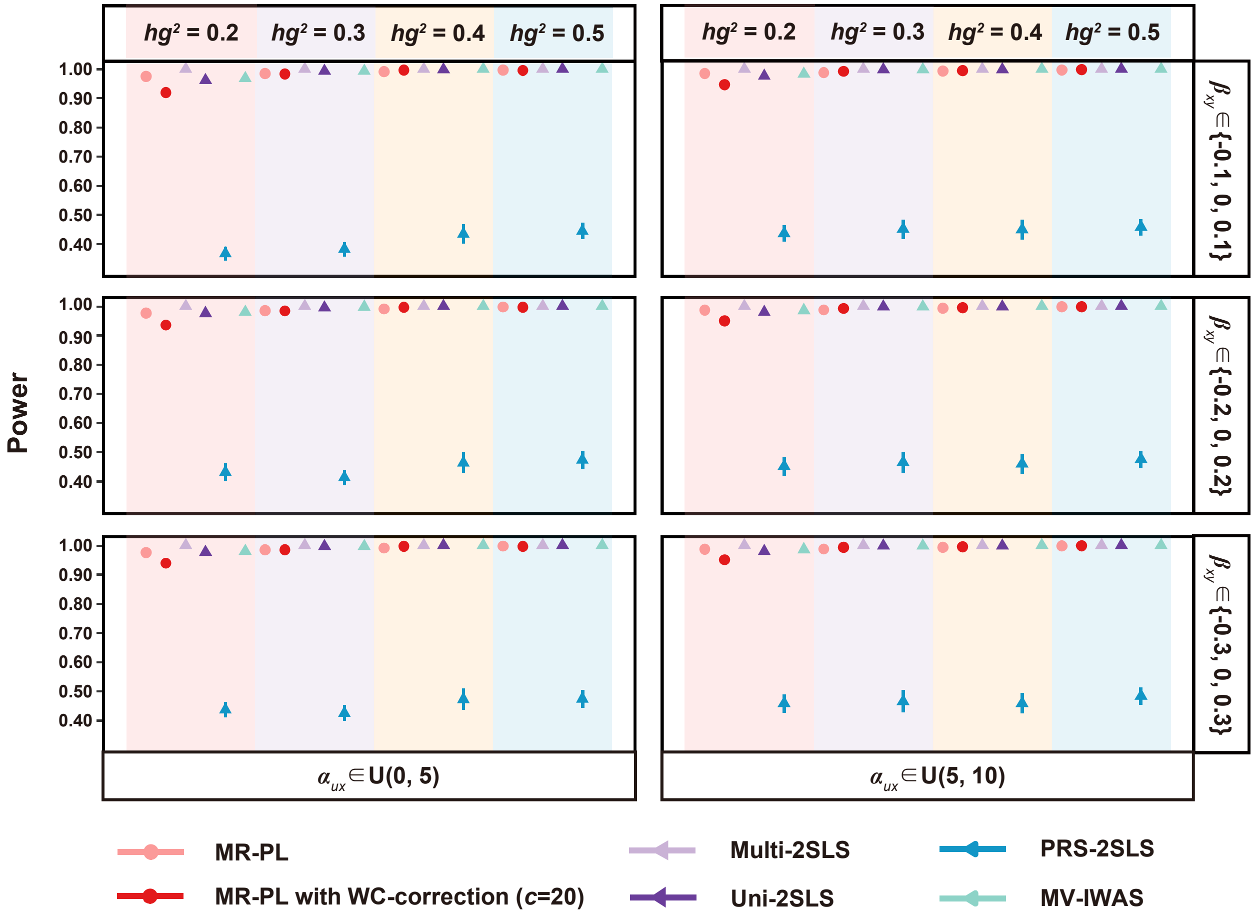

S9 Fig. Power results of MR-PL and other MR approaches in baseline simulation. Simulation settings were included if the causal effect was drawn from the discrete sets. The error bar represents the variance of power over 100 replications in each parameter setting. For settings with too small variance, the error bar tends to degenerate to a point.
