# Supplementary material for: An augmented Mendelian randomization approach provides causality of brain imaging features on complex traits in a single biobank-scale dataset
Source: PLoS Genet. 2023 Dec 27;19(12):e1011112. doi: 10.1371/journal.pgen.1011112 (PMC10775988; doi:10.1371/journal.pgen.1011112)
Supplement: S11 Fig — The error bar represents ten-fold variance in MSE over 100 replications in each parameter setting. For settings with too small variance, the error bar tends to degenerate to a point. (PDF) [file pgen.1011112.s011.pdf]

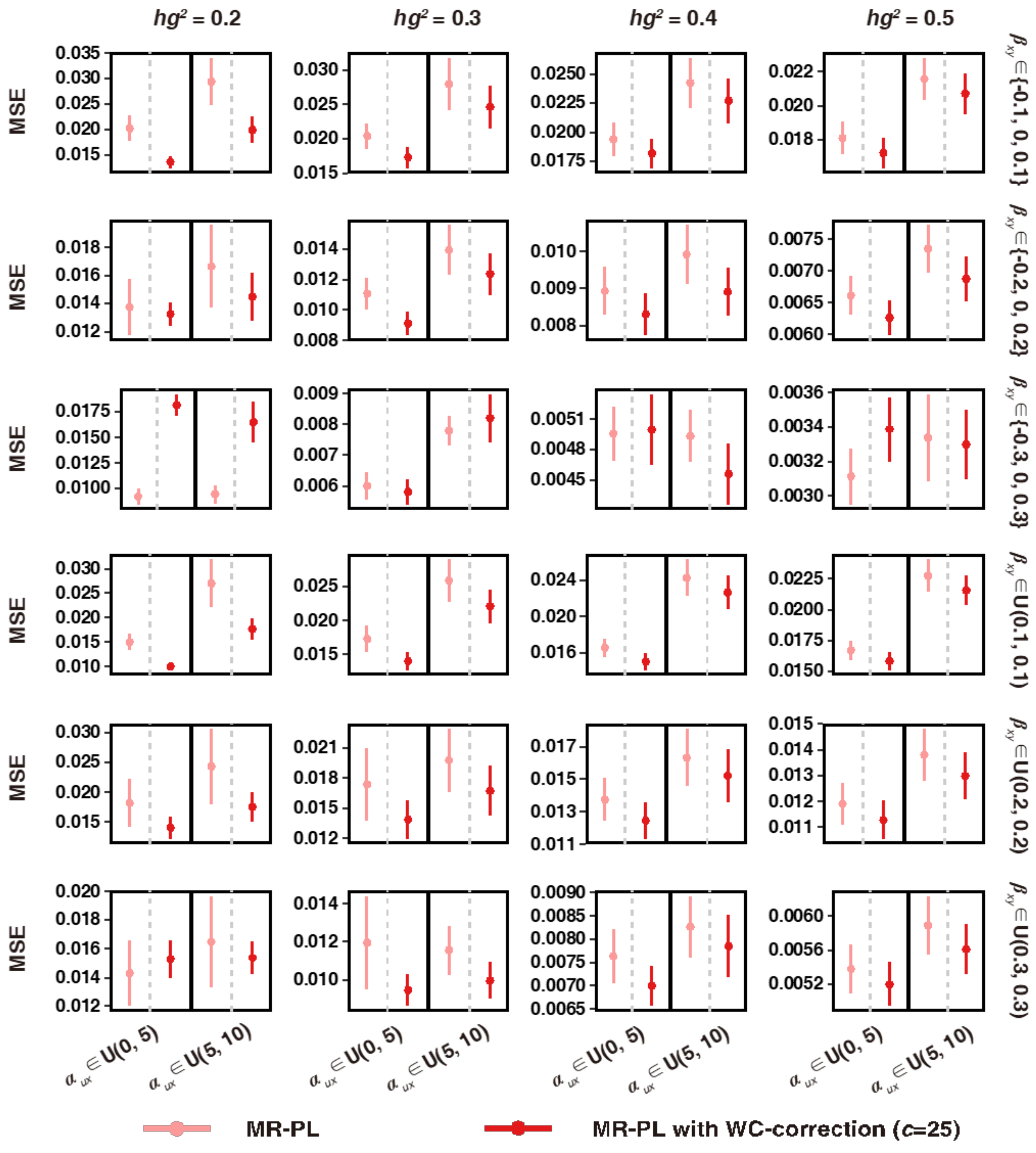

S11 Fig. A comparison of mean squared error (MSE) between MR-PL with and without winner's curse correction (WC-correction) at *c*=25 in baseline simulation. The error bar represents ten-fold variance in MSE over 100 replications in each parameter setting. For settings with too small variance, the error bar tends to degenerate to a point.
