# Supplementary material for: An augmented Mendelian randomization approach provides causality of brain imaging features on complex traits in a single biobank-scale dataset
Source: PLoS Genet. 2023 Dec 27;19(12):e1011112. doi: 10.1371/journal.pgen.1011112 (PMC10775988; doi:10.1371/journal.pgen.1011112)
Supplement: S13 Fig — Simulation settings were included if the causal effect was drawn from the discrete set. The error bar represents the variance of type I error rate across 100 replications for each parameter setting. For settings with too small variance, the error bar tends to degenerate to a point. (PDF) [file pgen.1011112.s013.pdf]

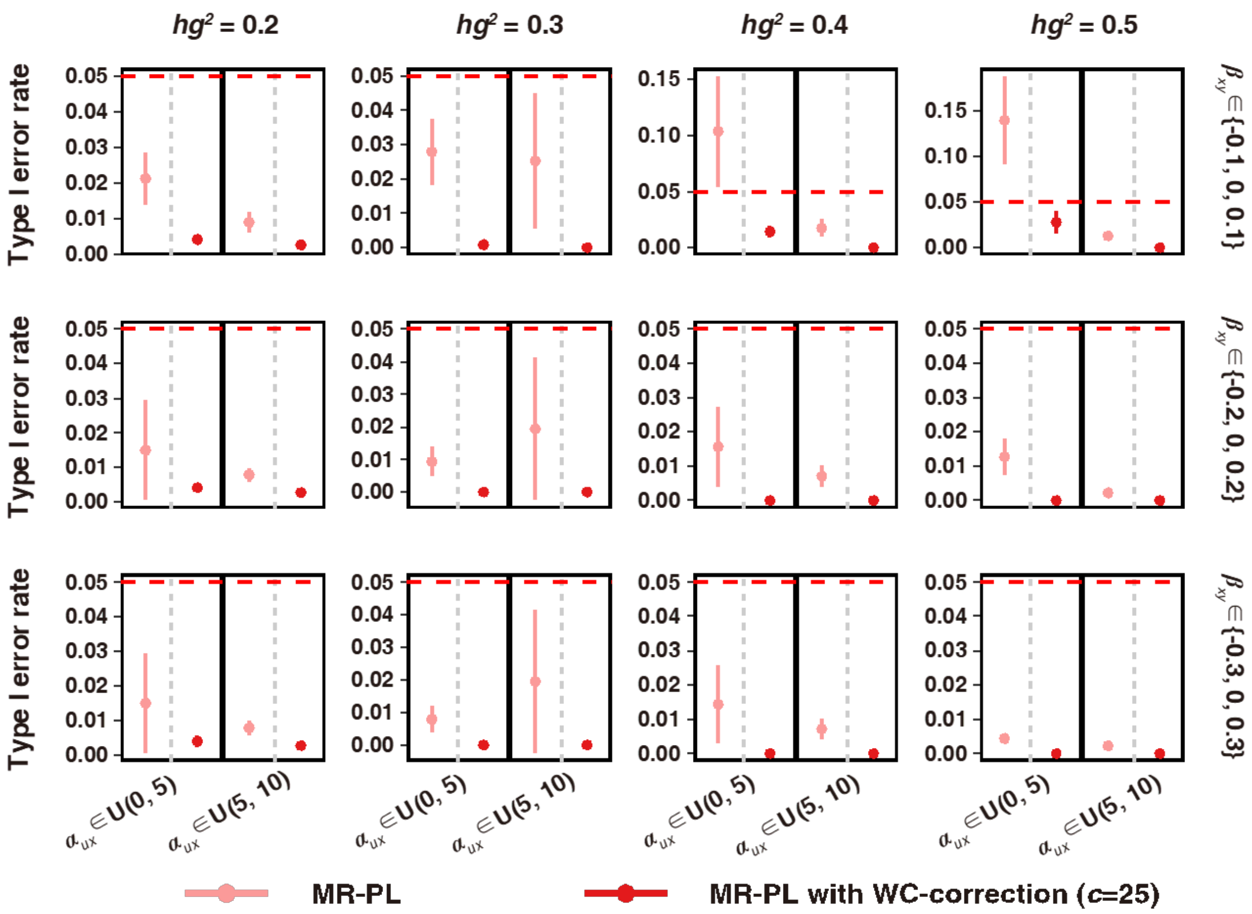

S13 Fig. A comparison of type I error rate between MR-PL with and without winner's curse correction (WC-correction) at c=25 in baseline simulation. Simulation settings were included if the causal effect was drawn from the discrete set. The error bar represents the variance of type I error rate across 100 simulations for each parameter setting. For settings with too small variance, the error bar tends to degenerate to a point.
