# Supplementary material for: An augmented Mendelian randomization approach provides causality of brain imaging features on complex traits in a single biobank-scale dataset
Source: PLoS Genet. 2023 Dec 27;19(12):e1011112. doi: 10.1371/journal.pgen.1011112 (PMC10775988; doi:10.1371/journal.pgen.1011112)
Supplement: S14 Fig — Simulation settings were included if the causal effect was drawn from the discrete set. The error bar represents the variance of power across 100 replications for each parameter setting. For settings with too small variance, the error bar tends to degenerate to a point. (PDF) [file pgen.1011112.s014.pdf]

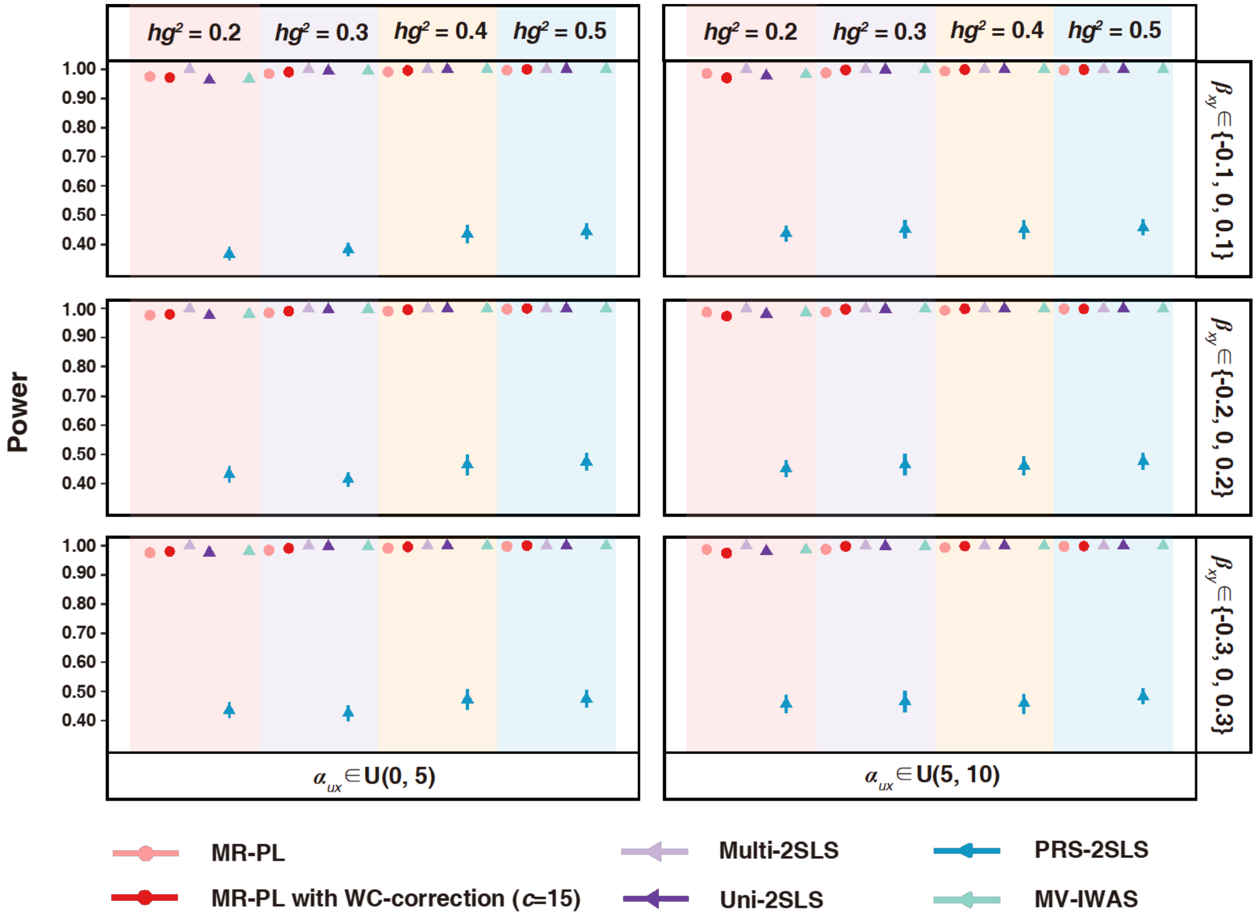

S14 Fig. A comparison of power results between MR-PL with and without winner's curse correction (WC-correction) at *c*=15 (and other approaches) in baseline simulation. Simulation settings were included if the causal effect was drawn from the discrete set. The error bar represents the variance of power across 100 simulations for each parameter setting. For settings with too small variance, the error bar tends to degenerate to a point.
