# Supplementary material for: An augmented Mendelian randomization approach provides causality of brain imaging features on complex traits in a single biobank-scale dataset
Source: PLoS Genet. 2023 Dec 27;19(12):e1011112. doi: 10.1371/journal.pgen.1011112 (PMC10775988; doi:10.1371/journal.pgen.1011112)
Supplement: S17 Fig — Simulation settings are included if the causal effect was drawn from the discrete set. The error bar represents the variance of type I error rate across 100 replications for each parameter setting. For settings with too small variance, the error bar tends to degenerate to a point. (PDF) [file pgen.1011112.s017.pdf]

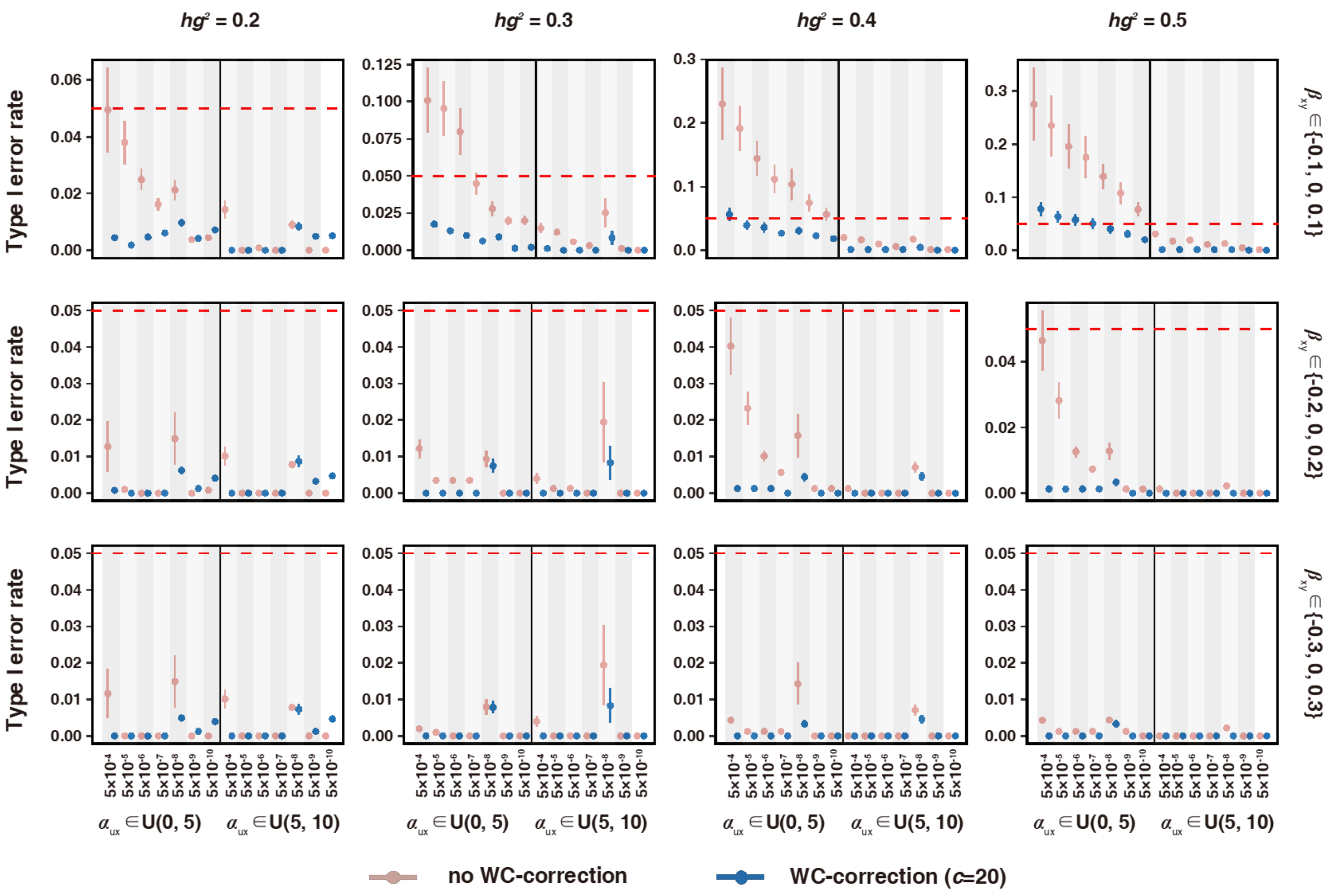

S17 Fig. A comparison of type I error rate between MR-PL with and without winner's curse correction (WC-correction) across different *P*-value thresholds to select instrumental variants in baseline simulation. Simulation settings are included if the causal effect was drawn from the discrete set. The error bar represents the variance of type I error rate across 100 simulations for each parameter setting. For settings with too small variance, the error bar tends to degenerate to a point.
