# Supplementary material for: An augmented Mendelian randomization approach provides causality of brain imaging features on complex traits in a single biobank-scale dataset
Source: PLoS Genet. 2023 Dec 27;19(12):e1011112. doi: 10.1371/journal.pgen.1011112 (PMC10775988; doi:10.1371/journal.pgen.1011112)
Supplement: S22 Fig — The error bar represents ten-fold variance in the MSE over 100 replications in each parameter setting. For settings with too small variance, the error bar tends to degenerate to a point. (PDF) [file pgen.1011112.s022.pdf]

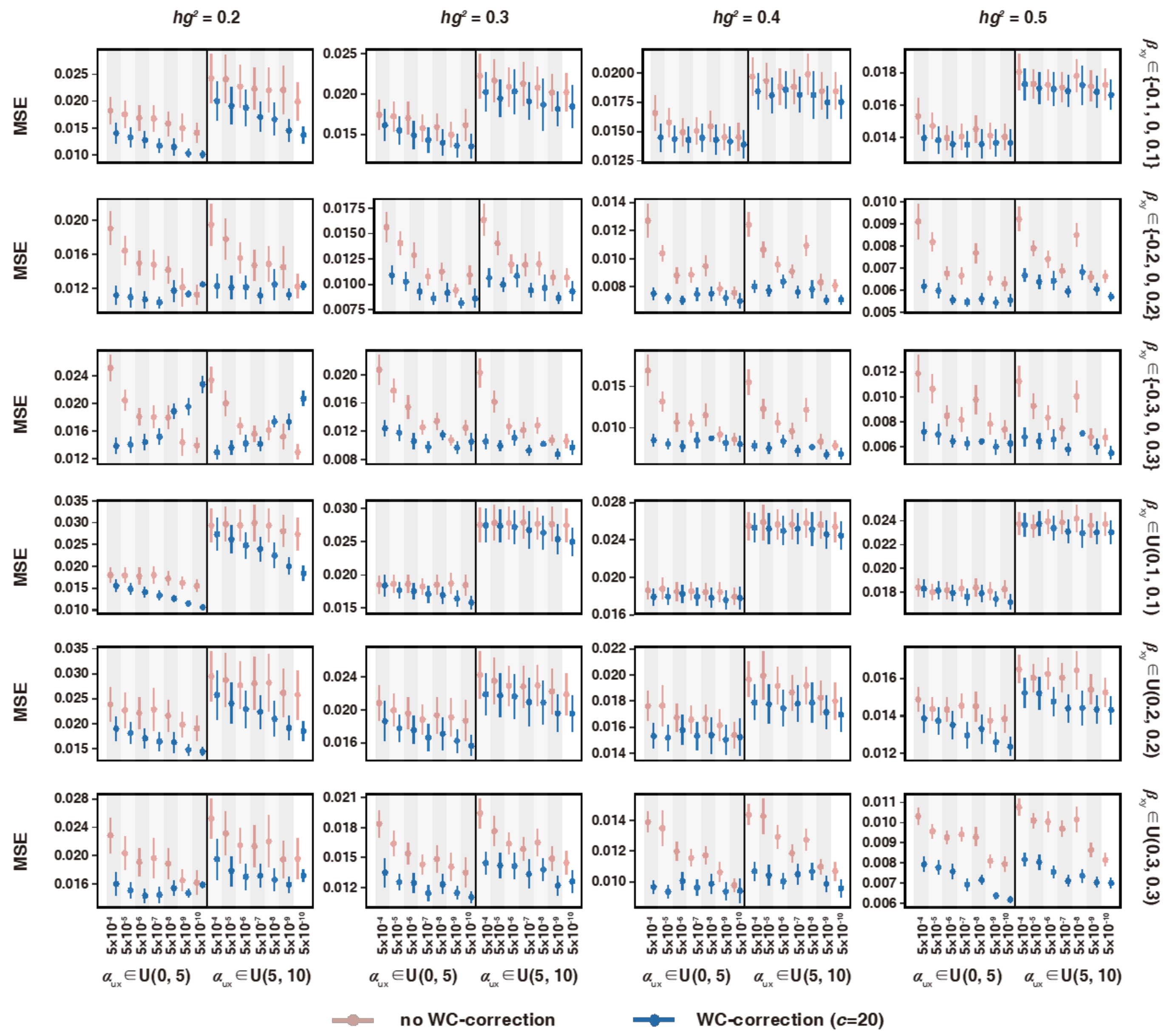

S22 Fig. A comparison of mean squared error (MSE) between MR-PL with and without winner's curse correction (WC-correction) across different *P*-value thresholds to select instrumental variants in supplementary simulation with the presence of linkage disequilibrium. The error bar represents ten-fold variance in the MSE over 100 replications in each parameter setting. For settings with too small variance, the error bar tends to degenerate to a point.
