# Supplementary material for: An augmented Mendelian randomization approach provides causality of brain imaging features on complex traits in a single biobank-scale dataset
Source: PLoS Genet. 2023 Dec 27;19(12):e1011112. doi: 10.1371/journal.pgen.1011112 (PMC10775988; doi:10.1371/journal.pgen.1011112)
Supplement: S24 Fig — (A) Boxplot of the conditional F-statistics before and after WC-correction, with a mean of 4.37 before WC-correction, and 5.63 after WC-correction. (B, D) Scatter plots colored by density of the conditional F-statistics and exposure numbers across 43,200 simulations (100 replications × 48 settings × 9 c values), where (D) displays separate plots for different parameter settings of heritability. (C, E) Scatter plots colored by density of conditional F-statistics and instrumental SNP numbers across 43,200 simulations (100 replications × 48 settings × 9 c values), where (E) displays separate plots for different parameter settings of heritability. (F) A comparison of Mean squared error (MSE) for MR-PL across different bins of conditional F-statistic values. The error bar represents the variance of MSE. (E) A comparison of type I error rate for MR-PL across different bins of conditional F-statistic values. The error bar represents the variance of type I error rate. The red dotted line denotes the rule-of-thumb value of 10. r: the estimate of Spearman correlation; P: the P-value of Spearman correlation test; n_neighbors: the number of dots around each dot. (PDF) [file pgen.1011112.s024.pdf]

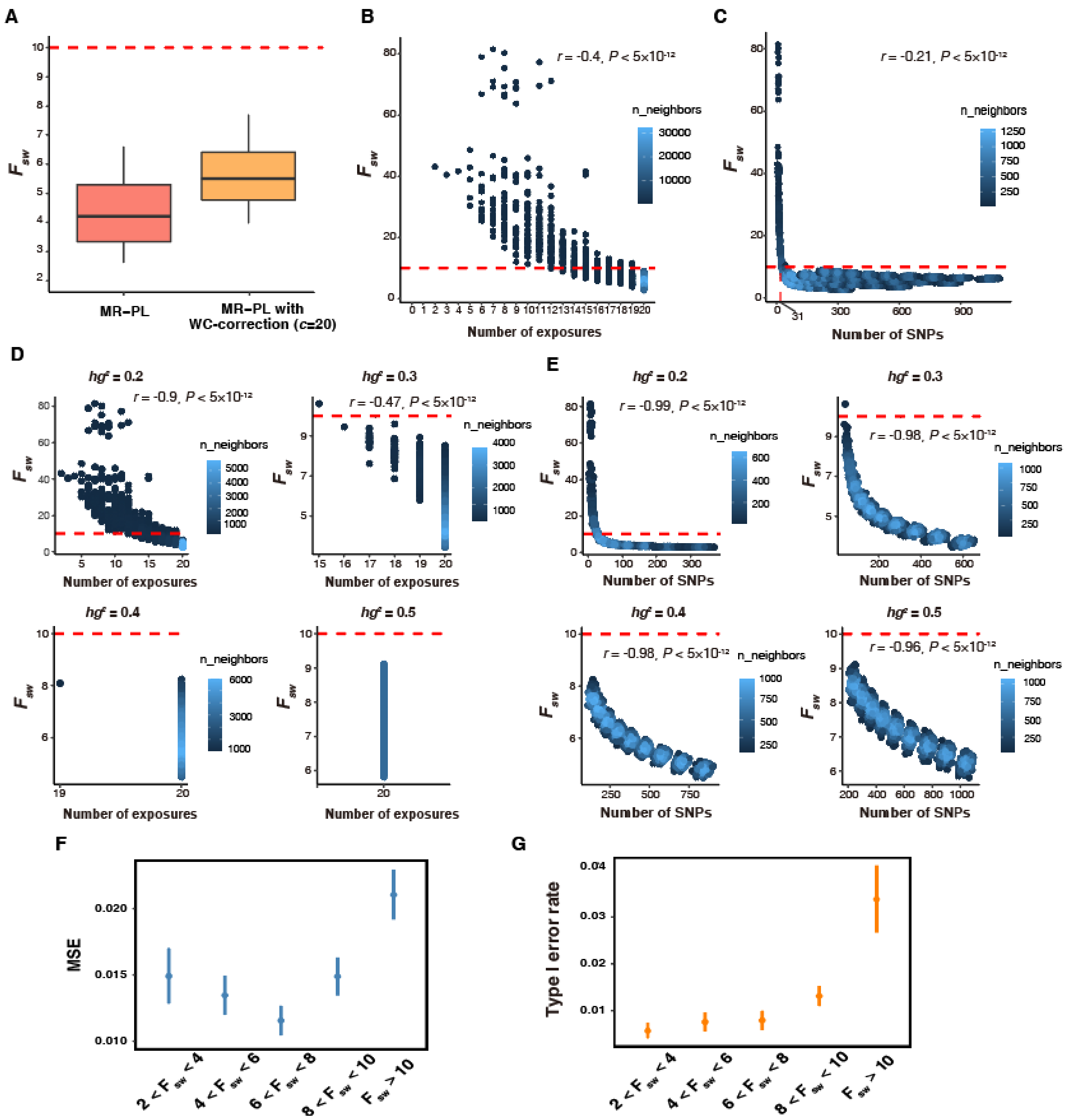

S24 Fig. Results of conditional F-statistic values (F_{sw}) in baseline simulation. (A) Boxplot of the conditional F-statistics before and after WC-correction, with a mean of 4.37 before WC-correction, and 5.63 after WC-correction. **(B, D)** Scatter plots colored by density of the conditional F-statistics and exposure numbers across 43,200 simulations (100 replications × 48 settings × 9 *c* values), where **(D)** displays separate plots for different parameter settings of heritability. **(C, E)** Scatter plots colored by density of conditional F-statistics and instrumental SNP numbers across 43,200 simulations (100 replications × 48 settings × 9 *c* values), where **(E)** displays separate plots for different parameter settings of heritability. **(F)** A comparison of Mean squared error (MSE) for MR-PL across different bins of conditional F-statistic values. The error bar represents the variance of MSE. **(E)** A comparison of type I error rate for MR-PL across different bins of conditional F-statistic values. The error bar represents the variance of type I error rate. The red dotted line denotes the rule-of-thumb value of 10. *r*: the estimate of Spearman correlation; *P*: the *P*-value of Spearman correlation test; n_neighbors: the number of dots around each dot.
