# Supplementary material for: An augmented Mendelian randomization approach provides causality of brain imaging features on complex traits in a single biobank-scale dataset
Source: PLoS Genet. 2023 Dec 27;19(12):e1011112. doi: 10.1371/journal.pgen.1011112 (PMC10775988; doi:10.1371/journal.pgen.1011112)
Supplement: S26 Fig — Each dot represents the percentage of non-causal SNPs selected as IVs under a specific parameter setting (number of non-causal SNPs selected as instruments / total number of non-causal SNPs), averaged across 100 replications. The Wilcoxon signed rank test was used for testing the difference of the percentage before and after WC-correction across all 48 parameter settings. It showed that the WC-correction procedure could significantly reduce the chances for non-causal SNPs chosen as IVs which would otherwise bias MR results greatly. (PDF) [file pgen.1011112.s026.pdf]

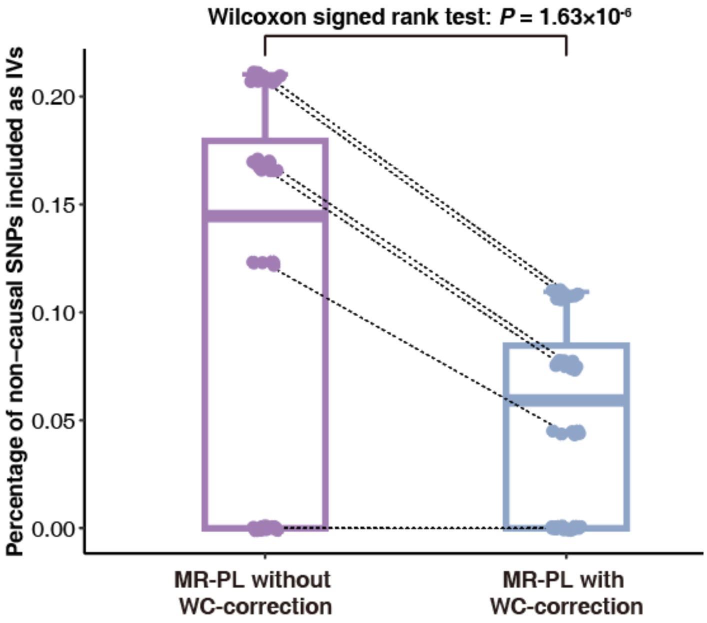

S26 Fig. Percentage of non-causal SNPs selected as IVs before and after WC-correction. Each dot represents the percentage of non-causal SNPs selected as IVs under a specific parameter setting (number of non-causal SNPs selected as instruments / total number of non-causal SNPs), averaged across 100 replications. The Wilcoxon signed rank test was used for testing the difference of the percentage before and after WC-correction across all 48 parameter settings. It showed that the WC-correction procedure could significantly reduce the chances for non-causal SNPs chosen as IVs which would otherwise bias MR results greatly.
