# Supplementary material for: An augmented Mendelian randomization approach provides causality of brain imaging features on complex traits in a single biobank-scale dataset
Source: PLoS Genet. 2023 Dec 27;19(12):e1011112. doi: 10.1371/journal.pgen.1011112 (PMC10775988; doi:10.1371/journal.pgen.1011112)
Supplement: S27 Fig — (A) Correlation between the mean fractional anisotropy (FA) of white matter tracts before regressing out the covariates. (B) Correlation between the mean FA value of white matter tracts after regressing out the covariates. The complete names of these white matter tracts that correspond to the abbreviation used can be found in S7 Table. (PDF) [file pgen.1011112.s027.pdf]

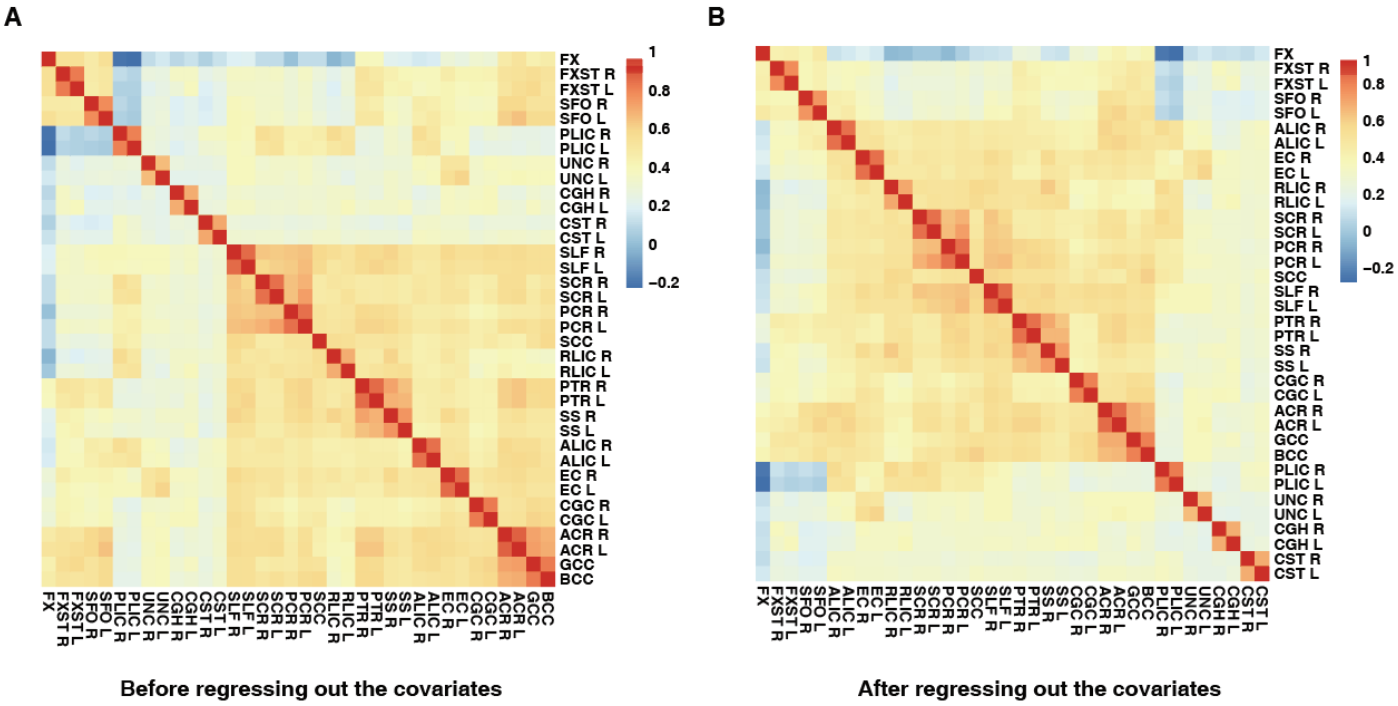


**S27 Fig. Correlation between different white matter tracts. (A)** Correlation between the mean fractional anisotropy (FA) of white matter tracts before regressing out the covariates. **(B)** Correlation between the mean FA value of white matter tracts after regressing out the covariates. The complete names of these white matter tracts that correspond to the abbreviation used can be found in **S7 Table**.
