# Supplementary material for: An augmented Mendelian randomization approach provides causality of brain imaging features on complex traits in a single biobank-scale dataset
Source: PLoS Genet. 2023 Dec 27;19(12):e1011112. doi: 10.1371/journal.pgen.1011112 (PMC10775988; doi:10.1371/journal.pgen.1011112)
Supplement: S1 Text — (PDF) [file pgen.1011112.s051.pdf]

S1 Text. Supplementary Methods

Supplementary simulation with instrumental SNPs in linkage disequilibrium

For this supplementary simulation, we extracted the genotype on chromosome 22 from the UK Biobank. The quality control of genotype was performed using PLINK v1.9 through the following steps: 1) removal of SNPs with minor allele frequency < 1%; 2) removal of SNPs with missing samples > 20%; 3) removal of samples with missing genotypes > 20%; 4) removal of SNPs deviating from Hardy–Weinberg equilibrium ($P < 10^{-6}$); 5) pruning SNPs for linkage disequilibrium (LD) with a LD r^2 threshold of 0.6 and window size of 50 kb. Finally, 5,995 genetic variants were retained after genotype quality control.

The sample quality control was performed with the following steps: 1) removal of non-British samples (Data-Field 21000); 2) removal of samples with poor heterozygosity or missingness (Data-Field 22010); 3) removal of genetic relatedness by randomly excluding individuals in a pair of samples who are estimated to be genetically related (Data-Field 22011); 4) removal of samples whose genetic sex is inconsistent with report sex (Data-Field 31/22001); 5) removal of samples who has imaging data. Finally, after the first five steps of sample quality control, 488,377 samples were left. To alleviate the computational burden, we randomly selected 10,000 individuals from the total cohort as a representative sample through seed '2022' set by R. For the derived genotype matrix with 5,995 variants and 10,000 samples, the simulation strategy to generate exposures and the outcome is the same as described in **Methods**.

Supplementary simulation with the presence of pleiotropy

For this supplementary simulation, we regenerated the outcome with the impact of direct pleiotropy from variants as follows by taking the prior study as a reference [1]:

$$\boldsymbol{Y} = \boldsymbol{G\tau} + \sum_{i=1}^{K} \beta_{x_i y} \boldsymbol{X}_i + \sum_{i=1}^{K} \delta_{u_i y} \boldsymbol{U}_i + \boldsymbol{\varepsilon}_y$$

where $\boldsymbol{\tau}$ denotes the m-vector of SNP direct effects on the outcome $\boldsymbol{Y}$, which was generated from normal distribution $N(\mu_\tau, {\sigma_\tau}^2)$ with $\mu_\tau = 0.2$ and $\sigma_\tau = 0.05$. $\boldsymbol{Y}$ was then scaled to have a zero mean and unit variance. The remaining notations are consistent with their meanings in the main text. The genotype matrix, exposure matrix, confounding factors matrix, and the effect of exposures and confounding factors remained the same as in baseline simulation. Therefore, direct comparison can be made between each replication in our baseline simulation with no pleiotropy exists, and each replication in this supplementary simulation with pleiotropy exists. For simplicity, we narrowed the comparison among the most focused methods: MR-PL, Multi-2SLS and Uni-2SLS.

Calculation of the conditional F-statistic

Usually, the Sanderson-Windmeijer conditional F-statistic (F_{sw}) is used to tested for the weak instruments problem in one-sample multivariable MR, and a F_{sw} greater than the rule-of-thumb value of 10 means there is sufficient conditional instrument strength [2-4]. The calculation steps of F_{sw} can be briefed as follows:

Step 1. Each exposure $\boldsymbol{X}_i$ ($1 \leqslant i \leqslant K$) is regressed on the whole set of genotype matrix $\boldsymbol{G}$ through the ordinary least square method (OLS), and the predicted value of each exposure $\hat{\boldsymbol{X}}_i$ ($1 \leqslant i \leqslant K$) is calculated;

Step 2. Each exposure $\boldsymbol{X}_i$ is regressed on all the other predicted exposures through OLS to yield the residual term $\boldsymbol{\varepsilon}_i = \boldsymbol{X}_i - \delta_1 \hat{\boldsymbol{X}}_1 - \ldots - \delta_k \hat{\boldsymbol{X}}_{k(k \neq i)} - \ldots - \delta_K \hat{\boldsymbol{X}}_K$;

Step 3. The residual term corresponding to each exposure is then regressed on the whole set of genotype matrix $\boldsymbol{G}$ through OLS, and the conditional F-statistic F_{sw} is obtained as the freedom adjusted F-statistic (F) in this regression as $F_{sw} = F \times M/(M\text{-}K+1)$, where M denotes the total number of instrumental SNPs.

Reference

1. Knutson, K.A., Deng, Y., Pan, W. Implicating causal brain imaging endophenotypes in Alzheimer's disease using multivariable IWAS and GWAS summary data. Neuroimage. 2020;223:117347.
2. Sanderson E, Spiller W, Bowden J. Testing and correcting for weak and pleiotropic instruments in two-sample multivariable Mendelian randomization. Statistics in medicine. 2021;40(25):5434-52.
3. Sanderson E, Windmeijer F. A weak instrument [Formula: see text]-test in linear IV models with multiple endogenous variables. J Econom. 2016;190(2):212-21.
4. Sanderson E, Davey Smith G, Windmeijer F, Bowden J. An examination of multivariable Mendelian randomization in the single-sample and two-sample summary data settings. International journal of epidemiology. 2019;48(3):713-27.
