# Supplementary material for: An augmented Mendelian randomization approach provides causality of brain imaging features on complex traits in a single biobank-scale dataset
Source: PLoS Genet. 2023 Dec 27;19(12):e1011112. doi: 10.1371/journal.pgen.1011112 (PMC10775988; doi:10.1371/journal.pgen.1011112)
Supplement: Attachment — Submitted filename: Response to Reviewers.docx [file pgen.1011112.s052.docx]

Institute of Science and Technology for Brain-Inspired Intelligence

Fudan University

Shanghai 200433, China

September 24, 2023

Dear Editor,

Thank you for your feedback on our manuscript entitled “**An augmented Mendelian randomization approach provides causality of brain imaging features on complex traits in a single biobank-scale dataset**” (PGENETICS-D-23-00635). We sincerely thank all reviewers for their constructive and valuable feedback that we have used to improve the quality of our manuscript.

The reviewers’ comments have been numbered and laid out in italicized font, while our point-to-point response in normal font. Notably, any changes or modifications in the main text have been given in red.

We are hopeful that the revised manuscript would be regarded as appropriate for PLOS Genetics publication. Thank you again for your consideration.

Best regards,

**Dr. Xing-Ming Zhao**

Professor

Institute of Science and Technology for Brain-Inspired Intelligence

Fudan University

Shanghai, P.R. China

Tel. 86-21-55665546

Email: xmzhao@fudan.edu.cn

*Response Letter to*

**An augmented Mendelian randomization approach provides causality of brain imaging features on complex traits in a single biobank-scale dataset**

*Reviewer #1: This is a useful look into partial least squares as a method in the MVMR framework, in cases of extreme upscaling of the number of exposures. Overall, I appreciate the motivation and the applied results and I would recommend that the methods and the simulation study be enhanced and contextualized in related MR literature.*

**REF1 – Response to REF1:** We sincerely thank you for these constructive, valuable and professional comments, which have greatly benefited us. The detailed point-by-point responses are listed below.

**REF1 – Point to point response to REF1**

**REF 1.1 – Issue of conditional instrument strength**

| *Referee Comment* | *Introduction*  *Thinking of the MR assumptions, the central issue at performing a multivariable MR without any further methodological refining is* *the issue of conditionally weak instruments (10.1002/sim.9133). As you correctly point out, a genetic variant is theoretically affecting many brain regions/densities rather than only a single one, and the result of this in the MVMR framework would be most pronounced by* *low conditional instrument strength. Overall, I think the simulations studies could track this explicitly as there is now.* |
| --- | --- |
| Author Response | Thank you for this constructive suggestion. In our data generation process, 250 of the 5,000 total SNPs (i.e., probability of 0.05) were independently and randomly selected as the causal SNPs for each of the 20 exposures. Such data generating mechanism could naturally induce 0%~18.2% overlapping percentage of the causal/instrumental SNPs between any two exposures (**S23 Table**), with a higher heritability setting leading to lower overlapping percentage. For testing the weak instruments problem in one-sample multivariable MR, the Sanderson-Windmeijer conditional F-statistic (*F_sw_*) is often used, and a *F_sw_* value greater than the rule-of-thumb value of 10 means that there is sufficient conditional instrument strength^1-3^. However, in our study, this canonical conditional F-statistic for testing the instrument strength in one-sample multivariable MR may not be applicable to our method, for which the reasons are shown as follows:  (1) The theory of conditional weak instrument F-test is basically built within the framework of 2SLS^2^, where exposures are fitted in a totally different way with our method. In 2SLS, each exposure is predicted separately and parallelly in the first stage, not considering the relationships with other exposures. In this event, when exposures are highly correlated with each other and instruments cannot be strongly associated with each exposure given other exposures, it could cause the issue of multicollinearity in predicted exposures and bias the causal estimates extremely (i.e., conditionally weak instruments bias). The conditional F-statistic is then designed to test this situation, which evaluates the overall strength of instruments on each individual exposure while controlling for other exposures^2^. In comparison, the PLS regression (and Lasso regression) used in our method is specially designed for alleviating the multicollinearity problems in predictor variables and response variables. Unlike 2SLS, PLS fits the regression model iteratively, where all exposures have already been considered as whole in each iterative step. In this context, each exposure is predicted while incorporating information from other exposures, effectively mitigating the impact of other highly correlated exposures, thus through the manner of ‘regressing out’ other exposures to test week instruments might not be suitable.  (2) The *F_sw_* value can hardly reach the rule-of-thumb value of 10 in high-dimension and high-correlation settings. We calculated the *F_sw_* statistics in our simulation. It shown that even in our ideal simulation which covers a board range of scenarios (**S23 Table**), the *F_sw_* value was far from reaching 10 (with a mean of 4.37 before WC-correction, and 5.63 after WC-correction, **S23A Fig** and **S6 Table**). To be more specific, the *F_sw_* value in any replication of any parameter setting cannot reach 10 in our simulation, not to mention in real data applications (all *F_sw_* values were below 2 in our real data application, **S11 Table**).  (3) The *F_sw_* value is highly sensitive to the number of instruments and exposures, limiting its applicability in high-dimension scenarios. Since a larger parameter *c* value in our WC-correction procedure will induce less IVs and exposures, we altered the *c* values from 0 to 40 (step by 5) and calculated the corresponding *F_sw_* values (took the mean of 20 exposures) for all 43,200 simulations (100 replications × 48 settings × 9 *c* values). The results revealed a strong negatively correlation between *F_sw_* and both the number of exposures (*r* = -0.4, *P* < 5×10^-12^, Spearman correlation, **S23B Fig**), and the number of instruments (*r* = -0.21, *P* < 5×10^-12^, Spearman correlation, **S23C Fig**). Notably, we found that only when the number of instruments is fewer than 31 and the number of exposures is fewer than 12 can the *F_sw_* value entirely surpassed 10. When the number of exposures and instruments increased, the *F_sw_* value experienced a notable decline.  (4) Forcing to make the conditional F statistics larger than the rule-of-thumb value of 10 could lead to poor results of causal estimates. We segmented the *F_sw_* values into distinct bins for all the 43,200 simulations (100 replications × 48 settings × 9 *c* values), and investigated the performance of MR-PL within each bin. It shown that a higher *F_sw_* value is not consistently linked with improved performance of causal estimation, and simulations falling within the bin with *F_sw_* values excessed 10 gave the poorest performance in MSE and type I error rate control (**S23F-G Fig**). This is possibly because a higher *F_sw_* value indicates a reduced number of instruments and exposures (**S23B-E Fig**), therefore limiting the statistical capacity of MR in estimating causal effects, which is consistent with previous studies^4-9^.  In summary, the issue of weak instrument strength is too complicated and challenging in high dimension and high correlation scenarios. Actually, few studies have examined this issue in the cases of extreme upscaling the numbers of exposures and instruments. Although the current conditional F-statistic as well as its rule-of-thumb value for detecting weak instruments have some limitations, we acknowledge that our method cannot deal with the issue of detecting weak instruments in one-sample, high-dimension and high-correlation situations. Incorporating dimensionality reduction techniques in MR such as PCA-based MR approaches might be one of the smart ways to solve this problem^10^. We believe that more robust and data-driven statistical tools to evaluate the instrument strength and post-MR refining methodologies would be developed in cases of extreme upscaling the numbers of exposures and instruments in the near future. In the revised manuscript, we have followed the reviewer’s suggestion to explicit this issue in simulations and discussion. We thank the reviewer again for this valuable suggestion.    **S23 Fig. Results of conditional F-statistic values (*F_sw_*). (A)** Boxplot of the conditional F-statistics before and after WC-correction, with a mean of 4.37 before WC-correction, and 5.63 after WC-correction. **(B, D)** Scatter plots colored by density of the conditional F-statistics and exposure numbers across 43,200 simulations (100 replications × 48 settings × 9 *c* values), where **(D)** displays separate plots for different parameter settings of heritability. **(C, E)** Scatter plots colored by density of conditional F-statistics and instrumental SNP numbers across 43,200 simulations (100 replications × 48 settings × 9 *c* values), where **(E)** displays separate plots for different parameter settings of heritability. **(F)** A comparison of Mean squared error (MSE) for MR-PL across different bins of conditional F-statistic values. The error bar represents the variance of MSE. **(E)** A comparison of type I error rate for MR-PL across different bins of conditional F-statistic values. The error bar represents the variance of type I error rate. The red dotted line denotes the rule-of-thumb value of 10. *r*: the estimate of Spearman correlation; *P*: the *P*-value of Spearman correlation test; n_neighbors: the number of dots around each dot. |
| Excerpt 1.1 | **Lines 297-307 (Main text):**  Additionally, we examined the Sanderson-Windmeijer conditional F-statistics (*F_sw_*) values in our simulations [18, 27] (see **S1 Text** for more details). It shown that even in our ideal simulation with sufficient exposure-specific instruments, the *F_sw_* values can hardly reach the rule-of-thumb value of 10 (with a mean 4.37 before WC-correction, and 5.63 after WC-correction at *c*=20, **S23A Fig** and **S6 Table**). To investigate the simulation performance under different levels of conditional F-statistics, we segmented the corresponding *F_sw_* values into distinct bins for all the 43,200 simulations (100 replications × 48 settings × 9 *c* values varying from 0 to 40). It was observed that the *F_sw_* values were strongly negatively correlated with the number of instruments and exposures (**S23B-E Fig**). Moreover, simulations falling within the bin with *F_sw_* values excessed 10 gave the poorest performance in MSE and type I error control in our method (**S23F-G Fig**). These indicate the potential limitation of the conditional F-statistic in application of high-dimension and high-correlation situations.  **Lines 523-539 (Main text):**  The issue of weak instruments is often raised in multivariable MR, which is arguably very complex and challenging in high-dimension and high-correlation scenarios. According to our results, the conditional F-statistic *F_sw_* can hardly reach the rule-of-thumb value of 10 neither in our ideal simulations nor real data applications (**S23A Fig**, **S6 and S11 Tables**), and it is highly sensitive to the number of instruments and exposures (**S23B-E Fig**). Forcing to make it larger than 10 by reducing the number of instruments or exposures could result in poor causal estimation results (**S23E-F Fig**). Furthermore, the current conditional F-test used to evaluate weak instrument strength is basically built within the framework of 2SLS, where exposures are fitted in a totally different manner with our method in the first stage (2SLS: separately and parallelly; MR-PL: iteratively and considering all exposures as a whole). These indicate the potential limitation of the conditional F-statistic in evaluating instruments strength under one-sample, high-dimension and high-correlation setting, especially in our method. Currently, few studies have discussed this issue in cases of extreme upscaling the numbers of exposures and instruments, and most of them just constrained the analysis to a limited number of exposures. Incorporating dimensionality reduction techniques in MR or using only exposure-specific IVs as predictors might be one of the smart ways to solve this problem [25, 74, 75]. We believe that more robust and data-driven statistical tools to evaluate the instrument strength and post-MR refining methodologies would be developed in cases of extreme upscaling the numbers of exposures and instruments in the near future.  **Lines 797-800 (Main text):**  The above parameter settings presented the desired numbers of instrumental SNPs ranging from 256 to 1,092, moderate to high correlation between any two exposures ranging from 0.31 to 0.72, and overlapping percentage of causal or instrumental SNPs between any two exposures ranging from 0% to 18.18% (**S23 Table**).  **S1 text:**  **Calculation of the conditional F-statistic**  Usually, the Sanderson-Windmeijer conditional F-statistic (*F_sw_*) is used to tested for the weak instruments problem in one-sample multivariable MR, and a *F_sw_* greater than the rule-of-thumb value of 10 means there is sufficient conditional instrument strength [2-4]. The calculation steps of *F_sw_* can be briefed as follows:  Step 1. Each exposure $X_{i}$ (1≤*i*≤*K*) is regressed on the whole set of genotype matrix $G$, and the predicted value of each exposure $\hat{X}_{i}$ (1≤*i*≤*K*) is calculated;  Step 2. Each exposure $X_{i}$ is regressed on all the other predicted exposures to yield the residual term $\varepsilon_{i}=X_{i}-\delta_{1}\hat{X}_{1}-...-\delta_{k}\hat{X}_{k(k\neq i)}-...-\delta_{K}\hat{X}_{K}$;  Step 3. The residual term corresponding to each exposure is then regressed on the whole set of genotype matrix $G$, and the conditional F-statistic *F_sw_* is obtained as the freedom adjusted F-statistic (*F*) in this regression as *F_sw_*= *F*×*M*/(*M*-*K*+1), where *M* denotes the total number of instrumental SNPs. |

**REF 1.2 – Clarifying the modifiability of the exposures**

| *Referee Comment* | *Introduction*  *While the application to imaging data is interesting and imaging data is a good contender for highly correlated data, there is discussion on MR literature on whether the target of the estimations is a modifiable exposure.* *Brain volumes are not readily modifiable in the adult life.* |
| --- | --- |
| Author Response | Thanks for your suggestion. Both the grey matter (including cortical thickness, volume and area) and white matter have been shown to be highly dynamic throughout the lifespan, and also linked to various disorders and complex traits^11-13^. These observations suggest that there are potential causal relationships between these brain imaging features and complex traits or disorders.  In the revised manuscript, we have added discussion on the relevant MR literatures which explored the causal effects of different imaging modalities, including cerebral cortex and white matter microstructure. We have also explicitly stated that while we primarily focused on white matter microstructure in the real data application, it is still applicable to other high-dimensional modalities such as cerebral cortex imaging features, metabolic profiles, proteomic profiles and microbiomes. |
| Excerpt 1.2 (main text) | **Lines 65-68:**  For instance, a recent study investigated the causal relationships between 587 IDPs (e.g., cortical volume, area, thickness and white matter microstructure) and 10 psychiatric disorders and reported 11 causal pairs (e.g., the causal association of right superior fronto-occipital fasciculus fractional anisotropy and left accumbens volume on schizophrenia) [3].  **Lines 129-132:**  MR-PL is a universal method that is capable of diverting high-dimensional omics data (e.g., transcriptome, proteome, metabolome, and microbiome). Given the prevalence and maturity of biobank-scale datasets for brain imaging compared with other molecular omics datasets, the focus was placed on IDPs that were adopted as the exposures in this study.  **Lines 497-502:**  In this study, we primarily focused on white matter microstructure as exposures in the real data applications. It’s noted that exposures can still be modified as other imaging modalities, considering the emerging causal relationships reported between different imaging modalities (e.g., brain volumes) and complex traits [3, 25]. Furthermore, it’s natural to apply MR-PL to other high-dimensional omics data such as metabolic profiles, proteomic profiles and microbiomes. |

**REF 1.3 – Providing results for bias and variance**

| *Referee Comment* | *Results*  *(1) There is previous work tackling a similar issue with PCA and sparse PCA (10.7554/eLife.80063), as well as applied work on components of body composition (DOI: 10.1038/s42003-021-02550-y). The common thread in these works and your work is an improvement in precision with a less clear result on bias. I would encourage you to present separate simulation results for bias and variance.*  *(2) How did you choose the performance measure of MSE? Granted it is a combination of bias and variance, but I've found it difficult to tell exactly where the benefit of MR-PL lies, in bias or in variance? I imagine it is predominantly due to a reduction in variance of the obtained estimates, similar to two other approaches mentioned above.* |
| --- | --- |
| Author Response | Thank you for this valuable suggestion. We have carefully read these two papers. We chose to use MSE as the performance metric because it is a widely used index for evaluating performance of causal estimation in relevant MR literatures^5; 14; 15^. As you note, MSE can effectively combine the estimate bias and variance, making it a more comprehensive measurement than bias and variance alone. In the revised manuscript, we have provided separate simulation results for bias and variance of causal estimation under each parameter setting (**S1 Fig** and **S3 Table**). It was observed that MR-PL consistently outperformed other approaches in yielding lower values of both bias and variance. According to these results, we can say that the benefit of MR-PL for causal estimation lies in both bias and variance reduction. In the revised manuscript, we have added these results.    **S1 Fig. Bias and variance results of MR-PL and other MR approaches in baseline simulation.** Bias was calculated as the mean of the absolute difference between causal estimate and true effect, and variance was calculated as the variance of causal estimate minus true effect (averaged across 100 replications for each setting). The results from the methods of ImagingMR and PRS-2SLS were not included here. The full results can be found in **S3 Table**.  **S3 Table. Bias and variance results (values within parentheses) of MR-PL and other MR approaches in baseline simulation.** Bias was calculated as the mean of the absolute difference between causal estimate and true effect, and variance was calculated as the variance of causal estimate minus true effect (averaged across 100 replications for each setting). The lowest value is highlighted in bold in each scenario, and the second lowest value is highlighted in underline.   |
| Excerpt 1.3 (main text) | **Lines 195-199:**  Given MSE is a combination of bias and variance, we also provided separate simulation results for bias and variance of causal estimation. It shown that MR-PL typically yielded lower values for both bias and variance compared to other approaches in nearly all settings, followed by PLS-Elasticnet (**S1 Fig** and **S3 Table**). The results indicate that the benefit of MR-PL for causal estimation lies in both bias and variance. |

**REF 1.4 – The alignment between results in real data application and simulation**

| *Referee Comment* | *Results*  *It would be interesting to see the results of the method but the results of a simple two-stage least squares approach, univariable and multivariable, and whether the observed differences in the real data examples line up with the simulation results. I would expect that there will be less strong effects in MVMR given the conditionally weak instrument problems.* |
| --- | --- |
| Author Response | Thanks for your suggestion. We have provided the results of Uni-2SLS and Multi-2SLS in the real data applications in **S20 Table**. As the reviewer expected, more strong effects were obtained in Uni-2SLS (469 pairs with *P*-value < 0.05, 105 pairs with *P*-value < 5×10^-3^, 25 pairs with *P*-value < 5×10^-4^) than Multi-2SLS (440 pairs with *P*-value < 0.05, 72 pairs with *P*-value < 5×10^-3^, 11 pairs with *P*-value < 5×10^-4^). This is in line with the simulation results that Uni-2SLS generally gave more inflated type I error rate than Multi-2SLS (**Fig 3A**). Additionally, we compared the overall causal estimation pattern between our method and these two 2SLS-based approaches. Significant correlation of IDP-trait *β*-maps were observed among the three methods (*r* = 0.33 between MR-PL and Multi-2SLS, *r* = 0.53 between MR-PL and Uni-2SLS, *r* = 0.27 between Multi-2SLS and Uni-2SLS, all with *P* < 5×10^-5^, Spearman correlation). These results suggest that there is a uniform trend in the causal estimations across these methods, which is also in line with the simulation results that all of them exhibited a consistent MSE under varying parameter settings. For example, all the three methods shown lower MSE when the heritability *hg^2^* is higher and the interference strength of confounding factors *α_ux_* is weaker (**Fig 2**). We added some texts in the revised manuscript to report the results from Uni-2SLS and Multi-2SLS. |
| Excerpt 1.4 (main text) | **Lines 432-437:**  Results of Uni-2SLS and Multi-2SLS are also provided in **S20 Table**. More strong causal effects were obtained in Uni-2SLS than Multi-2SLS, in line with the simulation results that Uni-2SLS generally gave more inflated type I error rate than Multi-2SLS (**Fig 3**). A consistent trend in causal estimations was observed across MR-PL, Uni-2SLS and Multi-2SLS (*r* = 0.33 between MR-PL and Multi-2SLS IDP-trait *β*-maps, *r* = 0.53 between MR-PL and Uni-2SLS IDP-trait *β*-maps, *r* = 0.27 between Multi-2SLS and Uni-2SLS IDP-trait *β*-maps, all with *P* < 5×10^-5^, Spearman correlation). |

**REF 1.5 – Interpreting the results**

| *Referee Comment* | *Results*  *The WC-corrected version of the method appears to provide lower MSE. How do you interpret this? In my understanding, a WCC would be targeting erroneously precise estimates that have just crossed the limit of selection. I would think that these SNPs should be still included, despite their increased variance when corrected.* |
| --- | --- |
| Author Response | Thank you for the comment. Based on the newly provided simulation results for bias and variance mentioned in **REF 1.3**, we noticed that our method generally achieved even lower values of bias and variance through the WC-correction procedure (**S1 Fig** and **S3 Table**). Therefore, the improvement in MSE observed in our method after WC-correction can be interpreted as an improvement in both bias and variance reduction. This could be attributed that the GWAS estimates of many non-causal SNPs might be highly upward biased due to substantial confounding factors and thus leading to mistaken IV selection, and WC-correction procedure could further refine the selection boundary and significantly reduce the chances for non-causal SNPs chosen as IVs which would otherwise bias MR results greatly (**R1 Fig**, *P*-value = 1.63×10^-6^, Wilcoxon signed rank test^16^). Given the further enhanced MR results in minimizing MSE, bias, variance, and controlling type I error rate across diverse scenarios after WC-correction, along with the long-raised WC issue by using identical sample for IV selection and MR analysis, we believe it is still needed. Nevertheless, we acknowledge that the MR performance under our WC-correction procedure is not optimal and is dependent on multiple factors, such as the genetic architecture and correlation pattern of exposures. In the future, more accurate WC-correction methods tailored to MR with high dimension and high correlation situations would be developed. In the revised manuscript, we reported the bias and variance results of our method after WC-correction, as well as added the discussion on the limitation of WC-correction.    **R1 Fig.** **Percentage of non-causal SNPs selected as IVs before and after WC-correction.** Each dot represents the percentage of non-causal SNPs selected as IVs under a specific parameter setting (number of non-causal SNPs selected as instruments / total number of non-causal SNPs), averaged across 100 replications. The Wilcoxon signed rank test was used for testing the difference of the percentage before and after WC-correction across all 48 parameter settings. |
| Excerpt 1.5 (main text) | **Lines 275-277:**  As depicted in **Fig 2** and **S2 Table**, MR-PL with WC-correction (*c* =20, not specified below) can further lower MSE across almost all scenarios when compared with the absence of WC-correction. Such reduction also applied to both bias and variance (**S1 Fig** and **S3 Table**).  **Lines 563-568:**  It should be noted that our simple WC-correction is not optimal and is dependent on multiple factors, including the genetic architecture of exposures, correlation pattern among exposures, linkage disequilibrium pattern of instrumental SNPs, and the ancestry of population. In the future, more accurate WC-correction methods tailored to MR with high-dimension and high-correlation situations would be developed. With rapid accumulation of the biobank-scale datasets and GWAS summarized data, the WC bias issue can be entirely avoided by selecting IVs based on a different dataset [7]. |

**REF 1.6 – Discussing the approaches**

| *Referee Comment* | *Methods*  *If I understand correctly, PLS is used and the X-Y covariance-explaining components that are generated are fixed to be equal to the number of exposures. Therefore, it is not primarily used as a dimensionality reduction approach. In PCA approaches, including those described in the above two papers for MR, the target is to generate components that explain the variance of X, and then use those in second-stage causal analyses. Could you discuss this?* |
| --- | --- |
| Author Response | Certainly. Thank you for the comment. We want to discuss these two approaches from two aspects: the algorithm and the interpretability in the MR context.  (1) Algorithm: PLS is specifically designed for regression tasks, rather than dimensionality reduction, as you mentioned. In PLS, latent components of input variables are iteratively created to maximize covariance with the response variables, and the response variables are regressed iteratively with these latent components simultaneously (**Lines 621-659**). PLS integrates the dimensionality reduction process into its iterative regression steps. It is used when we have a predictive modeling problem, such as the first stage of one-sample MR where we want to establish a relationship between the instruments and exposures. In contrast, PCA is specifically designed for dimensionality reduction of input variables. It seeks to find orthogonal principal components that capture the maximum variance of input variables. Notably, it does not consider the response variables during the transformation process.  (2) Interpretability in the MR context: The use of PLS regression in our method does not affect the interpretability of MR, but the PCA-based approaches mentioned above does. The reason is that our method is designed within one-sample MR framework, where the interpretability mainly lies in the second stage. Since PLS regression can predict each individual exposure in the first stage, it will not affect the causal estimation for each exposure in the second stage. Conversely, the PCA-based approaches mentioned above are designed within two-sample MR framework, with only one stage of linking SNP-outcome associations with SNP-exposure associations. Therefore, grouping a set of original exposures into a new synthetic exposure through dimensionality reduction techniques would directly impact the interpretability of MR.  Despite the loss of model interpretability with the use of PCA-related techniques in above-mentioned literatures, we still believe it is a wise way in dealing with the issue of weak instruments. In the revised manuscript, we included the relevant discussion. |
| Excerpt 1.6 (main text) | **Lines 684-697:**  The utilization of PLS regression in our method can be compared with previous MR approaches using techniques related to principal component analysis (PCA) [74, 75]. In terms of algorithm, PLS regression is specifically designed for regression predictive tasks, such as the first stage of one-sample MR where we want to establish a relationship between instrumental and exposure variables. It integrates the dimensionality reduction process into its iterative regression steps between the input and response variables. In contrast, PCA is specifically designed for dimensionality reduction of input variables, while the response variables are not considered during the transformation process. In terms of interpretability, the use of PLS regression in our method does not affect the interpretability of MR, but the PCA-based approaches mentioned above does. The reason is that our method is designed within one-sample MR framework where the interpretability mainly lies in the second stage. Conversely, there is only one stage of linking SNP-outcome associations with SNP-exposure associations in two-sample MR where the PCA-based approaches are designed, thus grouping a set of original exposures into a new synthetic exposure through dimensionality reduction techniques will surely influence the interpretability.  **Lines 535-537:**  Incorporating dimensionality reduction techniques in MR or using only exposure-specific IVs as predictors might be one of the smart ways to solve this problem [25, 74, 75]. |

**REF 1.7 – Explaining the failure of assumption with sufficient conditional instrument strength**

| *Referee Comment* | *Methods*  *In the data generating mechanism for the exposure, it is not clear how exactly the MVMR assumptions would fail. Shouldn't there be a lack of exposure-specific instruments? It's not clear to me how, out of a pool of 5,000 SNPs, of whom a random selection is affecting each exposure, would violate the assumption that there is sufficient conditional instrument strength. That is, I imagine MVMR might be feasible if, of those 5,000 SNPs, some are affecting X_1, some others that are independent are affecting X_2 and so on.* |
| --- | --- |
| Author Response | Thank you for the suggestion. We want to clarify this issue from the calculation steps of conditional F-statistic (i.e., *F_sw_*)^2; 3^, which can be briefed in the one-sample MR setting as follows (also added to illustrate in **S1 Text**):  Step 1. Each exposure $X_{i}$ (1≤*i*≤*K*) is regressed on the whole set of genotype matrix $G$, and the predicted value of each exposure $\hat{X}_{i}$ (1≤*i*≤*K*) is calculated;  Step 2. Each exposure $X_{i}$ is regressed on all the other predicted exposures to yield the residual term$\varepsilon_{i}=X_{i}-\delta_{1}\hat{X}_{1}-...-\delta_{k}\hat{X}_{k(k\neq i)}-...-\delta_{K}\hat{X}_{K}$;  Step 3. The residual term corresponding to each exposure is then regressed on the whole set of genotype matrix $G$, and the conditional F-statistic *F_sw_* is obtained as the freedom adjusted F-statistic in this regression.  From the above steps, it is obvious that what the conditional F-statistic tests is the overall strength of the whole instruments on each exposure given other exposures, rather than the strength of IVs specific to each exposure. As the number of exposures increases, the total number of IVs will also increase, but the number of IVs specific to each exposure remains unchanged. This will induce a lower proportion of exposure-specific IVs to the total number of instruments. Consequently, the strength of IVs specific to each exposure given other exposures will be greatly weakened as the total set of IVs (and exposures) increases, resulting in a low conditional F-statistic. This tendency has been depicted in **S23B-E Fig**, where the conditional F-statistic experienced a notable decline when the number of instrumental SNPs and exposures increased (also mentioned in **REF 1.1**). Therefore, if the conditional instrument strength is evaluated through current conditional F-statistic, the assumption of sufficient conditional instrument strength will surely be violated since it’s nearly impossible for *F_sw_* to reach 10 in cases of extreme upscaling the numbers of the instruments and exposures. |
| Excerpt 1.7 | Not applicable. |

**REF 1.8 – Writing issue**

| *Referee Comment* | *Methods*  *In the data generating mechanism for the outcome, I understand that the exposures that are truly causal (beta_i != 0 in equation 2, line 512) are drawn from a uniform distribution. I would suggest to look into more informative mechanisms, for instance if one of the groups of exposures is affecting Y. For example, what seemed to be a determinant of performance in DOI: 10.7554/eLife.80063 is the number of non-causal exposures in those blocks of exposures that contain some causal ones. That is, if X_1 is causal and correlated with X_2, these two were grouped together and X_2 counted as a false positive. Would this be the case with your approach as well?* |
| --- | --- |
| Author Response | Thank you for the suggestion. We sincerely apologize for the confusion caused by the inconsistent use of notation in our manuscript, which has led to a misunderstanding. Actually, we have already performed the simulations covering this case. In the original manuscript, we used different notations (*β_xy_*, $\beta$ and $\theta_{k}$) for the causal effect of exposure on the outcome, and similar notations (*β_ux_* and $\beta_{u_{k}}$) for the effect of confounding factors at different places, which caused the confusion. In the revised manuscript, we uniformly used $\beta_{xy}$ to represent the causal effect of exposures on the outcome, $\alpha_{ux}$ to represent the effect of confounding factors on exposures, and $\delta_{uy}$ to represent the effect of confounding factors on the outcome throughout the manuscript. In our simulation, the true causal effects of exposures *β_xy_* were randomly drawn from both continuous sets of U(-0.1,0.1), U(-0.2,0.2) or U(-0.3,0.3), and discrete sets of {-0.1,0,0.1}, {-0.2,0,0.2} or {-0.3,0,0.3} (**S1 Table, Lines 791-793**). For example, when *β_xy_* is drawn from the set {-0.2,0,0.2}, it means that each of the 20 simulated exposures has an equal possibility (1/3) of being a true causal one with a moderate positive effect of 0.2, a true causal one with a moderate negative effect of -0.2, or a non-causal one with an effect of 0. Similar interpretations apply to the other discrete sets (strong effect: {-0.3,0,0.3}; weak effect: {-0.1,0,0.1}). Accordingly, this setup induces a group of true causal exposures positively or negatively affecting the outcome, and a group of non-causal exposures, all of which are correlated with each other. We believe this is the case you mentioned. We have checked similar problems of notation usage throughout the manuscript and provided clear explanations for what they referred to. |
| Excerpt 1.8 (main text) | **Lines 778-782:** $\alpha_{u_{k}x}$  and $\delta_{u_{k}y}$ denote the effect size of confounder $u_{k}$ on exposure $x_{k}$ and the outcome *y*, respectively; $U_{k}$ denotes the *N*-vector observations of confounder $u_{k}$ (1≤*k*≤*K*) and was independently drawn from $N(0,(1-hg^{2}-\sigma^{2})/\sum_{i=1,i\neq k}^{K} \alpha_{u_{i}x}^{2})$, leading to correlations among exposures and the outcome; $\beta_{x_{k}y}$ expresses the causal effect size of exposure $x_{k}$ and the outcome *y*.  **Lines 788-793:**  For the effect of confounding factors on exposures $\alpha_{u_{k}x}$ (1≤*k*≤*K*), it was randomly drawn from uniform distribution of either U(0,5) or an increased effect size U(5,10). For the effect of confounding factors on the outcome $\delta_{u_{k}y}$, it was randomly drawn from uniform distribution U(0,1). For the true causal effect of exposures $\beta_{x_{k}y}$ (1≤*k*≤*K*), it was randomly drawn from discrete sets of {-0.1,0,0.1}, {-0.2,0,0.2} or {-0.3,0,0.3}, or continuous set U(-0.1,0.1), U(-0.2,0.2) or U(-0.3,0.3).  **Lines 160-164:**  To evaluate the performance of MR-PL, the individual-level data of independent SNPs, exposures, and the outcome were simulated across a variety of scenarios, covering different exposures heritability *hg^2^* (from low to high), different interference strengths of confounding factors on exposures *α_ux_* (low-power and high-power), and different types of true causal effects *β_xy_* (discrete and continuous; weak, moderate, and strong) (see **S1 Table** for all parameter settings). |

**REF 1.9 – Adding algorithmic details**

| *Referee Comment* | *Methods*  *Seeing that a difference with the mentioned PCA-based methods is the ability to provide inference on each individual exposure (rather than groups of exposures), I believe a more thorough discussion of what exactly the coef() function in the plsr pipeline is achieving. It was not clear how exactly there is a back transformation after the dimensionality reduction which PLS entails.* |
| --- | --- |
| Author Response | Thank you for your suggestion. We have carefully read the official documentation of R package pls, the associated paper^17^, and the mathematical modeling algorithms book^18^. In the revised manuscript, we have included more details of PLS algorithm and its back transformation after the dimensionality reduction. |
| Excerpt 1.9 (main text) | **Lines 621-659:**  We denote *N* as the sample size, *M* as the total number of SNPs selected as IVs, *K* as the total number of exposure variables. We also denote $G=\{G_{1},G_{2},...,G_{M}\}=\{g_{ij}\}_{N\times M}$ as *N* by *M* genotype matrix, where $G_{m}$ (1≤*m*≤*M*) denotes the *N*-vector observations of SNP $g_{m}$, $X=\{X_{1},X_{2},...,X_{K}\}=\{x_{ik}\}$ as *N* by *K* exposure matrix, where $X_{k}$ (1≤*k*≤*K*) denotes the *N*-vector observations of exposure variable $x_{k}$, ***Y*** denotes the *N*-vector observations of the outcome variable *y*. We model the association among the genotypes, exposures and outcome through the following two-stage estimation procedure. $X=GC+E$  (1) $Y=\hat{X}\beta+\varepsilon$  (2)  Where ***C*** denotes the coefficient matrix of ***G*** on ***X***; $\hat{X}$ represents the fitted matrix of *K* exposures from (1) which equals $G\hat{C}$; $\beta$ expresses a *K*-dimensional vector that represents the causal effect of exposures on the outcome; ***E*** is an *N* by *M* matrix of residual error; $\varepsilon$ is an *N*-vector of residual error.  Eq. (1) regresses the exposure matrix with genotype matrix to predict the genetically fitted exposure matrix. In the above step, Partial Least Squares (PLS) regression is adopted to model this association, which accounts for the variation in ***G*** and ***X*** and the correlation between them [22]. Both ***G*** and ***X*** are assumed to be first scaled to have zero mean and unit variance for the respective column, and PLS regression is briefed as follows. First, the first component $\hat{u}_{1}=G\rho^{(1)}$ and $\hat{v}_{1}=X\gamma^{(1)}$can be derived by solving the following conditional extremum problem, where$\rho^{(1)}$ denotes a *M*-vector loadings of SNPs and $\gamma^{(1)}$ is a *K*-vector loadings of exposures.   (3)  The optimization problem can be solved using Lagrange multiplier or SVD decomposition. The solution equals to calculate the eigenvalue and eigenvectors of $G^{T}XX^{T}G$. Suppose , then the maximum eigenvalue of $G^{T}XX^{T}G$ is $\theta^{2}$. The corresponding unit eigenvector of $\theta^{2}$ is the solution $\rho^{(1)}$, and then $\gamma^{(1)}$ is calculated as $\frac{1}{\theta}X^{T}G\rho^{(1)}$. Second, the regression model of ***X*** and ***G*** on $\hat{u}_{1}$ is built, respectively. $\begin{matrix} G=\hat{u}_{1}\sigma^{(1)T}+G_{1}^{res} \\ X=\hat{u}_{1}\tau^{(1)T}+X_{1}^{res} \end{matrix}$  (4)  where $\sigma^{(1)}=G^{T}\hat{u}_{1}/\vert\vert\hat{u}_{1}\vert\vert^{2}$ and $\tau^{(1)}=X^{T}\hat{u}_{1}/\vert\vert\hat{u}_{1}\vert\vert^{2}$ denote the coefficient vector estimated using the ordinary least square method (OLS); $G_{1}^{res}$ and $X_{1}^{res}$ represent the residual matrix. Third, residual matrix $G_{1}^{res}$ and $X_{1}^{res}$ are substituted for ***G*** and ***X***, and the above steps (3)-(4) are repeated. Then we have the following parameter vectors: $\rho^{(2)}$, $\gamma^{(2)}$, $\hat{u}_{2}=G\rho^{(2)}$, $\hat{v}_{2}=X\gamma^{(2)}$, $\sigma^{(2)}=G^{T}\hat{u}_{2}/\vert\vert\hat{u}_{2}\vert\vert^{2}$, $\tau^{(2)}=X^{T}\hat{u}_{2}/\vert\vert\hat{u}_{2}\vert\vert^{2}$. The regression model after the second iteration is built as $\begin{matrix} G=\hat{u}_{1}\sigma^{(1)T}+\hat{u}_{2}\sigma^{(2)T}+G_{2}^{res} \\ X=\hat{u}_{1}\tau^{(1)T}+\hat{u}_{2}\tau^{(2)T}+X_{2}^{res} \end{matrix}$  (5)  *r* repetitions are assumed to be conducted in total, and *r* components $\hat{u}_{1}$, $\hat{u}_{2}$, …,$\hat{u}_{r}$are obtained. Subsequently, the following equations hold. $\begin{matrix} G=\hat{u}_{1}\sigma^{(1)T}+...+\hat{u}_{r}\sigma^{(r)T}+G_{r}^{res} \\ X=\hat{u}_{1}\tau^{(1)T}+...+\hat{u}_{r}\tau^{(r)T}+X_{r}^{res} \end{matrix}$  (6)  The regression coefficient $\hat{C}$ is calculated by taking each score $u_{i}=\rho_{1}^{(i)}g_{1}+...+\rho_{M}^{(i)}g_{M}$ (1≤*i*≤*r*) back into $X=u_{1}\hat{\tau}^{(1)T}+...+u_{r}\hat{\tau}^{(r)T}$ to fit the regression model $\hat{X}=G\hat{C}$. Suppose that after each iteration the vectors $\rho^{(i)}$, $\sigma^{(i)}$, $\tau^{(i)}$ are saved as columns in matrix $P$,$\Sigma$, $T$, then the coefficient matrix $\hat{C}$ can be written in a less intuitive way as $\hat{C}=P(\Sigma^{T}P)^{-1}T^{T}$ [77].  **Lines 661-663:**  The PLS analysis was implemented using the R (version 4.2.1) package pls (version 2.8-1) [77]. The above coefficient matrix $\hat{C}$ and predicted exposure matrix $\hat{X}$ can be obtained through function *coef()* and *predict()*. |

**REF 1.10 – Writing issue**

| *Referee Comment* | *Minor comments*  *Table 1: Is the outcome scaled?* |
| --- | --- |
| Author Response | Yes. All traits that served as the outcome were scaled before MR analysis. We clarified it in Methods. |
| Excerpt 1.10  (main text) | **Lines 897-899:**  The obtained IDP residuals, as well as the instrumental SNPs and outcome traits were standardized to have a mean zero and unit variance, and were finally employed for MR analysis. |

**REF 1.11 – Writing issue**

| *Referee Comment* | *Minor comments*  *line 576: 'minizine' minimizing.* |
| --- | --- |
| Author Response | Thanks for pointing out the error. We have corrected “minizing” as “minimizing” in the revised manuscript (**Line 717**). |
| Excerpt 1.11 | Not applicable. |

**REF 1.12 – Writing issue**

| *Referee Comment* | *Minor comments*  *Fig.2, Table 1: 'ML-PL' MR-PL in legends.* |
| --- | --- |
| Author Response | Thanks for pointing out the error. We have corrected ‘ML-PL’ as ‘MR-PL’, and checked similar writing problems throughout the whole manuscript. |
| Excerpt 1.12 | Not applicable. |

*Reviewer #2: The authors propose a one-sample multivariable Mendelian randomization method called MR-PL. As a 2SLS method, it uses partial least square in the first stage to predict exposures using genome-wide significant SNPs, and then uses Lasso in the second stage to select and estimate potential causal exposures with non-zero coefficients, and incorporates de-sparsified Lasso for statistical inference. A heuristic approach for correcting winner's curse is also proposed. The authors apply the proposed method to infer the causal relationships between 37 white matter tracts and 180 traits using UKB individual-level data, and reveal some putative causal white matter tracts on smoking, blood vascular function-related traits, and eating behaviors. Overall, the manuscript is well-organized and generally straightforward. but I do have several concerns and questions:*

**REF2 – Response to REF2:** We would like to thank you for these insightful comments. Following these suggestions, our manuscript has been improved a lot. Our responses are summarized point-by-point below.

**REF2 – Point to point response to REF2**

**REF 2.1 – Writing issue / Comparing with the other approach**

| *Referee Comment* | *The use of PLS in stage 1 by MR-PL was highlighted in the simulation and methods using OLS in stage 1 such as multi-2sls in general had larger MSE and type-I error. The authors explained in page 8 line 177 that 'PLS can better incorporate the correlation between exposures into the estimation of causal effect.' This wasn't clear to me as PLS was used in stage 1 and how is the correlation among exposures incorporated in the second stage. Furthermore, in the implementation of stage 1 in multi-2sls (and uni-2sls), was the whole matrix G used to predict all exposures (X), so for each exposure,* *some irrelevant/non-significant SNPs were used as the predictors as well? This may lead to bad prediction in stage 1 given a large number of SNPs included. Could the authors try the usual implementation,* *where for each exposure, only the relevant set of IVs are used for prediction and obtain X_hat. It was discussed in a previous multivariable 2sls called MV-IWAS [1] and it'd be better to discuss/compare with in this manuscript. So it seems to me the main advantage of using PLS in stage 1 compared with the current implementation of OLS is that it can serve as a variable selection/dimension reduction purpose when predicting X.*  *Ref:*  *[1] Knutson, K. A., Deng, Y., & Pan, W. (2020). Implicating causal brain imaging endophenotypes in Alzheimer’s disease using multivariable IWAS and GWAS summary data. NeuroImage, 223, 117347.* |
| --- | --- |
| Author Response | Thank you for the helpful suggestion. We apologize for the unclear description in this sentence located on Page 8 Line 177 in the original manuscript. In our method, the PLS regression is only used in the first stage to incorporate correlations among exposures, and was not used in the second stage. In the second stage, exposures serve as explanatory variables, and both Multi-2SLS and our method directly incorporate their correlations through the term $X^{T}X$ in linear/Lasso regression (the OLS estimator of exposures in linear regression equals $\beta=\text{（}X^{T}X\text{）}^{-1}X^{T}Y$, and estimator of exposures in Lasso regression can be written as $\beta=\text{（}X^{T}X+\lambda W^{-}\text{）}^{-1}X^{T}Y$, where  and $W^{-}$ denotes a generalized inverse^19^). In the revised manuscript, we have revised the sentence in a more rigorous way: “The next optimal approaches are PLS-Elasticnet, PLS-Ridge and PLS-Lars, indicating that the use of PLS regression in the first stage can better incorporate the correlation between exposures, potentially leading to improved causal effect estimation in the second stage.” (**Lines 181-183**)  We have carefully read the paper of MV-IWAS model. In our initial manuscript, only the significant SNPs associated with each exposure were used as IVs in Uni-2SLS, while all significant SNPs associated with at least one exposure were used as IVs to predict each individual exposure in Multi-2SLS. Therefore, we followed the reviewer’s suggestion, adding the implementation of MV-IWAS where only the relevant IVs of each exposure were used for its prediction in the first stage. In a systematic comparison, we still found that our method generally outperformed all the other methods including MV-IWAS, exhibiting lower MSE and better performance to control type I error rate (**Figs 2 and 3A, S2 Fig, S2 and S4 Tables**). Noticeably, MV-IWAS outperformed Uni-2SLS in MSE across all parameter settings, and outperformed Multi-2SLS in MSE across the majority of parameter settings (**Fig 2** and **S4 Table**). This indicates that only using IVs relevant to each exposure could improve the causal estimation as you said. Overall, we consider that the main advantage of using PLS in our method, compared with 2SLS-related methods, lies in its capacity to effectively address correlations among exposures during the prediction process of stage I, which might lead to enhanced causal effect estimation in stage II. However, we still agree with you that employing a large number of SNPs might lead to suboptimal causal estimation due to issues such as weak instrument bias. In the revised manuscript, we have reported the above results, cited the MV-IWAS paper, and added the necessary discussion.    **Fig 2. Mean squared error (MSE) of MR-PL and other MR approaches.** The parameters were set to cover different exposures heritability *hg^2^*, different interference strength of confounding factors *α_ux_* and different types of causal effects *β_xy_* (**S1 Table**). Under each parameter setting, MSE was averaged over 100 replications. The error bar represents ten-fold variance of the MSE over 100 replications. For settings with too small variance, the error bar tends to degenerate to a point. Notably, the results from the methods of ImagingMR and PRS-2SLS were not included here because the MSE values from the two methods were extremely large to show here. The full results can be found in **S2 Table**.    **Fig 3A. A comparison of type I error rate across MR-PL and other MR approaches.** Simulation settings were included if the causal effect was drawn from the discrete set. MR methods were included if individual *P*-value of causal effect estimate of the respective exposure can be calculated. A method failed to control type I error if its type I error rate exceeded the nominal significance level of 0.05 (red dotted line). The error bar represents twice variance in type I error rate over 100 replications. For settings with too small variance, the error bar tends to degenerate to a point.    **S2 Fig. Type I error rate of MR-PL and other MR approaches in supplementary simulation with the presence of linkage disequilibrium.** Simulation settings were included if the causal effect was drawn from the discrete set. MR approaches were included if individual *P*-value of causal effect estimate of each exposure can be calculated. A method fails to control type I error if its type I error rate exceeds the nominal significance level of 0.05 (red dotted line). The error bar represents the variance of type I error rate over 100 replications. For settings with too small variance, the error bar tends to degenerate to a point. |
| Excerpt 2.1  (main text) | **Lines 167-170:**  MR-PL was compared with eight one-sample approaches including: (1) 2SLS-based methods: multivariable 2SLS (Multi-2SLS), univariable 2SLS (Uni-2SLS), polygenic risk score-based 2SLS (PRS-2SLS), and multivariable imaging wide association study (MV-IWAS) [25]; …  **Lines 181-183:**  The next optimal approaches are PLS-Elasticnet, PLS-Ridge and PLS-Lars, indicating that the use of PLS regression in the first stage can better incorporate the correlation between exposures, potentially leading to improved causal effect estimation in the second stage.  **Lines 189-191:**  MV-IWAS exhibited consistently enhanced MSE performance compared to Multi-2SLS and Uni-2SLS (**Fig 2** and **S2 Table**), suggesting that leveraging only exposure-specific IVs for each exposure prediction in multivariable MR might further enhance causal estimation.  **Lines 516-519:**  On the other hand, incorporating larger number of SNPs can elevate the risk of bias in MR estimations due to the weak instrument bias or the potential for some SNPs to have direct effects on the outcome (pleiotropy) since some irrelevant SNPs were used as the predictors as well.  **Lines 535-537:**  Incorporating dimensionality reduction techniques in MR or using only exposure-specific IVs as predictors might be one of the smart ways to solve this problem [25, 74, 75].  **Lines 841-844:**  MV-IWAS is a comprehensive multivariable 2SLS-based MR method which only used SNPs specific to each exposure as IVs [25]. Therefore, we tried this implementation in our one-sample simulation, only using the exposure-specific IVs for the prediction of each exposure in the first stage of 2SLS. |

**REF 2.2 – Refining the part of winner’s curse correction**

*The correction for winner's curse seems to be very heuristic and the current presentation is not convincing enough to me. Though the simulation showed improvement for estimation/MSE after WC correction, this may simply as the result of using more strong IVs and less weak IVs in the ideal scenario with no invalid IVs*

**REF 2.2.1**

| *Referee Comment* | *Could the authors clarify page 31 lines 665-666 'Accordingly, it is expected that the sacrifice of instruments will lead to a larger reduction of MSE.'* |
| --- | --- |
| Author Response | We are sorry for the unclear description which has confused you. It has been shown that using too few SNPs could limit the statistical capacity to detect causality^20^, whereas incorporating more SNPs could help to explain a greater proportion of the variance in exposure^6-9^. As a higher *c* in our WC-correction leads to fewer instruments, our original intent was to say that we hoped the WC-correction procedure can lead to larger reduction in MSE while retaining more instruments. Given the suggestion/discussion in **REF 2.1** that incorporating more SNPs could also increase the potential for some irrelevant/non-significant SNPs included as predictors, we directly used ΔMSE (percentage decrease in MSE) as the index for the selection of *c* in the revised manuscript, which is more intuitive, objective and less heuristic. It shown that the best parameter selection for *c* still remained unchanged at 20, inducing the largest percentage decrease in MSE of 8.03% (**Fig 3B**). This indicates the robustness of the selection. We have revised relevant texts and figures throughout the manuscript.    **Fig 3B. The ΔMSE index at different tuning parameter *c* ranging from 5 to 40 (step by 5) in WC-correction.** ΔMSE denotes the percentage decrease in MSE from MR analysis without WC-correction to MR analysis with WC-correction, averaged across all 48 parameter settings. With the increase of ΔMSE, the greater the MSE was decreased after WC-correction. The Wilcoxon signed rank test^16^ was used to test the difference of ΔMSE between *c*=20 and its neighboring selections of 15 and 25 across all 48 parameter settings. |
| Excerpt 2.2.1 (main text) | **Lines 268-272:**  The index ΔMSE was used for the choice of optimal *c*, where ΔMSE denotes the percentage decrease in MSE from MR analysis without WC-correction to MR analysis with WC-correction. In simulation, *c* varied from 5 to 40 (step by 5), and the value with the highest average ΔMSE across all parameter settings at *P*-value threshold 5×10^-8^ was selected.  **Lines 274-275:**  When *c* was set to 20, MR-PL exhibited the most significant percentage decrease in MSE of 8.03% (**Fig 3B**).  **Lines 808-811:**  For the choice of *c*, the index ΔMSE was defined, where ΔMSE denotes the percentage decrease in MSE from MR analysis without WC-correction to MR analysis with WC-correction. The index ΔMSE was then averaged across all 48 parameter settings, and the optimal *c* was selected with the highest ΔMSE. |

**REF 2.2.2**

| *Referee Comment* | *For the selection of c, is the intuition behind the highest index (decrease in MSE)/(decrease in IV) that MSE is reduced more (so the nominator is larger) and the less IVs are removed (so the denominator is smaller)?* |
| --- | --- |
| Author Response | Yes. The corresponding answer and revision have already been mentioned in **REF 2.2.1** above. |
| Excerpt 2.2.2 | Not applicable. |

**REF 2.2.3**

| *Referee Comment* | *Furthermore, the parameter c is determined based on the simulation studies and fixed to be 20 in their real data analysis. This seemed somewhat arbitrary to me since c=20 was selected based on the *average* across all simulation scenarios, which may not be representative of the real data. More importantly, how would c be selected in other real data analysis for users? The authors should give detailed recommendation on this instead of simply fixing them to 20 as it is affected by sample size, p-value threshold based on their Eq.(10).* |
| --- | --- |
| Author Response | Thank you for the constructive suggestion, and we appreciate this suggestion. We have made the following efforts to further enhance this part.  (1) To make the index for the selection of *c* more objective, intuitive, and less heuristic, we directly used ΔMSE instead of ΔMSE/ΔIV as the index for the choice of *c* (also mentioned in **REF 2.2.1**). The general pattern of index values is still consistent, with the optimal selection of *c* being 20, followed by 15 or 25 (**Fig 3B**). Moreover, the Wilcoxon signed rank test^16^ revealed that the index value of ΔMSE at *c*=20 was significantly higher than that at *c*=15 and 25 across all 48 parameter settings (*P*=2.7×10^-5^ for *c*=15, and *P*=0.036 for *c*=25, **Fig 3B**). These results indicate the selection of *c* being 20 is robust.  (2) In the original manuscript, we have provided the simulation results under each individual parameter setting of our method after WC-correction at *c*=20, which generally enhanced the performance of our method in MSE and type I error control across diverse scenarios including different heritability, confounding factors strength, patterns of causal effects, correlating structure of instrumental variants and *P*-value thresholds for IV selection. To make the selection of *c* more convincing, we added to provide simulation results at neighboring selections of *c* at 15 and 25 under each individual parameter setting (**S9-S12 Figs**). It shown that the implementation of WC-correction at *c*=15 and 25 still led to the performance improvement of MR-PL as WC-correction at *c*=20, generally further reducing MSE and better controlling type I error rate (**S9-S12 Figs**). Such improvement of WC-correction at *c*=15 and 25 still held for different *P*-value thresholds for instrument selection including 5×10^-4^, 5×10^-5^, 5×10^-6^, 5×10^-7^, 5×10^-9^ and 5×10^-10^ (**S17-S20 Figs**). Therefore, it indicates that performance enhancement achieved via WC-correction procedure is not specific to a single choice of *c*, but is robust across a gird of neighboring values.    **S9 Fig.** **A comparison of mean squared error (MSE) between MR-PL with and without winner’s curse correction (WC-correction) at *c*=15 in baseline simulation.** The error bar represents ten-fold variance in MSE over 100 replications in each parameter setting. For settings with too small variance, the error bar tends to degenerate to a point.    **S10 Fig.** **A comparison of mean squared error (MSE) between MR-PL with and without winner’s curse correction (WC-correction) at *c*=25 in baseline simulation.** The error bar represents ten-fold variance in MSE over 100 replications in each parameter setting. For settings with too small variance, the error bar tends to degenerate to a point.    **S11 Fig.** **A comparison of type I error rate between MR-PL with and without winner’s curse correction (WC-correction) at *c*=15 in baseline simulation.** Simulation settings were included if the causal effect was drawn from the discrete set. The error bar represents the variance of type I error rate across 100 simulations for each parameter setting. For settings with too small variance, the error bar tends to degenerate to a point.    **S12 Fig.** **A comparison of type I error rate between MR-PL with and without winner’s curse correction (WC-correction) at *c*=25 in baseline simulation.** Simulation settings were included if the causal effect was drawn from the discrete set. The error bar represents the variance of type I error rate across 100 simulations for each parameter setting. For settings with too small variance, the error bar tends to degenerate to a point.    **S17 Fig.** **A comparison of mean squared error (MSE) between MR-PL with and without winner’s curse correction (WC-correction) at *c*=15 across different *P*-value thresholds to select instrumental variants in baseline simulation.** The error bar represents ten-fold variance in MSE over 100 replications in each parameter setting. For settings with too small variance, the error bar tends to degenerate to a point.    **S18 Fig.** **A comparison of mean squared error (MSE) between MR-PL with and without winner’s curse correction (WC-correction) at *c*=25 across different *P*-value thresholds to select instrumental variants in baseline simulation.** The error bar represents ten-fold variance in MSE over 100 replications in each parameter setting. For settings with too small variance, the error bar tends to degenerate to a point.    **S19 Fig.** **A comparison of type I error rate between MR-PL with and without winner’s curse correction (WC-correction) at *c*=15 across different *P*-value thresholds to select instrumental variants in baseline simulation.** Simulation settings were included if the causal effect was drawn from the discrete set. The error bar represents the variance of type I error rate across 100 simulations for each parameter setting. For settings with too small variance, the error bar tends to degenerate to a point.    **S20 Fig.** **A comparison of type I error rate between MR-PL with and without winner’s curse correction (WC-correction) at *c*=25 across different *P*-value thresholds to select instrumental variants in baseline simulation.** Simulation settings were included if the causal effect was drawn from the discrete set. The error bar represents the variance of type I error rate across 100 simulations for each parameter setting. For settings with too small variance, the error bar tends to degenerate to a point.  (3) We added to provide real data application results in the UKBB with WC-correction of neighboring selections of *c* at 15 and 25 (**S17 and S18 Tables**). High positive correlation of IDP-trait *β*-maps between the results of *c* at 20 and 15/25 were observed (*r* = 0.831/0.851, all with *P* < 5×10^-12^, Spearman correlation), and the associations between IDPs and smoking, blood cell composition and eating behaviors still remained nominal significant (*β* ≠ 0 and *P* < 0.05), implying that MR results with WC-correction in the real data application are robust to the selection of *c* around 20.  (4) We added texts to illustrate that the application of the simple WC-correction procedure won’t change the overall tendency of MR results theoretically **(Lines 546-551)**. For example, in simulations, both MR-PL with and without WC-correction gave low MSE under 0.0034 at parameter setting *hg^2^* = 0.5, *β_xy_*∈{-0.3,0,0.3}, *α_ux_* ∈U(0,5); and both gave large MSE excess 0.023 at parameter setting *hg^2^* = 0.3, *β_xy_*∈{-0.1,0,0.1}, *α_ux_* ∈U(5,10) (**Fig 2**). In the real data application, significant positive correlation of IDP-trait *β*-maps between MR-PL results with and without WC-correction were observed (*r* = 0.792, *P* < 5×10^-12^, Spearman correlation).  (5) We have followed the reviewer’s suggestion to provide detailed recommendation for the choice of *c* in real data applications, where the true causal effect is unknown. We discovered that there was a significant negative correlation between the prediction *R^2^* of the outcome and the reduction in MSE for causal estimation when applying WC-correction (*r* = -0.56, *P* < 5×10^-16^, Spearman correlation, testing for 384 sample points = 48 settings × 8 *c* values ranging from 5 to 40, averaged across 100 replications, **S25 Fig**). This could because a higher prediction *R^2^* of the outcome indicates an overfitting in this context, thus leading to less accurate causal estimations through WC-correction. Therefore, in the revised manuscript, we recommended the selection for *c* as follows (**Lines 555-562**):  Step 1. Determine whether the WC-correction is required; if the IV selection is based on a different sample from that used for MR analysis, then the correction is not required;  Step 2. Find the grid search range for the parameter *c*. Here, we strongly recommend the narrowed grid search range from 15 to 25, which has displayed robust results in both simulation and real data application and shown the best performance in our simulation across diverse scenarios.  Step 3. Select *c* with the lowest prediction *R^2^* of outcome in MR from the grid search range.  Step 4. Examine the robustness of MR results at neighboring values of the selected *c*.    **S25 Fig. Scatter plots colored by density of reduction in Mean squared error (MSE) for causal estimation and prediction *R^2^* of the outcome when applying winner’s curse correction (WC-correction) across 384 simulation** **scenarios (48 settings × 8 *c* values ranging from 5 to 40, averaged across 100 replications).** The reduction in MSE was calculated as the MSE of MR analysis without WC-correction minus the MSE of MR analysis with WC-correction. n_neighbors: the number of dots around each dot.  (6) We acknowledge that even following the above recommendation, the MR performance under our simple WC-correction is not optimal and is dependent on multiple factors, including the genetic architecture of exposures, correlation pattern among exposures, and the ancestry of population. Nonetheless, the simple WC-correction procedure presented here only requires the GWAS *P*-value of SNPs and has shown generally improved MR results across a variety of scenarios including different heritability, confounding factors strength, patterns of causal effects, correlating structure of instrumental SNPs and *P*-value thresholds for IV selection, thereby it can be fast and conveniently applied without adding any additional computational complexity. In the future, more accurate WC-correction methods tailored to MR with high dimension and high correlation situations can be developed. With rapid accumulation of the biobank-scale datasets and GWAS summarized data, the WC bias issue could be entirely avoided by selecting IVs based on a different dataset^21^. In the revised manuscript, we added discussion on the limitation of our WC-correction (**Lines 563-568**). |
| Excerpt 2.2.3 (main text) | **Lines 281-284:**  Similar results were reported for neighboring selections of *c* at 15 and 25 (**S9-S14 Figs**), indicating that performance improvement achieved via WC-correction procedure is not specific to a single choice of *c*, but is robust across a gird of neighboring values.  **Lines 292-294:**  The above conclusion that WC-correction can improve the performance of MR-PL in reducing MSE and type I error rate across various *P*-value thresholds for IV selection still held for neighboring selections of *c* at 15 and 25 (**S17-S20 Figs**), …  **Lines 428-432:**  Analogously, significant positive correlation of *β*-maps were observed between the above main MR-PL results with WC-correction at *c*=20 and MR-PL results with WC-correction at neighboring selections of *c*=15/25 (*r* = 0.831/0.851, both with *P* < 5×10^-12^, Spearman correlation, **S17 and S18 Tables**), as well as MR-PL results without WC-correction (*r* = 0.792, *P* < 5×10^-12^, Spearman correlation, **S19 Table**).  **Lines 541-551:**  The WC-correction procedure presented here only requires the GWAS *P*-values of SNPs and has shown generally improved MR results across diverse scenarios including different heritability, confounding factors strength, patterns of causal effects, correlating structure of instrumental SNPs and *P*-value thresholds for IV selection, as well as robust results in both simulation and real data application. Therefore, it can be fast and conveniently applied without adding any additional computational complexity. Theoretically, the WC-correction procedure won’t change the overall tendency of MR results. For example, in simulations, both MR-PL with and without WC-correction gave low MSE under 0.0034 at parameter setting *hg^2^* = 0.5, *β_xy_*∈{-0.3,0,0.3}, *α_ux_* ∈U(0,5); and both gave large MSE excess 0.023 at parameter setting *hg^2^* = 0.3, *β_xy_*∈{-0.1,0,0.1}, *α_ux_* ∈U(5,10) (**Fig 2**). In the real data application, high causal estimations correlation between MR-PL results with and without WC-correction were observed.  **Lines 551-562:**  Moreover, we discovered that there was a significant negative correlation between the prediction *R^2^* of the outcome and the reduction in MSE for causal estimation when applying WC-correction (*r* = -0.56, *p* < 5×10^-16^, Spearman correlation, testing for 384 sample points = 48 settings × 8 *c* values ranging from 5 to 40, **S25 Fig**). This could because a higher *R^2^* indicates an overfitting in this context, thus leading to less accurate causal estimations. Therefore, we recommend the following selection of *c* in the real data application:  Step 1. Determine whether the WC-correction is required; if the IV selection is based on a different sample from that used for MR analysis, then the correction is not required;  Step 2. Find the grid search range for the parameter *c*. Here, we strongly recommend the narrowed grid search range from 15 to 25 based on our results;  Step 3. Select *c* with the lowest *R^2^* of the outcome in MR from the grid search range;  Step 4: Examine the robustness of MR results at neighboring values of the selected *c*.  **Lines 563-568:**  It should be noted that our simple WC-correction is not optimal and is dependent on multiple factors, including the genetic architecture of exposures, correlation pattern among exposures, linkage disequilibrium pattern of instrumental SNPs, and the ancestry of population. In the future, more accurate WC-correction methods tailored to MR with high-dimension and high-correlation situations would be developed. With rapid accumulation of the biobank-scale datasets and GWAS summarized data, the WC bias issue can be entirely avoided by selecting IVs based on a different dataset [7]. |

**REF 2.2.4**

| *Referee Comment* | *After determining c, in their WC procedure, they directly subtracted a term from the original abs(z-score), then transformed back to the corrected p-value based on the normal distribution. My concern is that after this heuristic transformation does the score still follow the standard normal distribution?* |
| --- | --- |
| Author Response | Thanks for your comment. The purpose of transforming the corrected *z*-score back to the corrected *P*-value in our WC-correction procedure is to use this corrected *P*-value to re-select instrumental SNPs based on the specified threshold (e.g., 5×10^-8^) directly. Moreover, selecting SNPs with corrected *P*-value < threshold is equivalent to selecting SNPs with corrected *z*-score < *Φ*^-1^(1-threshold/2). Such heuristic transformation between *P*-values and statistics, under the assumption of a specific hypothesis being true, is commonly used in similar tools^22; 23^. For example, in the popular gene analysis tool MAGMA, it directly transforms the GWAS *P*-values of SNPs to *χ^2^* statistics^23^. Since the WC-correction procedure can be seen as a secondary instrument selection process (**Lines 492-493**), and neither the corrected *z*-score nor *P*-value itself are used in subsequent MR analysis, we believe their individual value(s) will not impact the estimation of causal effects. |
| Excerpt 2.2.4 | Not applicable. |

**REF 2.2.5**

| *Referee Comment* | *In page 12 line 251-252, 'For type I error control, WC-correction still enhanced the power of MR-PL,' Where is the power result?* |
| --- | --- |
| Author Response | Sorry for the confusion caused by this. In this sentence, we meant to express that WC-correction can enhance the statistical capacity of MR-PL to control type I error rate. In the revised manuscript, we have rephrased this sentence in a more rigorous way as “As shown in **Fig 3A**, the statistical capacity of MR-PL with WC-correction for controlling type I error rate has also been enhanced” (**Lines 277-279**).  Additionally, we have added to provide the power results (represented as 1 - type II error rate at 5% nominal level) in **S8 Fig**. All of the methods were shown to have similar power results, with the exception of PRS-2SLS which had low power under 0.5. The highest power results can be seen for Multi-2SLS, but at a cost of inflated type I error rate and large MSE (**Figs 2 and 3A**). MR-PL displayed overly high-power results ranging from 0.975 to 0.998, highlighting its ability to maintain substantial power while effectively controlling the type I error rate. Application of WC-correction can further increase the power of MR-PL, except for scenarios with low heritability (*hg^2^* = 0.2) where the power slightly reduced. Similar conclusions apply to the neighboring selections of *c* at 15 and 25 (**S13 and S14 Figs**). These results suggest that our method still remains high sensitivity to detect true causal relationships while controlling for type I error rate and minimizing MSE best. In the revised manuscript, we added the relevant results.    **S8 Fig. Power results of MR-PL and other MR approaches in baseline simulation.** Simulation settings were included if the causal effect was drawn from the discrete sets. The error bar represents the variance of power over 100 replications in each parameter setting. For settings with too small variance, the error bar tends to degenerate to a point.    **S13 Fig.** **A comparison of power results between MR-PL with and without winner’s curse correction (WC-correction) at *c*=15 (and other approaches) in baseline simulation.** Simulation settings were included if the causal effect was drawn from the discrete set. The error bar represents the variance of power across 100 simulations for each parameter setting. For settings with too small variance, the error bar tends to degenerate to a point.    **S14 Fig.** **A comparison of power results between MR-PL with and without winner’s curse correction (WC-correction) at *c*=25 (and other approaches) in baseline simulation.** Simulation settings were included if the causal effect was drawn from the discrete set. The error bar represents the variance of power across 100 simulations for each parameter setting. For settings with too small variance, the error bar tends to degenerate to a point |
| Excerpt 2.2.5 (main text) | **Lines 228-235:**  Power results (represented as 1 - type II error rate at 5% nominal level) of approaches were provided in **S8 Fig**. It shown that all the MR approaches had similar power results, except PRS-2SLS which had low power under 0.5 across all settings. The highest power results can be seen for Multi-2SLS, but at a cost of inflated type I error rate and large MSE (**Figs 2 and 3A**). MR-PL displayed overall high-power results ranging from 0.975 to 0.998, highlighting its ability to maintain substantial power while effectively controlling the type I error rate. These results suggest that our method still remains the sensitivity to detect true causal relationships while controlling for type I error rate and minimizing MSE best.  **Lines 281-284:**  Similar results were reported for neighboring selections of *c* at 15 and 25 (**S9-S14 Figs**), indicating that performance improvement achieved via WC-correction procedure is not specific to a single choice of *c*, but is robust across a gird of neighboring values. |

**REF 2.3 – Adding supplementary simulation with the presence of pleiotropy**

| *Referee Comment* | *The method itself cannot deal with pleiotropy which is the main concern in MR, and the Sargan's test is proposed to detect invalid IVs. Could the authors investigate how well the method perform in the presence of pleiotropic IVs and how well is the Sargan's test in this context?* |
| --- | --- |
| Author Response | Thanks for your suggestion. By taking the prior study as a reference^24^, we regenerated the outcome with the impact of direct pleiotropy from variants as follows: $Y= G\tau+\sum_{i=1}^{K} \beta_{x_{i}y}X_{i}+\sum_{i=1}^{K} \delta_{u_{i}y}U_{i}+\varepsilon_{y}$   where $\tau$ denotes the *m*-vector of SNP direct effects on the outcome ***Y***, and was generated from normal distribution $N(\mu_{\tau},\sigma_{\tau}^{2})$ with $\mu_{\tau}=0.2$ and $\sigma_{\tau}=0.05$. ***Y*** was then scaled to have a zero mean and unit variance. The genotype matrix, exposure matrix, confounding factors matrix, and the effect of exposures and confounding factors remained the same as before. Therefore, direct comparison can be made between each replication in our baseline simulation with no pleiotropy exists, and each replication in this supplementary simulation with pleiotropy exists. For simplicity, we narrowed the comparison among the most focused methods: MR-PL, Multi-2SLS and Uni-2SLS. Although MR-PL slightly outperformed the other methods in terms of MSE at most parameter settings (**S5 Table**), all of the methods exhibited a considerably inflated type I error rate larger than 0.2 (**S3 Fig**) when compared to simulations with no pleiotropy exists (**Fig 3A**). These results are to be expected given that none of the methods under comparison can account for pleiotropy itself.  Despite this, the Sargan test used in our method successfully distinguished between scenarios with and without pleiotropy across all parameter settings, which is clearly depicted in the Sargan test *P*-value histograms in **S4-S7 Figs**. Therefore, we are able to recognize and remove out significant MR results that may be attributed to pleiotropy by combining the established pleiotropy-detecting capability of Sargan test with our method. In the revised manuscript, we reported these simulation results, and added to discuss the issue of pleiotropy.  **S5 Table. Mean squared error (MSE) of MR-PL and other MR approaches in supplementary simulation with the presence of pleiotropy.** For each parameter setting, MSE was averaged over 100 replications. The lowest MSE is highlighted in bold in each scenario, and the second lowest MSE is highlighted in underline.      **S3 Fig. Type I error rate of MR-PL and other MR approaches in supplementary simulation with the presence of pleiotropy.** Simulation settings were included if the causal effect was drawn from the discrete set. A method fails to control type I error if its type I error rate exceeds the nominal significance level of 0.05 (red dotted line). The error bar represents the variance of type I error rate over 100 replications.    **S4 Fig. Sargan test *P*-value distribution of MR-PL in baseline simulation where no pleiotropy exists.** A histogram illustrating the distribution of *P*-values across 100 replications is presented for each parameter setting. A *P*-value exceeding 0.05 indicates the absence of pleiotropy.    **S5 Fig. Sargan test *P*-value distribution of MR-PL in supplementary simulation with the presence of pleiotropy.** A histogram illustrating the distribution of *P*-values across 100 replications is presented for each parameter setting. A *P*-value below 0.05 indicates the presence of pleiotropy.    **S6 Fig. Sargan test *P*-value distribution of MR-PL with WC-correction in baseline simulation where no pleiotropy exists.** A histogram illustrating the distribution of *P*-values across 100 replications is presented for each parameter setting. A *P*-value exceeding 0.05 indicates the absence of pleiotropy.    **S7 Fig. Sargan test *P*-value distribution of MR-PL with WC-correction in supplementary simulation with the presence of pleiotropy.** A histogram illustrating the distribution of *P*-values across 100 replications is presented for each parameter setting. A *P*-value below 0.05 indicates the presence of pleiotropy. |
| Excerpt 2.3 | **Lines 202-205 (Main text):**  In supplementary simulation with the presence of pleiotropy, the comparison was narrowed among the most focused approaches of MR-PL, Multi-2SLS and Uni-2SLS, where MR-PL slightly outperformed other methods in terms of MSE at most parameter settings (**S5 Table**).  **Lines 220-226 (Main text):**  In the presence of pleiotropy, all approaches exhibited a considerably inflated type I error rate larger than 0.2 (**S3 Fig**) when compared to simulations with no pleiotropy exists (**Fig 3A**). Despite this, the Sargan test used in our method successfully distinguished between scenarios with and without pleiotropy across all parameter settings (**S4-S7 Figs**). Therefore, we are able to recognize and remove out significant MR results that may be attributed to pleiotropy through combining the established pleiotropy-detecting capability of Sargan test with our method.  **Lines 572-576 (Main text):**  Second, our method cannot deal with the pleiotropy itself, and it relies on Sargan test to identify scenarios with the presence of global horizontal pleiotropy. A more efficient way is to incorporate the pleiotropy term in the model like MV-IWAS-Egger and PMR-Egger [25, 72]. In the future, we will take into account the pleiotropy by directly incorporating SNP effects into the second stage of our model.  **Lines 813-814 (Main text):**  Besides, the simulation study was also expanded to different correlation structure between instrumental SNPs, and scenarios with the presence of pleiotropy. More details are presented in the **S1 Text**.  **S1 text:**  **Supplementary simulation with the presence of pleiotropy**  For this supplementary simulation, we regenerated the outcome with the impact of direct pleiotropy from variants as follows by taking the prior study as a reference [1]: $Y= G\tau+\sum_{i=1}^{K} \beta_{x_{i}y}X_{i}+\sum_{i=1}^{K} \delta_{u_{i}y}U_{i}+\varepsilon_{y}$   where $\tau$ denotes the *m*-vector of SNP direct effects on the outcome ***Y***, which was generated from normal distribution $N(\mu_{\tau},\sigma_{\tau}^{2})$ with $\mu_{\tau}=0.2$ and $\sigma_{\tau}=0.05$. ***Y*** was then scaled to have a zero mean and unit variance. The remaining notations are consistent with their meanings in the main text. The genotype matrix, exposure matrix, confounding factors matrix, and the effect of exposures and confounding factors remained the same as in baseline simulation. Therefore, direct comparison can be made between each replication in our baseline simulation with no pleiotropy exists, and each replication in this supplementary simulation with pleiotropy exists. For simplicity, we narrowed the comparison among the most focused methods: MR-PL, Multi-2SLS and Uni-2SLS. |

**REF 2.4 – Reporting the conditional F-statistics**

| *Referee Comment* | *The F statistics was used to evaluate the IV strength for each exposure, however, in the multivariable context, a conditional F statistic is also suggested to use for evaluating weak IV issue (see [2]). The authors should also report the conditional F-statistics.*  *Ref:*  *[2] Eleanor Sanderson, Wes Spiller, and Jack Bowden. Testing and correcting for weak and pleiotropic instruments in two-sample multivariable mendelian randomization. Statistics in medicine, 40(25):5434–5452, 2021.* |
| --- | --- |
| Author Response | Thank you for the comment. Usually, the Sanderson-Windmeijer conditional F-statistic (*F_sw_*) is used to tested for the weak instruments problem in one-sample multivariable MR, and a *F_sw_* greater than the rule-of-thumb value of 10 means there is sufficient conditional instrument strength^1-3^. However, in our study, this canonical conditional F-statistic for testing the instrument strength in one-sample multivariable MR may not be applicable to our method, for which the reasons are shown as follows:  (1) The theory of conditional weak instrument F-test is basically built within the framework of 2SLS^2^, where exposures are fitted in a totally different way with our method. In 2SLS, each exposure is predicted separately and parallelly in the first stage, not considering the relationships with other exposures. In this event, when exposures are highly correlated with each other and instruments cannot be strongly associated with each exposure given other exposures, it could cause the issue of multicollinearity in predicted exposures and bias the causal estimates extremely (i.e., conditionally weak instruments bias). The conditional F-statistic is then designed to test this situation, which evaluates the overall strength of instruments on each individual exposure while controlling for other exposures^2^. In comparison, the PLS regression (and Lasso regression) used in our method is specially designed for alleviating the multicollinearity problems in predictor variables and response variables. Unlike 2SLS, PLS fits the regression model iteratively, where all exposures have already been considered as whole in each iterative step. In this context, each exposure is predicted while incorporating information from other exposures, effectively mitigating the impact of other highly correlated exposures, thus through the manner of ‘regressing out’ other exposures to test week instruments might not be suitable.  (2) The *F_sw_* value can hardly reach the rule-of-thumb value of 10 in high-dimension and high-correlation settings. We added to calculated the *F_sw_* statistics in our simulation. It shown that even in our ideal simulation with sufficient exposure-specific IVs, the *F_sw_* value was far from reaching 10 (with a mean of 4.37 before WC-correction, and 5.63 after WC-correction, **S23A Fig** and **S6 Table**), not to mention in real data applications (all *F_sw_* values were below 2 in our real data application, **S11 Table**).  (3) The *F_sw_* value is highly sensitive to the number of instruments and exposures, limiting its applicability in high-dimension scenarios. Since a larger parameter *c* value in our WC-correction procedure will induce less IVs and exposures, we altered the *c* values from 0 to 40 (step by 5) and calculated the corresponding *F_sw_* values (took the mean of 20 exposures) for all 43,200 simulations (100 replications × 48 settings × 9 *c* values). The results revealed a strong negatively correlation between *F_sw_* and both the number of exposures (*r* = -0.4, *P* < 5×10^-12^, Spearman correlation, **S23B Fig**), and the number of instruments (*r* = -0.21, *P* < 5×10^-12^, Spearman correlation, **S23C Fig**). Notably, we found that only when the number of instruments is fewer than 31 and the number of exposures is fewer than 12 can the *F_sw_* value entirely surpassed 10. When the number of exposures and instruments increased, the *F_sw_* value experienced a notable decline.  (4) Forcing to make the conditional F statistics larger than the rule-of-thumb value of 10 could lead to poor results of causal estimates. We segmented the *F_sw_* values into distinct bins for all the 43,200 simulations (100 replications × 48 settings × 9 *c* values), and investigated the performance of MR-PL within each bin. It shown that a higher *F_sw_* value is not consistently linked with improved performance of causal estimation, and simulations falling within the bin with *F_sw_* values excessed 10 gave the poorest performance in MSE and type I error rate control (**S23F-G Fig**). This is possibly because a higher *F_sw_* value indicates a reduced number of instruments and exposures (**S23B-E Fig**), therefore limiting the statistical capacity of MR in estimating causal effects, which is consistent with previous studies^4-9^.  In summary, the issue of weak instrument strength is too complicated and challenging in high dimension and high correlation scenarios. Actually, few studies have examined this issue in the cases of extreme upscaling the numbers of exposures and instruments. Although the current conditional F-statistic as well as its rule-of-thumb value for detecting weak instruments have some limitations, we acknowledge that our method cannot deal with the issue of detecting weak instruments in one-sample, high-dimension and high-correlation situations. Using only exposure-specific IVs as predictors like MV-IWAS might be one of the smart ways to address the problem. In the near future, we believe that more robust and data-driven statistical tools to evaluate the instrument strength and post-MR refining methodologies would be developed in cases of extreme upscaling the numbers of exposures and instruments. In the revised manuscript, we have followed the reviewer’s suggestion to report the conditional F-statistics in **S6 and S11 Tables**, as well as added to discuss this issue.    **S23 Fig. Results of conditional F-statistic values (*F_sw_*). (A)** Boxplot of the conditional F-statistics before and after WC-correction, with a mean of 4.37 before WC-correction, and 5.63 after WC-correction. **(B, D)** Scatter plots colored by density of the conditional F-statistics and exposure numbers across 43,200 simulations (100 replications × 48 settings × 9 *c* values), where **(D)** displays separate plots for different parameter settings of heritability. **(C, E)** Scatter plots colored by density of conditional F-statistics and instrumental SNP numbers across 43,200 simulations (100 replications × 48 settings × 9 *c* values), where **(E)** displays separate plots for different parameter settings of heritability. **(F)** A comparison of Mean squared error (MSE) for MR-PL across different bins of conditional F-statistic values. The error bar represents the variance of MSE. **(E)** A comparison of type I error rate for MR-PL across different bins of conditional F-statistic values. The error bar represents the variance of type I error rate. The red dotted line denotes the rule-of-thumb value of 10. *r*: the estimate of Spearman correlation; *P*: the *P*-value of Spearman correlation test; n_neighbors: the number of dots around each dot. |
| Excerpt 2.4 | **Lines 297-307 (Main text):**  Additionally, we examined the Sanderson-Windmeijer conditional F-statistics (*F_sw_*) values in our simulations [18, 27] (see **S1 Text** for more details). It shown that even in our ideal simulation with sufficient exposure-specific instruments, the *F_sw_* values can hardly reach the rule-of-thumb value of 10 (with a mean 4.37 before WC-correction, and 5.63 after WC-correction at *c*=20, **S23A Fig** and **S6 Table**). To investigate the simulation performance under different levels of conditional F-statistics, we segmented the corresponding *F_sw_* values into distinct bins for all the 43,200 simulations (100 replications × 48 settings × 9 *c* values varying from 0 to 40). It was observed that the *F_sw_* values were strongly negatively correlated with the number of instruments and exposures (**S23B-E Fig**). Moreover, simulations falling within the bin with *F_sw_* values excessed 10 gave the poorest performance in MSE and type I error control in our method (**S23F-G Fig**). These indicate the potential limitation of the conditional F-statistic in application of high-dimension and high-correlation situations.  **Lines 328-330 (Main text):**  The F-statistic and conditional F-statistic for the respective IDP exposure were reported in **S10 and S11 Tables**, respectively, wherein all F-statistics exceeded 10 and all conditional F-statistics were below 2.  **Lines 523-539 (Main text):**  The issue of weak instruments is often raised in multivariable MR, which is arguably very complex and challenging in high-dimension and high-correlation scenarios. According to our results, the conditional F-statistic *F_sw_* can hardly reach the rule-of-thumb value of 10 neither in our ideal simulations nor real data applications (**S23A Fig**, **S6 and S11 Tables**), and it is highly sensitive to the number of instruments and exposures (**S23B-E Fig**). Forcing to make it larger than 10 by reducing the number of instruments or exposures could result in poor causal estimation results (**S23E-F Fig**). Furthermore, the current conditional F-test used to evaluate weak instrument strength is basically built within the framework of 2SLS, where exposures are fitted in a totally different manner with our method in the first stage (2SLS: separately and parallelly; MR-PL: iteratively and considering all exposures as a whole). These indicate the potential limitation of the conditional F-statistic in evaluating instruments strength under one-sample, high-dimension and high-correlation setting, especially in our method. Currently, few studies have discussed this issue in cases of extreme upscaling the numbers of exposures and instruments, and most of them just constrained the analysis to a limited number of exposures. Incorporating dimensionality reduction techniques in MR or using only exposure-specific IVs as predictors might be one of the smart ways to solve this problem [25, 74, 75]. We believe that more robust and data-driven statistical tools to evaluate the instrument strength and post-MR refining methodologies would be developed in cases of extreme upscaling the numbers of exposures and instruments in the near future.  **S1 text:**  **Calculation of the conditional F-statistic**  Usually, the Sanderson-Windmeijer conditional F-statistic (*F_sw_*) is used to tested for the weak instruments problem in one-sample multivariable MR, and a *F_sw_* greater than the rule-of-thumb value of 10 means there is sufficient conditional instrument strength [2-4]. The calculation steps of *F_sw_* can be briefed as follows:  Step 1. Each exposure $X_{i}$ (1≤*i*≤*K*) is regressed on the whole set of genotype matrix $G$, and the predicted value of each exposure $\hat{X}_{i}$ (1≤*i*≤*K*) is calculated;  Step 2. Each exposure $X_{i}$ is regressed on all the other predicted exposures to yield the residual term $\varepsilon_{i}=X_{i}-\delta_{1}\hat{X}_{1}-...-\delta_{k}\hat{X}_{k(k\neq i)}-...-\delta_{K}\hat{X}_{K}$;  Step 3. The residual term corresponding to each exposure is then regressed on the whole set of genotype matrix $G$, and the conditional F-statistic *F_sw_* is obtained as the freedom adjusted F-statistic (*F*) in this regression as *F_sw_*= *F*×*M*/(*M*-*K*+1), where *M* denotes the total number of instrumental SNPs. |

**REF 2.5 – Writing issue**

| *Referee Comment* | *In the abstract, the authors stated 'MR is more likely to be used in a single large dataset...' It gave readers an impression that one-sample MR is more common. However, I believe two-sample MR is the more popular and convenient one in current practice. It'd better to rephrase this sentence. Furthermore, in page 4 line 95, the ref [16] can account for overlapping samples, but it is limited to a small number of exposures.* |
| --- | --- |
| Author Response | Thank you for your suggestion. In the revised manuscript, we have rephrased the sentence “MR is more likely to be used in a single large dataset …” in a more reasonable expression as: “While MR is currently more prevalent in two-sample settings, it is more promising to be used in a single large dataset due to the rise of biobank-scale datasets containing well-collected genotype data, brain imaging data, and matched complex traits.” (**Lines 20-22**)  We also checked the reference [16] in our original manuscript, and refined the statement of the sentence “Existing multivariable MR methods are generally designed on two-sample setting [11-16], or a small number of exposures [17-19]” in a more rigorous way as: “Existing multivariable MR methods are generally designed on two-sample setting [11-16], or a small number of exposures [16-19]. While the method MVMR-cML can account for sample-overlapping within the two-sample MR framework, its applicability is limited to a small number of exposures [16]” (**Lines 97-100**) |
| Excerpt 2.5 | Not applicable. |

**REF 2.6 – Writing issue / interpreting the results**

**REF 2.6.1**

| *Referee Comment* | *In the real data analysis,*  *Table S6 WC column only has ~21 categories while the analysis has 36 exposures, could the authors clarify this mismatch? Also, what does 'Average' in the WC column mean?* |
| --- | --- |
| Author Response | Thank you for the suggestion. Instead of directly using dMRI data provided by the UK Biobank, the GWAS summary statistics used for our IV selection provided by Zhao et al. (*Science, 2021*) reprocessed the dMRI data themselves in a more rigorous manner. Though Zhao et al. used the same brain atlas to segment white matter tracts as UK Biobank, they took the mean of measures for any left/right tract pairs rather than making a distinction between tracts in the left and right hemisphere. For example, in Zhao et al., only one type of white matter—the superior corona radiata—is presented, but both left and right superior corona radiata are provided by UK Biobank. Therefore, there were 20 individual tracts in Zhao et al. (**S9 Table**) corresponding to 36 tracts in UK Biobank (**S7 Table**). We have clarified the mismatch in the revised manuscript in **Lines 873-875**.  The term ‘Average’ refers to the mean FA value averaged across all tracts. It is a global dMRI measurement of the whole brain that captures the largest variation in white matter and has high heritability of 62.65%^25^. It is a crucial measurement which is always focused along with tract-specific measurements in relevant white matter studies^25-27^. Therefore, we believe SNPs linked to this global measurement could also help to capture variations of individual tracts and included this measurement into IV selection, even though there isn’t clear difference in IV number before (351) and after (368) including the Average measure. In the revised manuscript of manuscript, we have clarified the term ‘Average’ in **Lines 875-876** and the legend of **S9 Table**. |
| Excerpt 2.6.1 (main text) | **Lines 871-879:**  For IV selection, the GWAS summarized data with 33,292 British samples from the UKBB provided by Zhao et al. were used [28]. Since Zhao et al. took the mean of FA measures for any left/right tract pairs rather than making a distinction between tracts in the left and right hemisphere, there were 20 individual white matter tracts in Zhao et al. (**S9 Table**) corresponding to 36 ones in the UKBB (**S7 Table**). The global DTI measure of the mean FA value averaged across all tracts were also included for IV selection. Finally, 368 independent genetic variants (*r^2^* < 0.1, **S9 Table**) served as the initial IVs, which were reported to be associated with the mean FA value of at least one white matter tract at genome-wide significance level (5×10^-8^) from Zhao et al. |

**REF 2.6.2**

| *Referee Comment* | *In the real data analysis,*  *In Table 1, I noticed that only Eosinophill percentage had two putatively causal IDPs, while other traits only had one. Could the authors comment on this? In particular, it would be interesting to compare the results between MR-PL and the univariable analysis. By comparing Table S12 and S8, I found that most of the significant IDP-trait pair identified by MR-PL were not significant at all by univariable analysis (and other methods). Is this expected or something going wrong here?* |
| --- | --- |
| Author Response | Thank you for your suggestion. In the revised manuscript, we have added comment on the association between Eosinophill percentage and two putatively causal IDPs in **Lines 386-392**. We also added additional potential confounding factors as covariates to look at whether the association between Eosinophill percentage and the two IDPs still exists, including LDL cholesterol, HDL cholesterol, triglycerides, alcohol intake frequency and pack years of smoking, which have been long reported to be associated with blood cell composition^28; 29^. We observed that their putatively causal relationship still remained (*β* = 0.04 and *P* = 3.46×10^-2^ between anterior limb of internal capsule and Eosinophill percentage; *β* = -0.0002 and *P* = 3.64×10^-2^ between right uncinate fasciculus and Eosinophill percentage, **S14 Table**), indicating the robustness of their association.  We thought this is expected. The reason that some significant relationships identified by MR-PL were not significant by Uni-2SLS is possibly because only the significant SNPs specific to each individual exposure were used as IVs in Uni-2SLS, which could lack statistical power to detect causal relationships. For example, Uni-2SLS failed to detect the eight putatively causal relationships between cingulum hippocampus and different traits shown in **Table 1** (all with *P* > 0.05, **S20 Table**). In Uni-2SLS, only 11 SNPs were used as IVs to estimate the causal effects of the cingulum hippocampus on traits, whereas the number in multivariable MR is several to dozens of times higher. Given the reported high heritability of cingulum hippocampus (46%~57%^25; 30^), the limited number of SNPs may not be adequate to capture its variation. The reason that some significant relationships identified by MR-PL were not significant by Multi-2SLS is possibly because Multi-2SLS is always prone to weak instrument bias under high-dimensional and high correlation conditions, since it doesn’t consider the influence of other correlated exposures in the first stage of exposure prediction. For example, Multi-2SLS failed to detect any of the well-known associations between superior fronto-occipital fasciculus (SFO) and smoking^31; 32^ (*P*-value ranging from 0.43 to 0.97 for all SFO-smoking pairs, **S20 Table**). However, other highly correlated tracts such as fornix (*r* = 0.48/0.55 between fornix and right/left SFO) exhibited significant causal relationship with traits categorized in smoking, which is less evidenced for their association by prior literature. Despite some differences in significant results among these methods, their global tendency of causal estimations still remained consistent, where significant correlation of IDP-trait *β*-maps were found (*r* = 0.33 between MR-PL and Multi-2SLS, *r* = 0.53 between MR-PL and Uni-2SLS, *r* = 0.27 between Multi-2SLS and Uni-2SLS, all with *p* < 5×10^-5^, Spearman correlation). This implies that the difference in MR results produced by these approaches still lies within reasonable boundary. In the revised manuscript, we added additional texts to reported the results. |
| Excerpt 2.6.2 (main text) | **Lines 386-395:**  Two IDPs, the mean FA of the right uncinate fasciculus and anterior limb of the internal capsule, were found to be nominally associated with eosinophil percentage. It was reported that white matter lesions can trigger blood–brain barrier alterations [43], a plausible mechanism linking white matter damage and inflammation [44]. Such alterations will facilitate the migration of leukocytes, including eosinophils, within the bloodstream [45]. We expect that microstructure changes in these two tracts could contribute to blood–brain barrier alterations, thus potentially leading to eosinophils changes in blood. By adding new confounding factors as covariates which are potentially linked with blood cell composition and physical measures, including LDL cholesterol, HDL cholesterol, triglycerides, alcohol intake frequency and pack years of smoking, the above causal association pattern still remains (**S14 Table**).  **Lines 903-907:**  For traits categorized in biochemical and physical measures, sensitivity analysis was conducted by adding additional potential confounding factors as covariates, including LDL, HDL cholesterol, triglycerides, alcohol intake frequency and pack years of smoking, which have long been reported to be associated with blood cell composition and physical measurements [91-95].  **Lines 434-449:**  A consistent trend in causal estimations was observed across MR-PL, Uni-2SLS and Multi-2SLS (*r* = 0.33 between MR-PL and Multi-2SLS IDP-trait *β*-maps, *r* = 0.53 between MR-PL and Uni-2SLS IDP-trait *β*-maps, *r* = 0.27 between Multi-2SLS and Uni-2SLS IDP-trait *β*-maps, all with *P* < 5×10^-5^, Spearman correlation). Furthermore, MR-PL can reveal potential causal relationships that were not found by Uni-2SLS or Multi-2SLS. For example, Uni-2SLS failed to reveal the eight potential causal relationships between cingulum hippocampus and different traits shown in **Table 1**. This is possibly because only 11 SNPs were used as IVs to estimate the causal effect of cingulum hippocampus on traits in Uni-2SLS, and the limited number of SNPs may not be adequate to capture its variation given the reported high heritability of cingulum hippocampus (46%~57% [28, 63]). Multi-2SLS failed to detect the well-known association between SFO and smoking (*P*-value ranging from 0.43 to 0.97 for all SFO-smoking pairs, **S20 Table**). However, other highly correlated tracts such as fornix (*r*=0.48/0.55 between fornix and right/left SFO) exhibited significant causal relationship with traits categorized in smoking, which is less evidenced for their association by prior literature. This is possibly because Multi-2SLS is always prone to weak instrument bias under high-dimensional and high correlation conditions since it doesn’t consider the influence of other correlated exposures in the first stage of exposure prediction. |

**REF 2.7 – Figure drawing issue**

| *Referee Comment* | *Minor:*  *1. Fig 2. row 4 column 1, the red dot didn't have error bar. And what is the 10-fold variance of MSE?* |
| --- | --- |
| Author Response | Thank you for the comment. We have checked the red dot at row 4 column 1 in Fig. 2, and found that it indeed had an error bar, but is too small to be seen due to the extremely small variance (2.87×10^-4^). In the revised manuscript, we have clarified this in the legend of **Fig 2** as well as other figures having similar issues as “For settings with too small variance, the error bar tends to degenerate to a point.” The phrase “10-fold variance of MSE” means 10 × variance of MSE over 100 replications in each parameter setting. Because these variances are the too small to show clearly in the figure, we amplified all of them tenfold together. For example, if the MSE variance is 1×10^-3^, then its 10-fold is 1×10^-2^. |
| Excerpt 2.7 | Not applicable. |

**REF 2.8 – Writing issue**

| *Referee Comment* | *Minor:*  *page 19 line 384, could you clarify in 0.05/34/69, what is 34 and what is 69?* |
| --- | --- |
| Author Response | Thank you for your suggestion. In the revised manuscript, we have clarified “0.05/34/69” as “0.05/34/69 = 0.05 / number of IDPs retained after the WC-correction / number of variants retained after the WC-correction)” (**Lines 459-461**). |
| Excerpt 2.8 | Not applicable. |

**REF 2.9 – Writing issue**

| *Referee Comment* | *Minor:*  *page 27 line 575 'SNP I' should be 'SNP i'.* |
| --- | --- |
| Author Response | Thanks for pointing out this writing mistake. We have corrected “SNP *I*” as “SNP *i*” in the revised manuscript (**Line 716**). |
| Excerpt 2.9 | Not applicable. |

**REF 2.10 – Writing issue**

| *Referee Comment* | *Minor:*  *page 30 line 637, should it be 'N-'vector residual errors?* |
| --- | --- |
| Author Response | Yes, and thank you for pointing out this writing mistake. We have corrected “*M*-vector residual errors” as “*N*-vector residual errors” (**Line 778**). |
| Excerpt 2.10 | Not applicable. |

**REF 2.11 – Writing issue**

| *Referee Comment* | *Minor:*  *Could the authors clarify the leave-one-out analysis discussed in page 35 line 758 and page 19 line 382?* |
| --- | --- |
| Author Response | Thanks for the suggestion. Leave-one-out cross validation (LOOCV) is a special form of *N*-fold cross-validation, where *N* equals the number of samples in the dataset. Given that the PLS regression cannot calculate the standard error of estimate itself, we used LOOCV to assess the significance of coefficients in PLS regression. More specifically, when dealing with a dataset with 10,000 samples, one sample is held out as the validation set in each PLS model fitting, and the model is trained on the remaining data with 9,999 samples. The model fitting is then repeated 10,000 times, generating 10,000 sets of regression coefficients. Subsequently, the jackknife approximate *t* test is used to estimate coefficients variance and statistical significance^33^. The above procedure can be implemented through the following codes:  *pls.fit = plsr(exposure_matrix ~ genotype_matrix, ncomp = best_ncomp, validation = "LOO", jackknife = TRUE) jack_result = jack.test(pls.fit, ncomp = best_ncomp) p = as.data.frame(jack_result[["pvalues"]])*  In the revised manuscript, we have clarified the leave-one-out cross validation. |
| Excerpt 2.11 (main text) | **Lines 912-916:**  Specifically, the leave-one-out CV is a special form of *N*-fold CV, where *N* equals to the number of samples in the dataset. Following the implementation of leave-one-out CV in PLS regression, the coefficient variance and significance of SNPs with IDPs were derived through the jackknife approximate *t* test [96], which was implemented through *jack.test()* function within the R package pls (version 2.8-1). |

*Reviewer #3: Here the authors propose a new application for multivariable MR. The method is very similar to 2SLS but uses Partial least squares for the first step and LASSO for the second step. Moreover, the authors propose a correction for winners curse to be used in the one sample setting. Overall the paper applies mostly well know statistical techniques so there is not much to be said from the theoretical point of view.*

*So overall although I see the potential advantages of the methods I am not convinced by the presented data that it is developed enough and the below issues should be addressed.*

**REF3 – Response to REF3:** We would like to thank you for the thoughtful review of our manuscript, and your suggestions are very precious to our work. Our responses are summarized point-by-point below. We believe that the additional changes we have made improved our manuscript.

**REF3 – Point to point response to REF3**

**REF 3.1 – Winner’s curse correction**

| *Referee Comment* | *I have a slight issue with the winner’s curse correction as it doesn't seem to rely on a model apart to making the coefficient inversely proportional to sample size and -log(p). There is no simulation or any proof that this is actually proper through specific simulations so I am left unsure.* |
| --- | --- |
| Author Response | Thank you for your suggestion. The WC-correction presented here can be seen as a heuristic and data-driven approach rather than one grounded in strict theory, and it is informed by the previous study^34^. In the original manuscript, we have provided sufficient simulation and shown that the WC-correction procedure can further enhance the MR results in minimizing MSE and controlling type I error rate under a variety of sceneries, including different heritability, confounding factors strength, patterns of causal effects, correlating structure of instrumental variants and *P*-value thresholds employed for instrumental SNPs selection (**Figs 2 and 3; S2 and S4 Tables; S2, S15 and S16 Figs**). Our simple WC-correction can be seen as a secondary selection process of both the candidate instruments and exposures (**Lines 492-493**), which only requires the GWAS *P*-value of SNPs, thereby it can be fast and conveniently applied without adding any additional computational complexity. In the revised manuscript, we have made the following efforts to further enhance the reliability and conviction of this part.  (1) In the original manuscript, we used MSE to evaluate the performance of causal estimation, which is a combination of both estimation bias and variance. To better tell exactly where the benefit of MR-PL lies in causal estimation, we added to provide separate simulation results for bias and variance of causal estimation under each parameter setting (**S1 Fig** and **S3 Table**). It was observed that MR-PL consistently outperformed other approaches in yielding lower values of both bias and variance. More importantly, through the WC-correction procedure, our method generally achieved even lower values of bias and variance (**S1 Fig** and **S3 Table**). The results prove the effectiveness of our WC-correction procedure for causal estimation.    **S1 Fig. Bias and variance results of MR-PL and other MR approaches in baseline simulation.** Bias was calculated as the mean of the absolute difference between causal estimate and true effect, and variance was calculated as the variance of causal estimate minus true effect (averaged across 100 replications for each setting). The results from the methods of ImagingMR and PRS-2SLS were not included here. The full results can be found in **S3 Table**.  **S3 Table. Bias and variance results (values within parentheses) of MR-PL and other MR approaches in baseline simulation.** Bias was calculated as the mean of the absolute difference between causal estimate and true effect, and variance was calculated as the variance of causal estimate minus true effect (averaged across 100 replications for each setting). The lowest value is highlighted in bold in each scenario, and the second lowest value is highlighted in underline.    (2) In the original manuscript, we used the indexΔMSE/ΔIV for the selection of parameter *c* in WC-correction, hoping the WC-correction can lead to larger reduction in MSE while retaining more instruments. Given that incorporating more SNPs could also increase the potential for some irrelevant/non-significant SNPs included as predictors, we directly used ΔMSE as index, which is more intuitive, objective and less heuristic. The general pattern of index values is still consistent, with the optimal selection of *c* being 20, inducing the most significant decrease in MSE of 8.03%, followed by 15 or 25 (**Fig 3B**). Moreover, the Wilcoxon signed rank test^16^ revealed that the index value of ΔMSE at *c*=20 was significantly higher than that at *c*=15 and 25 across all 48 parameter settings (*P*=2.7×10^-5^ for *c*=15, and *P*=0.036 for *c*=25, **Fig 3B**). This indicates the robustness of the selection of parameter *c* in WC-correction.    **Fig 3B. The ΔMSE index at different tuning parameter *c* ranging from 5 to 40 (step by 5) in WC-correction.** ΔMSE denotes the percentage decrease in MSE from MR analysis without WC-correction to MR analysis with WC-correction, averaged across all 48 parameter settings. With the increase of ΔMSE, the greater the MSE was decreased after WC-correction. The Wilcoxon signed rank test was used to test the difference of ΔMSE between *c*=20 and its neighboring selections of 15 and 25 across all 48 parameter settings.  (3) To make the choice *c* in WC-correction more convincing, we added to provide simulation results at neighboring choices of *c*=15 and 25 under each individual parameter setting (**S9-S12 Figs**). It shown that the implementation of WC-correction at *c*=15 and 25 still led to the performance improvement of MR-PL as WC-correction at *c*=20, generally further reducing MSE and better controlling type I error rate (**S9-S12 Figs**). Such improvement of WC-correction at *c*=15 and 25 still held for different *P*-value thresholds for instrument selection including 5×10^-4^, 5×10^-5^, 5×10^-6^, 5×10^-7^, 5×10^-9^ and 5×10^-10^ (**S17-S20 Figs**). Therefore, it indicates that performance enhancement achieved via WC-correction procedure is not specific to a single choice of *c*, but is robust across a gird of neighboring values.    **S9 Fig.** **A comparison of mean squared error (MSE) between MR-PL with and without winner’s curse correction (WC-correction) at *c*=15 in baseline simulation.** The error bar represents ten-fold variance in MSE over 100 replications in each parameter setting. For settings with too small variance, the error bar tends to degenerate to a point.    **S10 Fig.** **A comparison of mean squared error (MSE) between MR-PL with and without winner’s curse correction (WC-correction) at *c*=25 in baseline simulation.** The error bar represents ten-fold variance in MSE over 100 replications in each parameter setting. For settings with too small variance, the error bar tends to degenerate to a point.    **S11 Fig.** **A comparison of type I error rate between MR-PL with and without winner’s curse correction (WC-correction) at *c*=15 in baseline simulation.** Simulation settings were included if the causal effect was drawn from the discrete set. The error bar represents the variance of type I error rate across 100 simulations for each parameter setting. For settings with too small variance, the error bar tends to degenerate to a point.    **S12 Fig.** **A comparison of type I error rate between MR-PL with and without winner’s curse correction (WC-correction) at *c*=25 in baseline simulation.** Simulation settings were included if the causal effect was drawn from the discrete set. The error bar represents the variance of type I error rate across 100 simulations for each parameter setting. For settings with too small variance, the error bar tends to degenerate to a point.    **S17 Fig.** **A comparison of mean squared error (MSE) between MR-PL with and without winner’s curse correction (WC-correction) at *c*=15 across different *P*-value thresholds to select instrumental variants in baseline simulation.** The error bar represents ten-fold variance in MSE over 100 replications in each parameter setting. For settings with too small variance, the error bar tends to degenerate to a point.    **S18 Fig.** **A comparison of mean squared error (MSE) between MR-PL with and without winner’s curse correction (WC-correction) at *c*=25 across different *P*-value thresholds to select instrumental variants in baseline simulation.** The error bar represents ten-fold variance in MSE over 100 replications in each parameter setting. For settings with too small variance, the error bar tends to degenerate to a point.    **S19 Fig.** **A comparison of type I error rate between MR-PL with and without winner’s curse correction (WC-correction) at *c*=15 across different *P*-value thresholds to select instrumental variants in baseline simulation.** Simulation settings were included if the causal effect was drawn from the discrete set. The error bar represents the variance of type I error rate across 100 simulations for each parameter setting. For settings with too small variance, the error bar tends to degenerate to a point.    **S20 Fig.** **A comparison of type I error rate between MR-PL with and without winner’s curse correction (WC-correction) at *c*=25 across different *P*-value thresholds to select instrumental variants in baseline simulation.** Simulation settings were included if the causal effect was drawn from the discrete set. The error bar represents the variance of type I error rate across 100 simulations for each parameter setting. For settings with too small variance, the error bar tends to degenerate to a point.  (4) We added to provide real data application results in the UKBB with WC-correction of neighboring selections of *c* at 15 and 25 (**S17 and S18 Tables**). High positive correlation of non-zero IDP-trait *β*-maps between the results of *c* at 20 and 15/25 were observed (*r* = 0.831/0.851, all with *P* < 5×10^-12^, Spearman correlation), and the associations between IDPs and smoking, blood cell composition and eating behaviors still remained nominal significant (*β* ≠ 0 and *P* < 0.05), implying that MR results with WC-correction in the real data application are robust to the selection of *c* around 20.  (5) We acknowledge that the MR performance under our WC-correction is not optimal and is dependent on multiple factors, including the genetic architecture of exposures, correlation pattern among exposures, linkage disequilibrium pattern of instrumental SNPs, and the ancestry of population. In the future, more accurate WC-correction methods tailored to MR with high dimension and high correlation situations can be developed, and the WC bias issue can also be entirely avoided by selecting IVs based on a different dataset^21^. In the revised manuscript, we added to discuss the limitation of our WC-correction (**Lines 563-568**). |
| Excerpt 3.1 (main text) | **Lines 195-199:**  Given MSE is a combination of bias and variance, we also provided separate simulation results for bias and variance of causal estimation. It shown that MR-PL typically yielded lower values for both bias and variance compared to other approaches in nearly all settings, followed by PLS-Elasticnet (**S1 Fig** and **S3 Table**). The results indicate that the benefit of MR-PL for causal estimation lies in both bias and variance.  **Lines 275-277:**  As depicted in **Fig 2** and **S2 Table**, MR-PL with WC-correction (*c* =20, not specified below) can further lower MSE across almost all scenarios when compared with the absence of WC-correction. Such reduction also applied to both bias and variance (**S1 Fig** and **S3 Table**).  **Lines 268-272:**  The index ΔMSE was used for the choice of optimal *c*, where ΔMSE denotes the percentage decrease in MSE from MR analysis without WC-correction to MR analysis with WC-correction. In simulation, *c* varied from 5 to 40 (step by 5), and the value with the highest average ΔMSE across all parameter settings at *P*-value threshold 5×10^-8^ was selected.  **Lines 274-275:**  When *c* was set to 20, MR-PL exhibited the most significant percentage decrease in MSE of 8.03% (**Fig 3B**).  **Lines 808-811:**  For the choice of *c*, the index ΔMSE was defined, where ΔMSE denotes the percentage decrease in MSE from MR analysis without WC-correction to MR analysis with WC-correction. The index ΔMSE was then averaged across all 48 parameter settings, and the optimal *c* was selected with the highest ΔMSE.  **Lines 281-284:**  Similar results were reported for neighboring selections of *c* at 15 and 25 (**S9-S14 Figs**), indicating that performance improvement achieved via WC-correction procedure is not specific to a single choice of *c*, but robust across a gird of neighboring values.  **Lines 292-294:**  The above conclusion that WC-correction can improve the performance of MR-PL in reducing MSE and type I error rate across various *P*-value thresholds for IV selection still held for neighboring selections of *c* at 15 and 25 (**S17-S20 Figs**), ….  **Lines 428-432:**  Analogously, significant positive correlation of *β*-maps were observed between the above main MR-PL results with WC-correction at *c*=20 and MR-PL results with WC-correction at neighboring selections of *c*=15/25 (*r* = 0.831/0.851, both with *P* < 5×10^-12^, Spearman correlation, **S17 and S18 Tables**), as well as MR-PL results without WC-correction (*r* = 0.792, *P* < 5×10^-12^, Spearman correlation, **S19 Table**).  **Lines 541-546:**  The WC-correction procedure presented here only requires the GWAS *P*-values of SNPs and has shown generally improved MR results across diverse scenarios including different heritability, confounding factors strength, patterns of causal effects, correlating structure of instrumental SNPs and *P*-value thresholds for IV selection, as well as robust results in both simulation and real data application. Therefore, it can be fast and conveniently applied without adding any additional computational complexity.  **Lines 563-568:**  It should be noted that our simple WC-correction is not optimal and is dependent on multiple factors, including the genetic architecture of exposures, correlation pattern among exposures, linkage disequilibrium pattern of instrumental SNPs, and the ancestry of population. In the future, more accurate WC-correction methods tailored to MR with high-dimension and high-correlation situations would be developed. With rapid accumulation of the biobank-scale datasets and GWAS summarized data, the WC bias issue can be entirely avoided by selecting IVs based on a different dataset [7]. |

**REF 3.2 – Providing power results**

| *Referee Comment* | *The method itself seems to be a bit uncalibrated as Type I error is much lower than expected. This I think is reflected by the real data application where out of 4420 tests only 26 have a p<0.05 instead of the expected >200. So probably the test is underpowered. I realise that the estimates seem to be more precise when point estimates are compared to the original ones (lower MSE), since p-values are used these seem larger than expected, so in this respect it is hard to see the advantage of this method. Proper simulated power analysis should be presented. In this context it is hard to evaluate the claimed results as in the end they could be the result of chance.* |
| --- | --- |
| Author Response | Thank you for your constructive suggestion. Here, we want to clarify more about why the *P*-values seem to be a bit inconsistent with the causal estimates in our method. In the second stage of our method, we applied Lasso regression to derive causal estimates, which always yields a sparse model with some coefficients set to zero. A disadvantage of such regularized regression approaches is that it can’t obtain the *P*-value of coefficients. To address the issue, we adopted the de-sparsified Lasso method, a widely used post-processing step following Lasso regression, which takes additional steps to re-estimate the statistical significance of non-zero coefficients^35; 36^. The causal estimation from Lasso regression and the *P*-value from de-sparsified Lasso method collaboratively determine whether a causal relationship exists. More specifically, a causal relationship exists only when the estimation doesn’t equal zero and the *P*-value is below a given threshold. Conversely, if the estimation equals zero or the *P*-value exceeds the threshold, the relationship is considered non-causal. Therefore, our method tends to give a bit stricter criterion to determine the causal relationship than standard linear regression. Since Lasso regression and de-sparsified Lasso method are based on two distinct theoretical frameworks, there can be some slightly inconsistency between the estimates and *P*-values. Nevertheless, such implementation is widely used, and the two methods are always mutually complementary to use in relevant literatures^37; 38^.  We followed the reviewer’s suggestion, and added to provide power results (represented as 1 - type II error rate at 5% nominal level) in **S8 Fig** in the revised manuscript. It shown that all the methods had similar power results, with the exception of PRS-2SLS which had low power under 0.5. The highest power results can be seen for Multi-2SLS, but at a cost of inflated type I error rate and large MSE (**Figs 2 and 3A**). MR-PL displayed overall high-power results ranging from 0.975 to 0.998, highlighting its ability to maintain substantial power while effectively controlling the type I error. Application of WC-correction can further increase the power of MR-PL, except for scenarios with low heritability (*hg^2^* = 0.2) where the power slightly reduced. Similar conclusions apply to the neighboring selections of *c* at 15 and 25 (**S13 and S14 Figs**). These results reflect that our method still remains high sensitivity to detect true causal relationships while controlling for type I error rate and minimizing MSE best. Moreover, in the real data application, the MR-PL analysis was repeated for correlated variants (*r^2^* < 0.6) as instrument (**S16 Table**), and with WC-correction at neighboring choices of *c*=15/25 (**S17 and S18 Tables**). The associations between IDPs and smoking, blood cell count and eating behaviors were still identified at *β* ≠ 0 and *P* < 0.05, indicating that the results cannot appear by chance. In the revised manuscript, we added the relevant power results.    **S8 Fig. Power results of MR-PL and other MR approaches in baseline simulation.** Simulation settings were included if the causal effect was drawn from the discrete sets. The error bar represents the variance of power over 100 replications in each parameter setting. For settings with too small variance, the error bar tends to degenerate to a point.    **Fig 2. Mean squared error (MSE) of MR-PL and other MR approaches.** The parameters were set to cover different exposures heritability *hg^2^*, different interference strength of confounding factors *α_ux_* and different types of causal effects *β_xy_* (**S1 Table**). Under each parameter setting, MSE was averaged over 100 replications. The error bar represents ten-fold variance of the MSE over 100 replications. For settings with too small variance, the error bar tends to degenerate to a point. Notably, the results from the methods of ImagingMR and PRS-2SLS were not included here because the MSE values from the two methods were extremely large to show here. The full results can be found in **S2 Table**.    **Fig 3A. A comparison of type I error rate across MR-PL and other MR approaches.** Simulation settings were included if the causal effect was drawn from the discrete set. MR methods were included if individual *P*-value of causal effect estimate of the respective exposure can be calculated. A method failed to control type I error if its type I error rate exceeded the nominal significance level of 0.05 (red dotted line). The error bar represents twice variance in type I error rate over 100 replications. For settings with too small variance, the error bar tends to degenerate to a point.    **S13 Fig.** **A comparison of power results between MR-PL with and without winner’s curse correction (WC-correction) at *c*=15 (and other approaches) in baseline simulation.** Simulation settings were included if the causal effect was drawn from the discrete set. The error bar represents the variance of power across 100 simulations for each parameter setting. For settings with too small variance, the error bar tends to degenerate to a point.    **S14 Fig.** **A comparison of power results between MR-PL with and without winner’s curse correction (WC-correction) at *c*=25 (and other approaches) in baseline simulation.** Simulation settings were included if the causal effect was drawn from the discrete set. The error bar represents the variance of power across 100 simulations for each parameter setting. For settings with too small variance, the error bar tends to degenerate to a point. |
| Excerpt 3.2 (main text) | **Lines 228-235:**  Power results (represented as 1 - type II error rate at 5% nominal level) of approaches were provided in **S8 Fig**. It shown that all the MR approaches had similar power results, except PRS-2SLS which had low power under 0.5 across all settings. The highest power results can be seen for Multi-2SLS, but at a cost of inflated type I error rate and large MSE (**Figs 2 and 3A**). MR-PL displayed overall high-power results ranging from 0.975 to 0.998, highlighting its ability to maintain substantial power while effectively controlling the type I error rate. These results suggest that our method still remains the sensitivity to detect true causal relationships while controlling for type I error rate and minimizing MSE best.  **Lines 281-284:**  Similar results were reported for neighboring selections of *c* at 15 and 25 (**S9-S14 Figs**), indicating that performance improvement achieved via WC-correction procedure is not specific to a single choice of *c*, but is robust across a gird of neighboring values. |

**REF 3.3 – Reliability of the findings between brain features and non-behavioral traits**

| *Referee Comment* | *Linking brain areas to behavior will basically always make sense, but other less. For example why would a specific brain area influence the width of platelets? I would try to at least find some positive controls to see if everything is working fine. For example Lipids, BMI, smoking and alcohol as exposures and CVD as outcome.* |
| --- | --- |
| Author Response | In the original manuscript, we have included BMI as one of the covariates in MR, a potential confounding factor which has been reported for its association with blood cell composition and physical measurements^39; 40^. In the revised manuscript, we added to look at the impact of more background traits as potential confounding factors on MR results, including LDL (Field ID 30780), HDL cholesterol (Field ID 30760), triglycerides (Field ID 30870), alcohol intake frequency (Field ID 1558), and pack years of smoking (Field ID 20161), which have long been reported to be associated with blood cell composition and physical measurements^28; 29; 41-43^. All potential confounding factors were first regressed out as covariates from the imaging exposures to remove their influence on the model. For the 9 IDP-trait pairs wherein the traits were categorized in biochemical measures and physical measures presented in **Table 1**, we noticed that the main results were almost the same (**R1 Table**). All of these pairs still stood with a non-zero causal effect estimate at *P* < 0.05, except that the causal estimation between position of the pulse wave peak and cingulum hippocampus did not quite reach but very close to the nominal significance (*β* =0.0061, *P* = 5.99×10^-2^). The association between right cingulum hippocampus and platelet distribution width still remained the most significant (*β* = 0.0653, *P* = 7.11×10^-5^). Furthermore, all the 9 IDP-trait pairs have successfully passed Sargan test no matter before and after adding additional potential confounders, indicating the absence of pleiotropy.  **R1 Table.** **MR results for the 9 IDP-trait pairs presented in Table 1, wherein the traits were categorized in biochemical and physical measures, with and without adding additional background traits as covariates.** The additionally added covariates includes LDL (Field ID 30780), HDL cholesterol (Field ID 30760), triglycerides (Field ID 30870), alcohol intake frequency (Field ID 1558), and pack years of smoking (Field ID 20161). The ‘Original results’ columns are the same as those shown in **Table 1**. The ‘Adding additional potential confounding factors as covariates’ columns are the same as those shown in **S14 Table**.    Since there are always unobserved confounding factors, through which variants exert a direct effect on the outcome outside any exposures, we added simulations to assess the effectiveness of the Sargan test used in our method for testing pleiotropy. By taking the prior study as a reference^24^, we regenerated the outcome with the impact of direct pleiotropy from variants as follows $Y= G\tau+\sum_{i=1}^{K} \beta_{x_{i}y}X_{i}+\sum_{i=1}^{K} \delta_{u_{i}y}U_{i}+\varepsilon_{y}$   Where $\tau$ denotes the *m*-vector of SNP direct effects on the outcome ***Y***, and was generated from normal distribution $N(\mu_{\tau},\sigma_{\tau}^{2})$ with $\mu_{\tau}=0.2$ and $\sigma_{\tau}=0.05$. ***Y*** was then scaled to have a zero mean and unit variance. The genotype matrix, exposure matrix, confounding factors matrix, and the effect of exposures and confounding factors remained the same as before. Therefore, direct comparison can be made between each replication in our baseline simulation with no pleiotropy exists, and each replication in this supplementary simulation with pleiotropy exists. The results shown that Sargan test successfully distinguished between scenarios with and without pleiotropy across all parameter settings, which is depicted in the Sargan test *P*-value histograms in **S4-S7 Figs**. Therefore, by combining the established pleiotropy-detecting capability of Sargan test with our method, we are able to recognize and eliminate significant MR results that may be attributed to pleiotropy, which could be caused by some unobserved confounding factors. For example, the relationship between right uncinate fasciculus and mean platelet (thrombocyte) volume failed to pass the Sargan test (**Line 5 in S12 Table**), implying that this significant result was influenced by pleiotropy and was thus discarded.    **S4 Fig. Sargan test *P*-value distribution of MR-PL in baseline simulation where no pleiotropy exists.** A histogram illustrating the distribution of *P*-values across 100 replications is presented for each parameter setting. A *P*-value exceeding 0.05 indicates the absence of pleiotropy    **S5 Fig. Sargan test *P*-value distribution of MR-PL in supplementary simulation with the presence of pleiotropy.** A histogram illustrating the distribution of *P*-values across 100 replications is presented for each parameter setting. A *P*-value below 0.05 indicates the presence of pleiotropy.    **S6 Fig. Sargan test *P*-value distribution of MR-PL with WC-correction in baseline simulation where no pleiotropy exists.** A histogram illustrating the distribution of *P*-values across 100 replications is presented for each parameter setting. A *P*-value exceeding 0.05 indicates the absence of pleiotropy.    **S7 Fig. Sargan test *P*-value distribution of MR-PL with WC-correction in supplementary simulation with the presence of pleiotropy.** A histogram illustrating the distribution of *P*-values across 100 replications is presented for each parameter setting. A *P*-value below 0.05 indicates the presence of pleiotropy.  In summary, these results confirm the robustness of our results in the real data applications. In the revised manuscript, we reported the above results. |
| Excerpt 3.3 | **Lines 392-395 (Main text):**  By adding new confounding factors as covariates which are potentially linked with blood cell composition and physical measures, including LDL cholesterol, HDL cholesterol, triglycerides, alcohol intake frequency and pack years of smoking, the above causal association pattern still remains (**S14 Table**).  **Lines 903-907 (Main text):**  For traits categorized in biochemical and physical measures, sensitivity analysis was conducted by adding additional potential confounding factors as covariates, including LDL, HDL cholesterol, triglycerides, alcohol intake frequency and pack years of smoking, which have long been reported to be associated with blood cell composition and physical measurements [91-95].  **Lines 222-226 (Main text):**  Despite this, the Sargan test used in our method successfully distinguished between scenarios with and without pleiotropy across all parameter settings (**S4-S7 Figs**). Therefore, we are able to recognize and remove out significant MR results that may be attributed to pleiotropy through combining the established pleiotropy-detecting capability of Sargan test with our method.  **Lines 813-814 (Main text):**  Besides, the simulation study was also expanded to different correlation structure between instrumental SNPs, and scenarios with the presence of pleiotropy. More details are presented in the **S1 Text**.  **S1 text:**  **Supplementary simulation with the presence of pleiotropy**  For this supplementary simulation, we regenerated the outcome with the impact of direct pleiotropy from variants as follows by taking the prior study as a reference [1]: $Y= G\tau+\sum_{i=1}^{K} \beta_{x_{i}y}X_{i}+\sum_{i=1}^{K} \delta_{u_{i}y}U_{i}+\varepsilon_{y}$   where $\tau$ denotes the *m*-vector of SNP direct effects on the outcome ***Y***, which was generated from normal distribution $N(\mu_{\tau},\sigma_{\tau}^{2})$ with $\mu_{\tau}=0.2$ and $\sigma_{\tau}=0.05$. ***Y*** was then scaled to have a zero mean and unit variance. The remaining notations are consistent with their meanings in the main text. The genotype matrix, exposure matrix, confounding factors matrix, and the effect of exposures and confounding factors remained the same as in baseline simulation. Therefore, direct comparison can be made between each replication in our baseline simulation with no pleiotropy exists, and each replication in this supplementary simulation with pleiotropy exists. For simplicity, we narrowed the comparison among the most focused methods: MR-PL, Multi-2SLS and Uni-2SLS. |

**References**

1. Sanderson, E., Spiller, W., and Bowden, J. (2021). Testing and correcting for weak and pleiotropic instruments in two-sample multivariable Mendelian randomization. Statistics in medicine 40, 5434-5452.

2. Sanderson, E., and Windmeijer, F. (2016). A weak instrument [Formula: see text]-test in linear IV models with multiple endogenous variables. J Econom 190, 212-221.

3. Sanderson, E., Davey Smith, G., Windmeijer, F., and Bowden, J. (2019). An examination of multivariable Mendelian randomization in the single-sample and two-sample summary data settings. International journal of epidemiology 48, 713-727.

4. Brion, M.J., Shakhbazov, K., and Visscher, P.M. (2013). Calculating statistical power in Mendelian randomization studies. International journal of epidemiology 42, 1497-1501.

5. Porcu, E., Rueger, S., Lepik, K., e, Q.C., Consortium, B., Santoni, F.A., Reymond, A., and Kutalik, Z. (2019). Mendelian randomization integrating GWAS and eQTL data reveals genetic determinants of complex and clinical traits. Nature communications 10, 3300.

6. Burgess, S., Small, D.S., and Thompson, S.G. (2017). A review of instrumental variable estimators for Mendelian randomization. Stat Methods Med Res 26, 2333-2355.

7. Burgess, S., Butterworth, A., and Thompson, S.G. (2013). Mendelian randomization analysis with multiple genetic variants using summarized data. Genet Epidemiol 37, 658-665.

8. Burgess, S., Dudbridge, F., and Thompson, S.G. (2016). Combining information on multiple instrumental variables in Mendelian randomization: comparison of allele score and summarized data methods. Statistics in medicine 35, 1880-1906.

9. Burgess, S., and Thompson, S.G. (2011). Bias in causal estimates from Mendelian randomization studies with weak instruments. Statistics in medicine 30, 1312-1323.

10. Karageorgiou, V., Gill, D., Bowden, J., and Zuber, V. (2023). Sparse dimensionality reduction approaches in Mendelian randomisation with highly correlated exposures. Elife 12.

11. Brouwer, R.M., Klein, M., Grasby, K.L., Schnack, H.G., Jahanshad, N., Teeuw, J., Thomopoulos, S.I., Sprooten, E., Franz, C.E., Gogtay, N., et al. (2022). Genetic variants associated with longitudinal changes in brain structure across the lifespan. Nat Neurosci 25, 421-432.

12. Kochunov, P., Glahn, D.C., Lancaster, J., Thompson, P.M., Kochunov, V., Rogers, B., Fox, P., Blangero, J., and Williamson, D.E. (2011). Fractional anisotropy of cerebral white matter and thickness of cortical gray matter across the lifespan. Neuroimage 58, 41-49.

13. Zhao, X., Yang, A., Zhang, Z.C., Yang, Y.T., and Zhao, X.M. (2023). Deciphering the genetic architecture of human brain structure and function: a brief survey on recent advances of neuroimaging genomics. Briefings in bioinformatics 24.

14. Zuber, V., Colijn, J.M., Klaver, C., and Burgess, S. (2020). Selecting likely causal risk factors from high-throughput experiments using multivariable Mendelian randomization. Nature communications 11, 29.

15. Lin, Z., Xue, H., and Pan, W. (2023). Robust multivariable Mendelian randomization based on constrained maximum likelihood. Am J Hum Genet.

16. Bauer, D.F. (1972). Constructing confidence sets using rank statistics. Journal of the American Statistical Association 67, 687-690.

17. Mevik, B.H., and Wehrens, R. (2007). The pls package: Principal component and partial least squares regression in R. J Stat Softw 18, 1-23.

18. Si, S., and Sun, Z. (2015). Mathematical Modeling Algorithms and Applications.(National Defense Industry Press).

19. Tibshirani, R. (1996). Regression shrinkage and selection via the Lasso. J Roy Stat Soc B Met 58, 267-288.

20. Zhu, Z., Zhang, F., Hu, H., Bakshi, A., Robinson, M.R., Powell, J.E., Montgomery, G.W., Goddard, M.E., Wray, N.R., Visscher, P.M., et al. (2016). Integration of summary data from GWAS and eQTL studies predicts complex trait gene targets. Nat Genet 48, 481-487.

21. Burgess, S., Davey Smith, G., Davies, N.M., Dudbridge, F., Gill, D., Glymour, M.M., Hartwig, F.P., Kutalik, Z., Holmes, M.V., Minelli, C., et al. (2019). Guidelines for performing Mendelian randomization investigations: update for summer 2023. Wellcome Open Res 4, 186.

22. Wang, L., Jia, P., Wolfinger, R.D., Chen, X., Grayson, B.L., Aune, T.M., and Zhao, Z. (2011). An efficient hierarchical generalized linear mixed model for pathway analysis of genome-wide association studies. Bioinformatics 27, 686-692.

23. de Leeuw, C.A., Mooij, J.M., Heskes, T., and Posthuma, D. (2015). MAGMA: generalized gene-set analysis of GWAS data. PLoS Comput Biol 11, e1004219.

24. Knutson, K.A., Deng, Y., and Pan, W. (2020). Implicating causal brain imaging endophenotypes in Alzheimer's disease using multivariable IWAS and GWAS summary data. Neuroimage 223, 117347.

25. Zhao, B., Li, T., Yang, Y., Wang, X., Luo, T., Shan, Y., Zhu, Z., Xiong, D., Hauberg, M.E., Bendl, J., et al. (2021). Common genetic variation influencing human white matter microstructure. Science (New York, NY) 372.

26. Dekkers, I.A., Jansen, P.R., and Lamb, H.J. (2019). Obesity, Brain Volume, and White Matter Microstructure at MRI: A Cross-sectional UK Biobank Study. Radiology 291, 763-771.

27. Cremers, L.G.M., Wolters, F.J., de Groot, M., Ikram, M.K., van der Lugt, A., Niessen, W.J., Vernooij, M.W., and Ikram, M.A. (2020). Structural disconnectivity and the risk of dementia in the general population. Neurology 95, e1528-e1537.

28. Tziakas, D., Chalikias, G., Grapsa, A., Gioka, T., Tentes, I., and Konstantinides, S. (2012). Red blood cell distribution width: a strong prognostic marker in cardiovascular disease: is associated with cholesterol content of erythrocyte membrane. Clin Hemorheol Microcirc 51, 243-254.

29. Snell, L.D., Glanz, J., and Tabakoff, B. (2002). Relationships between effects of smoking, gender, and alcohol dependence on platelet monoamine oxidase-B: activity, affinity labeling, and protein measurements. Alcoholism, clinical and experimental research 26, 1105-1113.

30. Lee, S.J., Steiner, R.J., Luo, S., Neale, M.C., Styner, M., Zhu, H., and Gilmore, J.H. (2015). Quantitative tract-based white matter heritability in twin neonates. Neuroimage 111, 123-135.

31. Liang, H., Chang, L., Chen, R., Oishi, K., and Ernst, T. (2018). Independent and Combined Effects of Chronic HIV-Infection and Tobacco Smoking on Brain Microstructure. J Neuroimmune Pharmacol 13, 509-522.

32. Weng, J.C., Chuang, Y.C., Zheng, L.B., Lee, M.S., and Ho, M.C. (2022). Assessment of brain connectome alterations in male chronic smokers using structural and generalized q-sampling MRI. Brain Imaging Behav 16, 1761-1775.

33. Martens, H., and Martens, M. (2000). Modified Jack-knife estimation of parameter uncertainty in bilinear modelling by partial least squares regression (PLSR). Food Quality and Preference 11, 5-16.

34. Shi, J., Park, J.H., Duan, J., Berndt, S.T., Moy, W., Yu, K., Song, L., Wheeler, W., Hua, X., Silverman, D., et al. (2016). Winner's Curse Correction and Variable Thresholding Improve Performance of Polygenic Risk Modeling Based on Genome-Wide Association Study Summary-Level Data. PLoS Genet 12, e1006493.

35. Zhang, C.H., and Zhang, S.S. (2014). Confidence intervals for low dimensional parameters in high dimensional linear models. Journal of the Royal Statistical Society, Series B Statistical Methodology.

36. Dezeure, R., Bühlmann, P., Meier, L., and Meinshausen, N. (2015). High-dimensional inference: confidence intervals, p-values and R-software hdi. Statistical science, 533-558.

37. Priya, S., Burns, M.B., Ward, T., Mars, R.A.T., Adamowicz, B., Lock, E.F., Kashyap, P.C., Knights, D., and Blekhman, R. (2022). Identification of shared and disease-specific host gene-microbiome associations across human diseases using multi-omic integration. Nat Microbiol 7, 780-795.

38. Lang, B.M., Ledergerber, M., Jordi, S.B.U., Krupka, N., Biedermann, L., Schreiner, P., Juillerat, P., Wyss, J., Vavricka, S.R., Zeitz, J., et al. (2023). Because I'm happy - positive affect and its predictive value for future disease activity in patients with inflammatory bowel diseases: a retrospective cohort study. Therapeutic advances in gastroenterology 16, 17562848231179335.

39. Church, T.S., Finley, C.E., Earnest, C.P., Kampert, J.B., Gibbons, L.W., and Blair, S.N. (2002). Relative associations of fitness and fatness to fibrinogen, white blood cell count, uric acid and metabolic syndrome. Int J Obes Relat Metab Disord 26, 805-813.

40. Hudson, L.D., Rapala, A., Khan, T., Williams, B., and Viner, R.M. (2015). Evidence for contemporary arterial stiffening in obese children and adolescents using pulse wave velocity: A systematic review and meta-analysis. Atherosclerosis 241, 376-386.

41. Urbina, E.M., Khoury, P.R., McCoy, C.E., Dolan, L.M., Daniels, S.R., and Kimball, T.R. (2013). Triglyceride to HDL-C ratio and increased arterial stiffness in children, adolescents, and young adults. Pediatrics 131, e1082-1090.

42. Del Giorno, R., Maddalena, A., Bassetti, S., and Gabutti, L. (2022). Association between Alcohol Intake and Arterial Stiffness in Healthy Adults: A Systematic Review. Nutrients 14.

43. Doonan, R.J., Hausvater, A., Scallan, C., Mikhailidis, D.P., Pilote, L., and Daskalopoulou, S.S. (2010). The effect of smoking on arterial stiffness. Hypertension research : official journal of the Japanese Society of Hypertension 33, 398-410.
