# Supplementary material for: An augmented Mendelian randomization approach provides causality of brain imaging features on complex traits in a single biobank-scale dataset
Source: PLoS Genet. 2023 Dec 27;19(12):e1011112. doi: 10.1371/journal.pgen.1011112 (PMC10775988; doi:10.1371/journal.pgen.1011112)
Supplement: Attachment — Submitted filename: Response to Reviewers.Round2.docx [file pgen.1011112.s053.docx]

Institute of Science and Technology for Brain-Inspired Intelligence

Fudan University

Shanghai 200433, China

November 17, 2023

Dear Editor,

Thank you for your feedback on our manuscript entitled “**An augmented Mendelian randomization approach provides causality of brain imaging features on complex traits in a single biobank-scale dataset**” (PGENETICS-D-23-00635R1). We sincerely thank all reviewers for their constructive and valuable feedback that we have used to improve the quality of our manuscript.

The reviewers’ comments have been numbered and laid out in italicized font, while our point-to-point response in normal font. Notably, any changes or modifications in the main text have been given in red.

We have made tremendous efforts in revising the manuscript. Hopefully, the revised manuscript would be regarded as appropriate for publication in PLOS Genetics. Thank you again for your consideration.

Best regards,

**Dr. Xing-Ming Zhao**

Professor

Institute of Science and Technology for Brain-Inspired Intelligence

Fudan University

Shanghai, P.R. China

Tel. 86-21-55665546

Email: xmzhao@fudan.edu.cn

*Response Letter to*

**An augmented Mendelian randomization approach provides causality of brain imaging features on complex traits in a single biobank-scale dataset**

*Reviewer #1:  It was interesting reading the review, the authors have put in extensive work in updating it.*

**REF1 – Response to REF1:** We sincerely thank you for taking the time to review our work. Your comments are valuable to us, and we have taken them into consideration for any further improvements. The detailed point-by-point responses are listed below.

**REF1 – Point to point response to REF1**

**REF 1.1 – Conditional instrument strength**

| *Referee Comment* | *I would add that it's more of the strength of association, that is the magnitude of the parameter chosen in the sim's, that guides the instrument strength, a stronger predictor of instrumental strength than number. Still I agree with the conclusion of the paragraph 297-307.* |
| --- | --- |
| Author Response | Thank you for this constructive comment. We agree with you that the magnitude of the parameter chosen in the simulation, in addition to the exposure and the number of instruments, could be one of the strong predictors guiding the instrumental strength, e.g., the proportion of variance in the respective exposure explained by the instruments (*hg^2^*). A wider range of parameter chosen could be applied to more thoroughly explore to the variables affecting instrument strength. In the revised manuscript, we have added comments on this in **Discussion**. |
| Excerpt 1.1 | **Lines 578-581 (Main text):**  Finally, in addition to the number of exposures and instruments, the magnitude of the parameter chosen in the simulation (e.g., *hg^2^*), could be one of the strong predictors that guiding the instrumental strength. More detailed studies with a wider range of parameters can look into the factors affecting instrument strength. |

**REF 1.2 – Revision feedback from the previous round**

| *Referee Comment* | *Appreciated the clarification on other types of more modifiable exposures and the discussion on brain tissue dynamics.* |
| --- | --- |
| Author Response | Thank you for this comment, and we appreciate your positive feedback. |
| Excerpt 1.2 | Not applicable. |

**REF 1.3 – Data generation framework**

| *Referee Comment* | *(1) L195-199: If you could add a specification of the exact data generating mechanism for clarity purposes that would be useful.*  *(2) How is correlation achieved? Due to genetics?* |
| --- | --- |
| Author Response | (1) Thank you for this suggestion. In the revised manuscript, we have provided a schematic in **S1 Fig** to more clearly show the data generation framework.    **S1 Fig. The data generation framework in baseline simulation.**  (2) In simulation, the correlation between two exposures was achieved through two components: genetic factors and confounding factors. Specifically, 250 of the 5,000 total SNPs (i.e., probability of 0.05) were independently and randomly selected as the causal SNPs for each of the 20 exposures. Such data generation framework could naturally induce an overlapping percentage of the causal/instrumental SNPs ranging from 0% to 18.2% between any two exposures (**S23 Table**), which would contribute to the correlation between two exposures (i.e., due to genetic factors). Furthermore, for each pair of exposures, *x_i_* and *x_j_*, both would be influenced by the common confounding factors *u_1_*, …, *u_i-1_*, *u_i+1_*, …, *u_j-1_*, *u_j+1_*, …, *u_20_*, which would further contribute to the correlation between exposures (i.e., due to confounding factors). |
| Excerpt 1.3 (main text) | **Line 164:**  The data generation mechanism in simulation is depicted in **S1 Fig**. |

**REF 1.4 – Writing issue**

| *Referee Comment* | *The term β-map is a bit unclear. Ι understand you refer to the many-element collection of the IDP-outcome effect estimates? You can define it in its first occurrence.* |
| --- | --- |
| Author Response | Thank you for this valuable suggestion. In the revised manuscript, we have added to define this term in its first occurrence. |
| Excerpt 1.4 (main text) | **Lines 426-428:**  Their pattern of *β*-maps (a vector that containing all non-zero causal estimates of IDPs on traits) was highly consistent (*r* = 0.91, *P* < 5×10^-12^, Spearman correlation) between the results from the two instrument selection strategies… |

**REF 1.5 – Writing issue**

| *Referee Comment* | *Lines 563+: I still find it confusing how exactly the developed WC correction method works. It could be of use if the authors added a couple of sentences that summaries this part of the workflow in plain language. Especially how the end-output is apparently more precise; I would imagine that the SEs of the falsely selected IVs would be inflated afterwards.* |
| --- | --- |
| Author Response | Thank you for the valuable suggestion. In the revised manuscript, we have added some plain texts to summarize this part, as well as highlighted how this method leads to a more precise end-output. |
| Excerpt 1.5 (main text) | **Lines 556-561:**  The WC-correction procedure can be considered as a secondary selection of candidate instruments (and exposures). It can further refine the selection boundary, significantly reducing the number of falsely selected IVs (**S26 Fig**). This process tries to achieve a delicate balance between reducing the inclusion of non-causal SNPs while retaining sufficient genetic information for analysis. Consequently, this adjustment would mitigate potential bias in exposure prediction and reduce causal estimate inflation thereafter. |

**REF 1.6 – Revision feedback from the previous round**

| *Referee Comment* | *Lines 684+: this passage spells out the difference of pca and pls in mr.* |
| --- | --- |
| Author Response | Thank you for this comment, and we appreciate your positive feedback. |
| Excerpt 1.6 | Not applicable. |

*Reviewer #2: Thank you for the revisions made to this paper, most of my comments have been resolved. However, I still have some remaining concerns.*

**REF2 – Response to REF2:** Thank you for taking the time to review our revised paper, and we're pleased to hear that most of your concerns have been addressed. We appreciate your thorough evaluation. We hope that the remaining concerns are appropriately addressed in this round of revision. The detailed point-by-point responses are listed below.

**REF2 – Point to point response to REF2**

**REF 2.1 – Type I error**

| *Referee Comment* | *In the ideal scenario simulation, univariable MR had inflated type-I error was expected, but for other multivariable methods, even 20 exposures are all included, it still had inflated type-I error. Did the authors explore why other multivariable MR methods and for some scenarios MR-PL had inflated type-I error even in the ideal scenario without pleiotropy and independent IVs (so no pleiotropy through LD as well)? Relatedly, how did the author define a type-I error? For example, for 20 exposures, 5 of them are non-causal, then it is counted a type-I error as long as one of the 5 p-values is smaller than 0.05? Similarly, how did the author define power when you have 15 causal exposures?* |
| --- | --- |
| Author Response | Thank you for your comments. Here, we would like to clarify that there are two reasons why other multivariable MR methods, including Multi-2SLS and MV-IWAS, exhibited inflated type-I error rates even in the ideal simulation, while MR-PL did not in most simulation scenarios.  First, unlike MR-PL, other multivariable MR methods cannot effectively address correlations among the exposures during the prediction process of stage I, which might result in inflated type I error rate in stage II. Given that the aim of our study is to investigate how to infer the causal relationships from multiple correlated exposures, the data generation mechanism in simulation was tailored to yield moderate to high correlations between any two exposures (ranging from 0.31 to 0.72) (see **S23 Table**, **Lines 793-795**). Therefore, the multivariable MR methods that cannot address these correlations are more likely to yield inflated results.  Second, MR-PL offers a slightly stricter criterion for determining the causal relationships than other multivariable methods. In MR-PL, Lasso regression is applied to derive causal estimates in stage II, which can always yield a sparse model with some coefficients set to zero. A disadvantage of such regularized regression approaches is that we cannot obtain the *P*-value of coefficients using these methods. To address this issue, we adopted the de-sparsified Lasso method, a widely used post-processing step following Lasso regression, which takes additional steps to re-estimate the statistical significance of non-zero coefficients [1,2]. Therefore, the causal estimation from Lasso regression and the *P*-value from de-sparsified Lasso method collaboratively determine whether a causal relationship exists in MR-PL (a causal relationship exists only when the estimation ≠ zero and *P*-value < a given threshold, **Lines 675-676**), while only the *P*-value is used in other multivariable MR methods.  As shown in **Fig 3A**, MR-PL just showed inflated type I error rate under two parameter settings when *β_xy_* was drawn from set {-0.1,0,0.1} (weak causal effects setting, **S1 Table**). The reason could be that as the Lasso regression was used in our method tends to provide sparse solution, it will make it difficult to distinguish between causal effects that are to each other (**Lines 212-213**).  The estimation of the type I error rate you mentioned is approximately accurate. More specifically, in MR-PL, the type I error rate was calculated as: (number of non-causal exposure-outcome pairs with causal estimate≠0 and *P* < 0.05) / number of non-causal exposure-outcome pairs. While in other multivariable MR methods including Multi-2SLS and MV-IWAS, the type I error rate was calculated as: (number of non-causal exposure-outcome pairs with *P* < 0.05) / number of non-causal exposure-outcome pairs. Then the type I error rate was averaged across 100 replications (the number of non-causal relationships in each replication is not fixed) under each parameter setting, and the averaged type I error rate larger than 0.05 can be considered as a type I error.  Similarly, in MR-PL, the power was calculated as: 1 - (number of causal exposure-outcome pairs with causal estimate = 0 or *P* ≥ 0.05) / number of causal exposure-outcome pairs. While in other multivariable MR methods including Multi-2SLS and MV-IWAS, the power was calculated as 1 - (number of causal exposure-outcome pairs with *P* ≥ 0.05) / number of causal exposure-outcome pairs. Then the power was averaged across 100 replications under each parameter setting. |
| Excerpt 2.1 | Not applicable. |

**REF 2.2 – Winner’s curse correction**

*While I appreciate the authors made a huge effort to revise the part of winner's curse correction, it is still not convincing enough for me.*

**REF 2.2.1**

| *Referee Comment* | *First, the intuition behind 'Select c with the lowest prediction R2 of outcome in MR from the grid search range.' was not clear. It seems to me the negative correlation between R^2 and reduction in MSE simply because when R^2 is large, correct for winner's curse or not didn't matter a lot, or the original method performed well enough in some sense. Could the authors verify this? S25 Figure was confusing - was the difference in R^2 and reduction in MSE driven by different c? Or the difference in R^2 was driven by different simulation setup? Then I am confused by how to select c based on R^2. Moreover, did the author check their suggested solution on real data?* |
| --- | --- |
| Author Response | Thank you for this constructive suggestion, and we are sorry for the confusion. Previously, the intuition ‘Select *c* with the lowest prediction *R^2^* of outcome in MR from the grid search range’ was informed by the parallel post-GWAS field of PRS, where the pre-defined *P*-value threshold used for SNP selection was determined by the prediction *R^2^* of the trait [3].  Here, we reconsidered and double checked this part carefully. In the original method without WC-correction, negative correlation was observed between *R^2^* and MSE (*r* = -0.48, *P* = 4×10^-4^, Spearman correlation, **R1A Fig**). It means that if the same instruments were used, when the prediction *R^2^* is high, the method tended to estimate the causal effect well to some sense as you mentioned. However, when applying WC-correction, we observed that the original prediction *R^2^* showed no correlation at all with the relationship between prediction *R^2^* and MSE reduction (*P* = 0.93, Spearman correlation, **R1B Fig**). This result implies that the relative value of the original prediction *R^2^* is not critical in the negative correlation between prediction *R^2^* and MSE reduction after applying WC-correction.    **R1 Fig. (A) Scatter plots of MSE and the prediction *R^2^* of MR-PL without applying WC-correction.** Each dot denotes a parameter setting (averaged across 100 replications). **(B) Scatter plots of the prediction *R^2^* of MR-PL without applying WC-correction and the Spearman estimate (test across different *c* values) between the prediction *R^2^* and MSE reduction when applying WC-correction.** Each dot denotes a parameter setting (averaged across 100 replications). *r*: the estimate of Spearman correlation; *P:* the *P*-value of Spearman correlation test.  We also redraw the original **S25 Fig** under separate parameter settings (**R2 Fig**) and *c* values (**R3 Fig**). It was discovered that under a majority of parameter settings (35 out of 48), *R^2^* had a negative correlation estimate with MSE reduction (**R2 Fig**). In the remaining 13 parameter settings with a positive correlation estimate, the majority of them were not statistically significant. These results suggest that the negative correlation between *R^2^* and MSE reduction is driven by different *c* values. As shown in **R3 Fig**, when the value *c* ranged from 10 to 35, the prediction *R^2^* was negatively correlated with MSE reduction. This result suggests that the negative correlation between *R^2^* and MSE reduction is driven by different parameter settings. As a result, the negative correlation between *R^2^* and MSE reduction is caused by both different *c* selections and simulation parameter settings.    **R2 Fig. Separate scatter plots under different parameter settings of MSE reduction and prediction *R^2^* when applying WC-correction.** The reduction in MSE was calculated as the MSE of MR analysis without WC-correction minus the MSE of MR analysis with WC-correction. Each dot denotes a *c*-value setting (ranging from 5 to 40, averaged across 100 replications). *r*: the estimate of Spearman correlation; *P:* the *P*-value of Spearman correlation test.    **R3 Fig. Separate scatter plots for different *c* values of MSE reduction and prediction *R^2^* when applying WC-correction.** The reduction in MSE was calculated as the MSE of MR analysis without WC-correction minus the MSE of MR analysis with WC-correction. Each dot denotes a parameter setting (averaged across 100 replications). *r*: the estimate of Spearman correlation; *P:* the *P*-value of Spearman correlation test.  We also checked the performance of the prediction *R^2^* in the real data application. It was observed that no matter what *c* value was chosen, the number of traits reaching the lowest *R^2^* remained fairly consistent, ranging from 20 to 25 (except when the value of *c* was set as 40, no trait reached the lowest *R^2^*). Moreover, the Wilcoxon signed rank test revealed that between any two selection of *c*, the prediction *R^2^* for all analyzed traits has no difference (all with *P* > 0.3, **R4 Fig**). These results suggest that *R^2^* may not play a critical role in the selection of *c* in the real-world application.    **R4 Fig. Results when applying WC-correction under different *c* values in the real data application. (A) Number of traits reaching the lowest prediction *R^2^* for each selection of *c.* (B) Boxplot of the prediction *R^2^* across 180 traits under different selection of *c*.** The *P*-values for each pair of boxplots from the Wilcoxon signed rank test are shown in the upper table, all of which are greater than 0.3.  Overall, we discovered that it is very difficult to find a highly persuasive and perfect solution to select the parameter *c* in the real data application where the causal relationship is unknown. Given that MR is concerned with inferring hidden causal associations rather than prediction of the outcome, traditional metrics like *R^2^* and MSE cannot be well applied in real data settings. Consequently, most proposed MR methods are assessed through ideal simulations using canonical metrics, and/or testing the sensitivity or repeatability of the results in the real data application. For instance, in the MR literature [4], the parameter "σ" was only evaluated for its robustness in the rounding selections in the real application. In the MR literature [5], the selection of the prior distribution for "*q*" was only examined through simulations. Furthermore, we believed that the reason for choosing *c* as 20 is sufficiently reliable as follows:  (1) Setting *c* as 20 consistently proves to be the optimal choice in simulation. When *c*=20, it reaches the highest ΔMSE, and the index value of ΔMSE at *c*=20 was significantly higher than that at *c*=15 and 25 across all 48 parameter settings (*P*=2.7×10^-5^ for *c*=15, and *P*=0.036 for *c*=25, **Fig 3B**).  (2) We have provided sufficient simulations and shown that the WC-correction procedure under *c*=20 can further enhance the MR results in minimizing MSE (including bias and variance), controlling type I error rate, and improve power under a variety of sceneries, including different heritability, confounding factors strength, patterns of causal effects, correlating structure of instrumental variants and *P*-value thresholds employed for instrumental SNPs selection (**Figs 2 and 3; S2-S4 Tables; S2, S3, S16 and S17 Figs**)  (3) We have validated in simulations that the improvement of MR-PL through WC-correction procedure is not specific to a single choice of *c*, but is robust across a gird of neighboring values across diverse parameter settings (**S10-S15, S18-S21 Figs**).  (4) In the real data application, most (18 out of 26) of these predicted causal associations could be supported by previous literature (**S13 Table**), suggesting the high reliability of the results generated by our approach.  (5) In the real data application, MR results with WC-correction are robust to the selection of *c* around 20. High positive correlation of IDP-trait *β*-maps between the results of *c* at 20 and 15/25 were observed (*r* = 0.831/0.851, all with *P* < 5×10^-12^, Spearman correlation), and the associations between IDPs and smoking, blood cell composition and eating behaviors still remained nominal significant (*β* ≠ 0 and *P* < 0.05).  (6) We have added discussion on the limitation and caution readers that our WC-correction is not optimal and is dependent on multiple factors (**Lines 561-566**), and the best choice to select instrument from another independent dataset. Nonetheless, the simple WC-correction procedure presented here only requires the GWAS *P*-value of SNPs and has shown generally improved MR results across a variety of scenarios including different heritability, confounding factors strength, patterns of causal effects, correlating structure of instrumental SNPs and *P*-value thresholds for IV selection, thereby it can be fast and conveniently applied without adding any additional computational complexity. In the future, more accurate WC-correction methods tailored to MR with high dimension and high correlation situations can be developed. With rapid accumulation of the biobank-scale datasets and GWAS summarized data, the WC bias issue could be entirely avoided by selecting IVs based on a different dataset [6].  In light of the above circumstances, we removed this part of selecting *c* with the lowest prediction *R^2^* and recommended to choose *c* as 20 as well as testing the robustness at its neighboring selections as follows. |
| Excerpt 2.2.1 (main text) | **Lines 550-555:**  Here, we recommend the following selection of *c* in the real data application:  Step 1. Determine whether the WC-correction is required; if the IV selection is based on a different sample from that used for MR analysis, then the correction is not required;  Step 2. Find the grid search range for the parameter *c*. Here, we strongly recommend to select *c* as 20 based on our results;  Step 3: Examine the robustness of MR results at neighboring values of the selected *c* (e.g., 15 or 25). |

**REF 2.2.2**

| *Referee Comment* | *Second, for the authors' response 2.2.4, transformation between P-values and Z-statistics is valid given the usual Z-score=beta_hat/se_hat which (asymptotically) follows standard normal under null hypothesis. However, my point is that when the original Z-score is transformed based on the proposed method, I don't think it still follows the standard normal that the authors use to calculate the corrected p-value.* |
| --- | --- |
| Author Response | We sincerely appreciate your thoughtful consideration. Basically, the proposed method here was informed by the previous study [7], which is a heuristic approach rather than one based on rigorous grounded theory. To make things more easy, here we assume that the transformed *z*-score after correction follows the standard normal distribution. Then we could re-select the instrumental SNPs based on the pre-specified *P*-value threshold (e.g., 5×10^-8^). We believe that, although this *z*-score transformation step may not be very strict in theory, it is intuitive and easily accessible to readers without any unnecessary complication. Furthermore, it is important to notice that extensive simulations have been carried out to validate the efficiency of proposed WC-correction. These simulations demonstrated notable improvements in minimizing MSE (comprising both bias and variance), effectively controlling the type I error rate across a wide range of scenarios. All these results indicate the practicality and efficiency of the proposed method.  In response to the reviewer's feedback, we have made some revisions in the manuscript. Specifically, we have weakened the narration in **Fig 1** by removing the transformation formula, and also stated the assumption-based transformation more explicitly in **Methods** (**Lines 735-737**). We thank the reviewer's dedication to enhancing the clarity of our methodology.    **Fig 1. Schematic showing the MR-PL method.** |
| Excerpt 2.2.2 | **Lines 735-737:**  Notably, here we assume that the shrank *z*-score follows the standard normal distribution, thus enabling re-selecting instruments based on the corresponding pre-specified *P*-value threshold. |

**REF 2.3 – Conditional F-statistic**

| *Referee Comment* | *In the calculation of the* *conditional F-statistics, could the authors clarify how they calculated for MR-PL? Step 1 says each exposure is regressed on the whole genotype matrix, but I assume in MR-PL the Xhat is calculated based on the PLS? In the real data analysis, all condition F-statistics are smaller than 2, are these calculated based on OLS or PLS?* |
| --- | --- |
| Author Response | Thank you for your suggestion, and we want to make it more clear here. In our manuscript, for both simulation and real data analysis, all conditional F-statistics were calculated based on the ordinary least square (OLS) method, where we fully followed the calculation steps of conditional F-statistics outlined in the original literature [8]. The underlying theoretical theory of weak instrument is systemic and pretty complex [8,9], and the steps to calculate the F-statistic for evaluation are merely the ultimate results. Also, few MR literatures could we found that calculated the conditional F-statistic with a regression method other than OLS. In light of these, we thought that we cannot simply transfer to calculate $\hat{X}$ based on PLS in MR-PL as we lack sufficient evidence and theoretical basis for doing so. Thus, we would like to persist the original calculation steps of conditional F-statistics for the sake of conservatism. In the revised manuscript, we have clarified that OLS was used for the regression steps in the calculation of conditional F-statistics as follows. |
| Excerpt 2.3 | **S1 text:**  **Calculation of the conditional F-statistic**  Usually, the Sanderson-Windmeijer conditional F-statistic (*F_sw_*) is used to tested for the weak instruments problem in one-sample multivariable MR, and a *F_sw_* greater than the rule-of-thumb value of 10 means there is sufficient conditional instrument strength [2-4]. The calculation steps of *F_sw_* can be briefed as follows:  Step 1. Each exposure $X_{i}$ (1≤*i*≤*K*) is regressed on the whole set of genotype matrix $G$ through the ordinary least square method (OLS), and the predicted value of each exposure $\hat{X}_{i}$ (1≤*i*≤*K*) is calculated;  Step 2. Each exposure $X_{i}$ is regressed on all the other predicted exposures through OLS to yield the residual term $\varepsilon_{i}=X_{i}-\delta_{1}\hat{X}_{1}-...-\delta_{k}\hat{X}_{k(k\neq i)}-...-\delta_{K}\hat{X}_{K}$;  Step 3. The residual term corresponding to each exposure is then regressed on the whole set of genotype matrix $G$ through OLS, and the conditional F-statistic *F_sw_* is obtained as the freedom adjusted F-statistic (*F*) in this regression as *F_sw_*= *F*×*M*/(*M*-*K*+1), where *M* denotes the total number of instrumental SNPs. |

**REF 2.4 – Figure drawing issue**

| *Referee Comment* | *Minor:*  *I'm not sure why a 95% line is plotted in the power plot. p < 0.05 only tells you control type-I error at 5%.* |
| --- | --- |
| Author Response | Thank you for this suggestion. We agree with you that the 95% line potted in the power plot is unnecessary and may cause confusion. In the revised manuscript, we have removed the lines in all figures when showing the power results in **S9, S14 and S15 Figs**. |
| Excerpt 2.4 | Not applicable. |

**REF 2.5 – Issue of including BMI as the covariate**

| *Referee Comment* | *Minor:*  *In the real data analysis, when performing GWAS on IDP, why the authors also adjusted for BMI? How would the results be different if not adjusting for BMI?* |
| --- | --- |
| Author Response | Thank you for the suggestion. There are several reasons for adjusting for BMI in the real data application. First, BMI is a potential confounding factor having been reported for its association with brain imaging measurement [10,11], and is always regressed out as covariates in relevant imaging-trait association studies [3,12-14]. Second, the Brain Imaging Documentation of UK Biobank (http://www.ukbiobank.ac.uk) also recommends to consider BMI as confound variables when working with imaging data. Third, in line with the suggestion from reviewer #3 in the first round of revision, BMI should be adjusted given its potential association with many traits that served as the outcome in MR analysis, including blood cell composition, physical measurements, smoking and socio-demographics [15-17].  We also repeated the real data analysis without including BMI as a covariate. The global tendency of causal estimations still remained consistent, where significant correlation of IDP-trait *β*-maps was observed (*r* = 0.992, *P* < 5×10^-16^, Spearman correlation). For the 26 IDP-trait pairs presented in **Table 1**, 20 of them still stood with a non-zero causal effect estimate at *P* < 0.05 without including BMI as a covariate (**R1 Table**). The associations between IDPs and smoking, blood cell count and eating behaviors could be still identified. However, the causal associations between uncinate fasciculus and eosinophill percentage, anterior limb of internal capsule and high light scatter reticulocyte count, and superior fronto-occipital fasciculus and pack years of smoking disappeared after removing BMI as a covariate. These results reveal that whether including BMI as a covariate might cause slight difference in the causal associations for traits such as blood cell composition, smoking and socio-demographics, but the main conclusion and overall tendency are still the same as before, which is expected.  **R1 Table.** **MR results for the 26 IDP-trait pairs presented in Table 1, with and without adding BMI as the covariate.** The ‘Original results’ columns are the same as those shown in **Table 1**. The results with a non-zero causal effect estimate at *P* < 0.05 after removing BMI as a covariate were labeled as blue.   |
| Excerpt 2.5 | Not applicable. |

*Reviewer #3: I have no further comments to the authors. I think they did not get the suggestion of testing BMI LDL and HDL as exposures as outcomes and CVD as exposure to have a positive control, but I don't want to push the subject further.*

**REF3 – Response to REF3:** We sincerely thank you for taking the time to review our work. During the revision process, our primary focus is on improving the methodology and optimizing the validation process. Your suggestions for biological discoveries will be considered further in our future research.

**References**

1.	Zhang, C.H. and S.S. Zhang, *Confidence intervals for low dimensional parameters in high dimensional linear models.* Journal of the Royal Statistical Society, Series B. Statistical Methodology, 2014.

2.	Dezeure, R., et al., *High-dimensional inference: confidence intervals, p-values and R-software hdi.* Statistical science, 2015: p. 533-558.

3.	Yang, A., et al., *Longer screen time utilization is associated with the polygenic risk for Attention-deficit/hyperactivity disorder with mediation by brain white matter microstructure.* EBioMedicine, 2022. **80**: p. 104039.

4.	Zuber, V., et al., *Selecting likely causal risk factors from high-throughput experiments using multivariable Mendelian randomization.* Nat Commun, 2020. **11**(1): p. 29.

5.	Morrison, J., et al., *Mendelian randomization accounting for correlated and uncorrelated pleiotropic effects using genome-wide summary statistics.* Nat Genet, 2020. **52**(7): p. 740-747.

6.	Burgess, S., et al., *Guidelines for performing Mendelian randomization investigations.* Wellcome Open Res, 2019. **4**: p. 186.

7.	Shi, J., et al., *Winner's Curse Correction and Variable Thresholding Improve Performance of Polygenic Risk Modeling Based on Genome-Wide Association Study Summary-Level Data.* PLoS Genet, 2016. **12**(12): p. e1006493.

8.	Sanderson, E. and F. Windmeijer, *A weak instrument [Formula: see text]-test in linear IV models with multiple endogenous variables.* J Econom, 2016. **190**(2): p. 212-221.

9.	Stock, J.H. and M. Yogo, *Testing for weak instruments in linear IV regression*. 2002, National Bureau of Economic Research Cambridge, Mass., USA.

10.	Bobb, J.F., et al., *Cross-sectional and longitudinal association of body mass index and brain volume.* Hum Brain Mapp, 2014. **35**(1): p. 75-88.

11.	Sui, S.X. and J.A. Pasco, *Obesity and Brain Function: The Brain–Body Crosstalk.* 2020. **56**(10): p. 499.

12.	Cheng, W., et al., *Sleep duration, brain structure, and psychiatric and cognitive problems in children.* Mol Psychiatry, 2021. **26**(8): p. 3992-4003.

13.	Shen, C., et al., *What Is the Link Between Attention-Deficit/Hyperactivity Disorder and Sleep Disturbance? A Multimodal Examination of Longitudinal Relationships and Brain Structure Using Large-Scale Population-Based Cohorts.* Biol Psychiatry, 2020. **88**(6): p. 459-469.

14.	Wang, H., et al., *Severe nausea and vomiting in pregnancy: psychiatric and cognitive problems and brain structure in children.* BMC Med, 2020. **18**(1): p. 228.

15.	Church, T.S., et al., *Relative associations of fitness and fatness to fibrinogen, white blood cell count, uric acid and metabolic syndrome.* Int J Obes Relat Metab Disord, 2002. **26**(6): p. 805-13.

16.	Hudson, L.D., et al., *Evidence for contemporary arterial stiffening in obese children and adolescents using pulse wave velocity: A systematic review and meta-analysis.* Atherosclerosis, 2015. **241**(2): p. 376-86.

17.	Wang, P., et al., *Smoking and Socio-demographic correlates of BMI.* BMC Public Health, 2016. **16**: p. 500.
